# Development of a measure of knowledge and attitudes about obstructive sleep apnea for pediatric anesthesia (OSAKA-PedAn) and survey of knowledge and attitudes about pediatric obstructive sleep apnea among Italian anesthesiologists

**DOI:** 10.1186/s44158-025-00260-z

**Published:** 2025-07-01

**Authors:** Alessandro Simonini, Marco Cascella, Antonino Giarratano, Elena Giovanna Bignami, Giacomo Grasselli, Roberto Pedone, Diletta Costantini, Elisa Romagnoli, Alessandro Vittori

**Affiliations:** 1 Pediatric Anesthesia and Intensive Care Unit, Salesi Children’s Hospital, Ancona, Italy; 2 Anesthesia and Pain Medicine, Department of Medicine, Surgery and Dentistry “scuola Medica Salernitana”, University of Salerno, Baronissi, 84081 Italy; 3 Department of Anaesthesia, Intensive Care and Emergency Policlinico Paolo Giaccone, Palermo, Italy; 4 Division of Anesthesiology, Critical Care and Pain Medicine, Department of Medicine and Surgery, University of Parma, Parma, Italy; 5 Department of Anesthesiology Intensive Care and Emergency Fondazione IRCCS Ca’ Granda Ospedale Maggiore Policlinico, Milan, Italy; 6 Department of Psychology, University of Campania Luigi Vanvitelli, Caserta, Italy; 7 Department of Anesthesia, Critical Care and Pain Medicine, ARCO, Ospedale Pediatrico Bambino Gesù IRCCS, Rome, Italy

**Keywords:** Sleep-disordered breathing, Obstructive sleep apnea syndrome, Anesthesia, Pediatric anesthesia, Education, Tonsillectomy, Adenotonsillectomy, Bleeding, Pain, Opioid

## Abstract

**Background:**

Sleep-disordered breathing and obstructive sleep apnea syndrome are two diseases of relevant clinical and research interest, especially in the pediatric field. However, there are gaps in knowledge regarding these diseases.

**Methods:**

We performed a survey that was administered electronically, via the SurveyMonkey platform, to 15,000 Italian anesthesiologists registered on the SIAARTI mailing list for a period of 4 months (April–July 2021).

**Results:**

A total of 223 anesthesiologists completed the questionnaire (1.48%), 143 female and 79 male. Pediatric anesthetists generally responded more correctly than the general anesthesiology population.

**Conclusion:**

The topic of pediatric OSA is a topic on which to invest in training of anesthesiologists, and the OSAKA-PedAn questionnaire is a valid tool to test the level of basic competence and that achieved.

**Supplementary Information:**

The online version contains supplementary material available at 10.1186/s44158-025-00260-z.

## Background

Sleep-disordered breathing (SDB) is a common problem in children that can affect medical, psychological, and developmental aspects of the child [1]. The most common consequences of SDB can include enuresis, daytime sleepiness, headaches, behavioral changes from restlessness to aggression, poor school performance, and more [2–12].

Prevalence estimates of SDB are highly variable (from 0.7 to 13.0%) and this is likely due to the fact that different diagnostic techniques and criteria have been adopted in different studies [7, 9–11]. A study by Bixler et al. observed a prevalence of 1.2% for moderate SDB, 25.0% for mild SDB, and 15.5% for primary snoring [13].

Obstructive sleep apnea syndrome (OSAS) is a public health problem affecting 1% to 6% of all children, up to 59% of obese children, and 2% to 24% of adults [13–15]. Obstructive sleep apnea syndrome (OSAS) in the pediatric population is characterized by intermittent partial or complete obstruction of the upper airways, resulting in hypoxia, hypercapnia, increased respiratory effort, significant changes in intrathoracic pressure, and fragmentation of rapid eye movement (REM).

### Sleep with frequent awakenings

The prevalence reported in the most recent literature varies from 1 to 5%, depending on the population studied and on the diagnostic criteria used [16]. The peak prevalence occurs between 2 and 8 years of age [17, 18].

Few data are available on risk factors related to OSAS, especially in the pediatric age [19]. Pediatric OSA has a multifactorial etiology with some well-defined risk factors (obesity, adenoid, and/or adeno-tonsillar hypertrophy, allergic rhinitis, craniofacial malformations, proinflammatory states). The identification of risk factors in each patient is essential for a careful planning of the perioperative pathway, since OSA can increase the risk of anesthetic complications also during non-operating room anesthesia (NORA) procedures [20].

The American Society of Anesthesiologists (ASA) has recently published an update of the guidelines for the perioperative evaluation and management of these patients because airway obstruction during sleep causes episodic desaturation and hypercapnia that over time can determine cardiovascular dysfunctions such as pulmonary hypertension and left ventricular hypertrophy [21–23]. Repeated episodes of desaturation can modify the mu opioid receptors, determining, in children with OSA, a greater sensitivity to this category of drugs with potential risk of overdose with the administration of a standard dose [24–27].

We hypothesized an insufficient knowledge and awareness of pediatric OSA among Italian Anesthesiologists who care for children.

The Obstructive Sleep Apnea Knowledge and Attitudes (OSAKA) questionnaire [27] has been developed and successfully used in adults; however, OSA in children has different characteristics. The OSAKA-KIDS questionnaire was developed to study pediatric OSA [26], but it does not explore aspects of anesthetic relevance.

The Ostructive Sleep Apnea Knowledge and Attitudes in Pediatric Population (OSAKA-PedAn) questionnaire, presented here, was developed to assess the knowledge and attitudes of anesthesiologists regarding OSA specifically in the pediatric population. In this article, we describe the development of the OSAKA-PedAn questionnaire and the results of a pilot administration of the survey to Italian anesthesiologists.

## Methods

Ethical review and approval were waived for this survey, which did not collect sensitive personal or clinical data. According to Italian legislation for non-interventional studies (Ministerial Circular N. 6, 2 September 2002), ethics committee approval is only required if the study addresses issues related to prescribed medicinal products, where patient inclusion in therapeutic strategies is determined by normal clinical practice rather than the trial protocol.

The OSAKA-PedAn questionnaire is an Italian-language questionnaire consisting of 28 items that assesses the knowledge and attitudes of Anesthesiologists regarding pediatric OSA.

To establish the validity of the questionnaire, a panel of anesthesiologists with proven experience in the pediatric field, pediatric pulmonologists, pediatric neurologists, and representatives of patient associations was created by the SIAARTI Airway Management Study Group (GVA). The OSAKA-PedAn questionnaire was subjected to 3 Delphi rounds in which the panelists was asked to rate the relevance of each of the 28 items using a 4-point scale (1, not relevant; 2, slightly relevant; 3, relevant and 4, extremely relevant). Items were considered suitable for inclusion in the final questionnaire if at least 75% of the expert panel members rated individual items as 3 or 4. Modifications were made based on expert feedback.

The final questionnaire included 14 knowledge items and 9 attitude items. The knowledge domains include epidemiology, pathophysiology, symptoms, diagnosis, and treatment options of pediatric OSA (Appendix 1). Items were presented in a true-or-false format. “Don’t know” was included as a third response choice to minimize the effect of guessing and was scored as an incorrect response. Additionally, 9 attitude items were included regarding the importance of OSA as a clinical disorder in children and stratification of anesthetic risk, the importance of identifying the disorder, pharmacological appropriateness in performing general anesthesia, management of the postoperative period, the importance of Team Work and multidisciplinary management of confirmed cases. These items, taken from the attitude items on the OSAKA questionnaire for adults, were reformulated to specifically investigate childhood OSA. Physicians were asked to rate their level of agreement with each of the 5 items using a 5-point Likert scale ranging from 1 (strongly disagree) to 5 (strongly agree). The OSAKA-PedAn questionnaire also included questions on age, sex, year of specialization in anesthesiology, type of hospital activity (Pediatric Hospital, Adult Hospital with Pediatric Department, Adult Hospital with sporadic pediatric activity, Hospital exclusively for adults), and number of pediatric anesthetic procedures per year (in order to investigate whether greater experience in the pediatric field translates into a higher cultural level). The questionnaire was administered electronically, via the SurveyMonkey platform, to 15,000 Italian Anesthesiologists registered on the SIAARTI mailing list for a period of 4 months (April–July 2021). Participants were asked to consent to provide their data and participate in the survey. All questionnaires were collected by April 30, 2021 by the scientific secretariat of SIAARTI and sent to Statistical Laboratory for statistical analysis. No compensation was offered for participation in the survey, either to the respondents or to the researchers.

### Statistical analysis

An analysis of the responses was carried out by aggregating them by “Experience” or by considering both seniority, and therefore distinguishing between Resident (1st–3rd year Resident and 4th–5th year Resident) and Specialist (Specialist 31–40, Specialist 41–50, Specialist 51–60, Specialist over 61), and by considering the number of pediatric sedations and/or anesthesia procedures performed in a year grouped into the following classes: 20–39, 40–149, 150–25, > 251. A further aggregation was considered in agreement with the hospital where the interviewee declared to carry out his/her work activity mainly (SP = Pediatric facility, SADP = Adult facility with pediatric department, SASAP = Adult facility with sporadic pediatric activity, SA = Facility exclusively for adults). For the questions that assess the interviewee’s knowledge of the study topic, a parametric analysis was performed to investigate the possible presence of a dependency relationship between the probability of answering correctly and the independent variables (Experience and Structure). The interviewee is asked to declare a number of pediatric sedations/anesthesia performed monthly. The response methods included classes of amplitude equal to 5 anesthesia procedures defining a total interval from 0 to over 50 sedations/anesthesia procedures. This information was coded in annual starting from the central value of each class and subsequently grouped into clusters as per study needs (20–39, 40–149, 150–250, > 251). With the aim of facilitating the interpretation of the result, following the coding, it was decided to merge the 40–99 class with the 100–149 class.

For questions requiring a Likert scale response method, an indicator is proposed that summarizes the information from the frequency distributions, returning a more robust result that allows a more immediate and less distorted comparison between different levels of Experience (Seniority–Number of sedations/anesthesia procedure), types of hospital. This tool therefore allows us to understand the degree of agreement expressed by the interviewees towards a judgment of the Item that goes from “Not at all” to “Very much.” There is maximum agreement towards the worst judgment when the indicator is equal to 0, maximum agreement towards the best judgment when it is equal to 1 and 0.5 expresses the disagreement between the interviewees. Clinical trial number is not applicable.

## Results

The study sample consisted of 223 anesthesiologists who completed and returned the questionnaires, with a response rate for this study of 1.48% (223/15000).

Demographic data, professional profile, and number of years of experience are reported in Table 1.

**Table 1:** Main characteristics of anesthesiologists who completed the OSAKA-PedAn questionnaire

	**Value**
**Age (years)**	43.5 ± 10.8
**Sex**	
Female	143 (64.4%)
Male	79 (35.6%)
**Type of hospital**	
Pediatric Hospital	43 (19.3%)
Adult Facility With Pediatric Department	64 (28.7%)
Sporadic Pediatric Activity	80 (35.9%)
Facility Exclusively For Adults	35 (15.7%)
Other	1 (0.4%)
**Professional experience**	
Specialist over 61	19 (8.5%)
Specialist 51–60	32 (14.3%)
Specialist 41–50	75 (33.6%)
Specialist 31–40	55 (24.7%)
Resident 4°–5° year of course	18 (8.1%)
Resident 1°–3° year of course	24 (10.8%)
**Sedations/anesthesia procedures per year**	
20–39	121 (54.3%)
40–149	26 (11.7%)
150–250	23 (10.3%)
> 250	53 (23.8%)

All results are reported in Appendix 2.

**Fig. 1:**
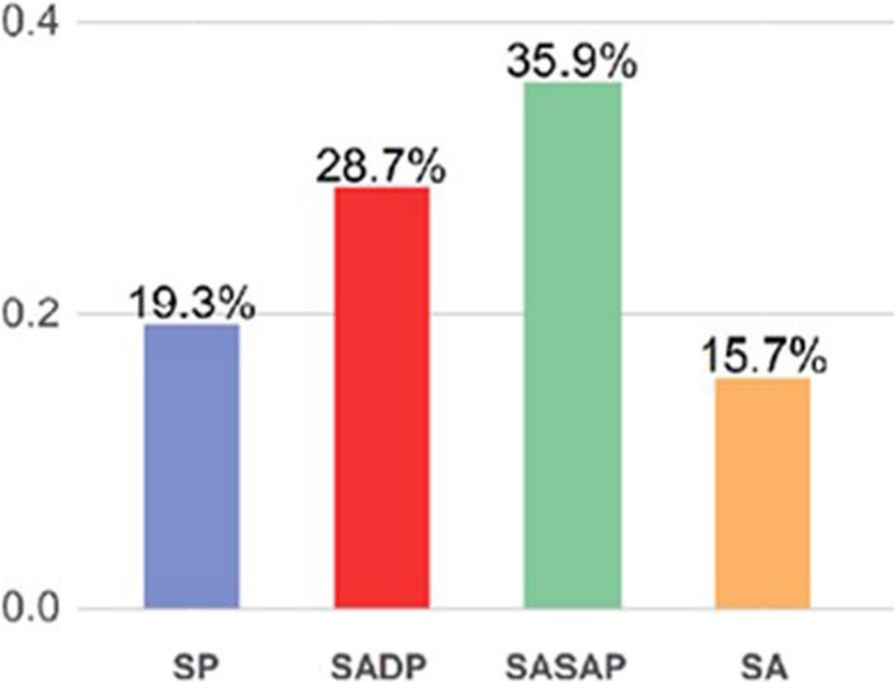
Hospital type of interviewees

Most of the interviewees declared to work in public facilities: University Hospital (40.4%), Hospital (37.2%), Public Istituto di Ricerca e Cura a Carattere Scientifico (IRCCS) (8.1%). A much lower percentage of the interviewees declared to work in private facilities: Private IRCCS (7.2%), Conventioned Hospital (3.1%), Private Hospital (1.3%), Other facilities (2.7%). Most of the interviewees stated that they work in an adult facility that also provides pediatric services and almost all stated that in their region there is a pediatric referral center with a Pediatric Intensive Care Unit (Fig. 1).

**Fig. 2:**
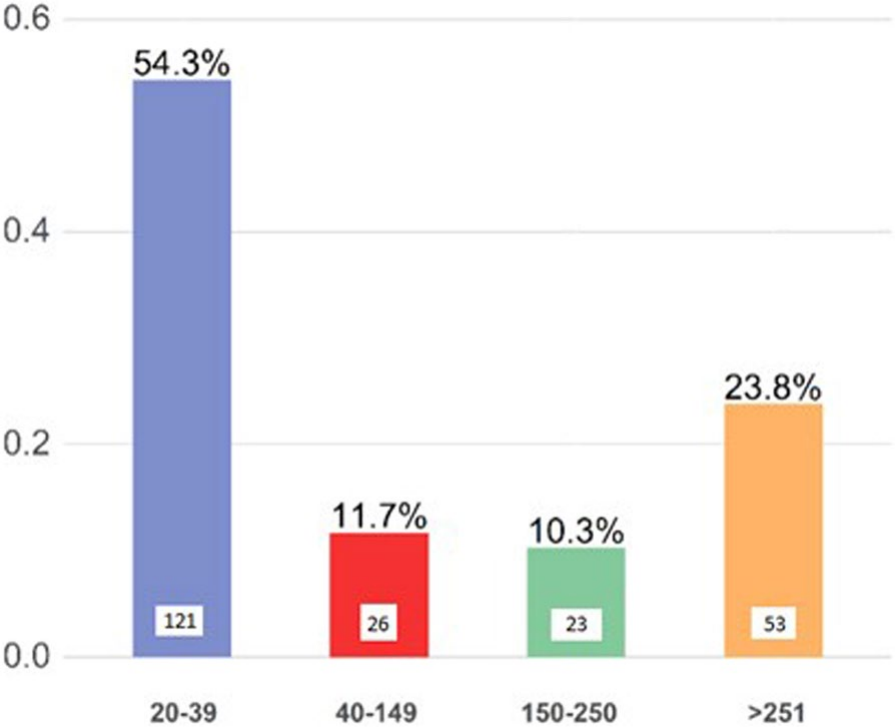
Volume of pediatric activity of interviewees

With regard to specific experience, 54.3% stated that they perform between 20 and 39 pediatric sedations/anesthesia procedures per year (Fig. 2).

The interviewees were asked to express a preference on the need to produce a “Good Clinical Practices” document on pOSAs.

Anesthesiologists who have performed a number of anesthesia procedures per year between 20 and 39 and between 40 and 149 seem to be more interested in the production of “Good Clinical Practices on pOSAs”.

**Fig. 3:**
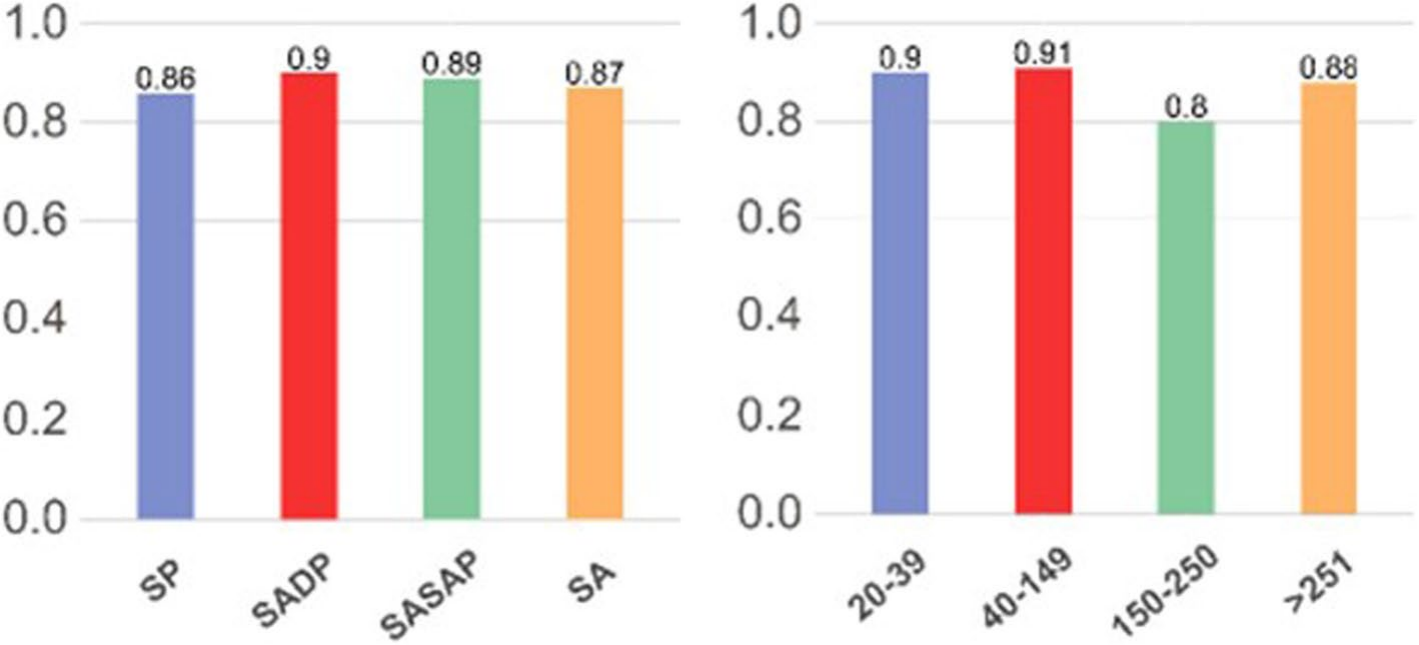
Perceived need for the production of Good Clinical Practices

Figure 3 summarizes the results obtained regarding the perceived need for the production of Good Clinica Practices.

### Epidemiology

Respondents were asked if the incidence of pOSAs is 0.1–0.5%; 65 respondents (29%) answered correctly, 64 respondents (29%) answered incorrectly, and 94 respondents (42%) stated that they did not know the answer. Regarding the facility and the experience, there is no relationship of dependence (*p* > 0.05).

Respondents were asked if 10–15% of children have Sleep-Related Breathing Disorders; 21 respondents (9%) answered correctly, 51 respondents (23%) answered incorrectly, and 151 respondents (68%) stated that they did not know the answer. Regarding the facility and the experience, there is no relationship of dependence (*p* > 0.05).

Respondents were asked whether pOSAs is more common in females; 131 respondents (60%) answered correctly, 21 respondents (9%) answered incorrectly, and 68 respondents (31%) said they did not know the answer. Regarding the facility and the experience, there is no relationship of dependence (*p* > 0.05).

The percentage of anesthesiologists who declare to perform more than 251 anesthesia procedures in a year who answered the questionnaire correctly is 0.27 times lower than the percentage of Anesthesiologists who declare to perform 20–39 anesthesia procedures per year who answered the question correctly (*p* 0.03).

### Physiology

Interviewees were asked whether craniofacial malformations can cause OSAs; 213 interviewees (96%) answered correctly, none answered incorrectly, and 10 interviewees (4%) stated they did not know the answer.

Interviewees were asked whether the most frequent cause of OSAs in the age group > 8 years is adeno-tonsillar hypertrophy; 39 interviewees (18%) answered correctly, 50 interviewees (22%) answered incorrectly, and 134 interviewees (60%) stated they did not know the answer.

### Symptomatology

Interviewees were asked whether enuresis can be a symptom of pOSAs; 141 interviewees (63%) answered correctly, 16 interviewees (7%) answered incorrectly, and 66 interviewees (30%) stated that they did not know the answer. Respondents were asked whether a child with OSA can be hyperactive; 119 respondents (53%) answered correctly, 24 respondents (11%) answered incorrectly, and 80 respondents (36%) said they did not know the answer.

Respondents were asked whether compensated respiratory acidosis may suggest pOSAs; 175 respondents (79%) answered correctly, 11 respondents (5%) answered incorrectly, and 37 respondents (17%) stated that they did not know the answer.

### Diagnosis

Respondents were asked whether polysomnography (PSG) is the ideal test for the diagnosis of pOSAs; 205 respondents (92%) answered correctly, 1 respondent (0.4%) answered incorrectly, and 17 respondents (7.6%) stated that they did not know the answer.

Respondents were asked whether an accurate ENT examination can exclude the diagnosis of pOSAs; 13 respondents (59%) answered correctly, 32 respondents (14%) answered incorrectly, and 60 respondents (27%) stated that they did not know the answer.

Respondents were asked whether an apnea/hypopnea index of 0–5 on PSG is normal in children; 38 respondents (17%) answered correctly, 52 respondents (23%) answered incorrectly, and 133 respondents (60%) stated that they did not know the answer.

### Therapeutic options

Interviewees were asked whether, unlike adults, CPAP is never useful in pOSAs; 106 interviewees (48%) answered correctly, 28 interviewees (13%) answered incorrectly, and 89 interviewees (40%) said they did not know the answer.

Interviewees were asked whether adenotonsillectomy is mandatory in moderate/severe pOSAs; 42 interviewees (19%) answered correctly, 53 interviewees (24%) answered incorrectly, and 128 interviewees (58%) said they did not know the answer.

### Importance of the clinical disorder and stratification of the anesthetic risk

Item 1. “severe pOSAs can cause pulmonary hypertension, therefore a cardiological evaluation is indicated”: all the anesthesiologists interviewed agree with the proposed statement.

Item 2. “Children affected by OSAs present a greater anesthetic risk and should be referred to centers equipped with pediatric intensive care”: all the anesthesiologists would seem to agree with the proposed statement. All the anesthesiologists who carry out their activity in a pediatric facility would seem to agree with the proposed statement.

### Importance of identifying the disorder

Item 3. “Children affected by OSAs undergoing adenotonsillectomy require special postoperative precautions”: specialists over 41–50 and specialists 51–60 would seem to agree in adopting special precautions in children affected by OSAs undergoing adenotonsillectomy. Anesthesiologists working in a pediatric facility would seem to agree on adopting particular precautions in children affected by OSA undergoing adenotonsillectomy.

### Pharmacological appropriateness

Item 4. “Opioid analgesics can be safely used at standard dosages in the intraoperative period”: Anesthesiologists seem to have a discordant opinion on the use of opioid analgesics in the intraoperative period.

Item 5. “In children with OSA, it is preferable not to use inhaled anesthetics”: the interviewees seem to have a discordant opinion on the use of inhaled anesthetics in children with OSA.

Item 6. “In children with OSA, it is preferable to avoid neuromuscular blockade”: anesthesiologists seem to have a discordant opinion on avoiding neuromuscular blockade in children with OSA.

### Post-operative/post-procedure management

Item 7. “In case of suspected pOSAs, it is advisable to monitor vital parameters for 24 h after a procedure under sedation/AG”: all specialists, unlike the specialist over 61, would seem to agree to monitor vital parameters for 24 h after a procedure under sedation/AG in case of suspected pOSAs.

Item 8. “Postoperative analgesia with opioids should include reduced dosages in case of moderate/severe pOSAs”: all specialists, unlike the specialist over 61, would seem to agree in monitoring vital parameters for 24 h after a procedure under sedation/AG in case of suspected pOSAs.

### Team work and multidisciplinary management

Item 9. “All specialists would seem to agree to put team work and multidisciplinary management into practice for the management of post-operative/post-procedure. All anesthesiologists working in the different types of hospitals taken as reference would seem to agree to put team work and multidisciplinary management into practice for the management of post-operative/post-procedure.

Interviewees were asked to express a preference on the topics related to pOSAs, of interest for further training.

The specialist compared to the resident seems to be more interested in training on risk stratification in pOSAs. The interviewees who work in a facility with a pediatric department or in pediatric facilities seem to be more interested in training on risk stratification in pOSAs compared to those who work in an adult facility.

All the anesthesiologists seem to be interested in training on the treatment and management of pOSAs.

## Discussion

As far as we know, this is the first survey among anesthesiologists aimed at exploring the level of knowledge and attitudes on pediatric OSA.

With the major limitation related to the small sample size (only 1.48% of the specialists invited to participate responded), the knowledge of pediatric OSA among the Italian anesthesiologists involved in this survey seems to be insufficient. The correct answers were 54.1%, the incorrect answers 12.9%, and the abstentions 32.2%; considering that abstention is equivalent to the awareness of not knowing the topic, it can reasonably be said that almost half of the participants showed poor knowledge of pediatric OSA.

The greatest cultural gaps are in topics such as the epidemiology of OSA in children (correct answers 32.6%) and therapeutic options (correct answers 33.5%). The diagnostic process is also not sufficiently known (correct answers 56%) while the clinical suspicion, related to the knowledge of the symptoms, shows greater awareness among Anesthesiologists (correct answers 65%).

With regard to epidemiological knowledge, it was observed that “pediatric” anesthesiologists (> 250 pediatric anesthesia procedures per year) provided an incorrect answer with a frequency 27% higher than “non-pediatric” Anesthesiologists (< 250 anesthesia procedures per year); this data, however, appears to be of little clinical relevance.

With regard to the culture of the physiopathology of OSA in the pediatric population, it is interesting to note that “pediatric” anesthesiologists correctly answer in a percentage 30% higher than “non-pediatric” anesthesiologists to the question relating to the most frequent cause of OSAS in the pediatric population over 8 years of age. In fact, there are 2 peaks of incidence of OSA in children without cranio-facial anatomical alterations or syndromes [28, 29] where, in the first, the main cause is represented by adeno-tonsillar hypertrophy (children between 2 and 8 years of age) while over 8 years of age the main cause is represented by obesity [30].

Regarding the knowledge of the pathognomonic symptomatology of OSA in children, “pediatric” anesthesiologists correctly answered 14% more than “non-pediatric” anesthesiologists to the question about hyperreactivity as a potential symptom of OSA.

It is interesting to note that knowledge about the diagnostic parameters of the polysomnographic examination (Apnea/Hypopnea Index—AHI) is poor in the entire population under study (correct answers 17%). This is important because polysomnography, and specifically the AHI parameter, allows both diagnosis and staging of the disease, a fundamental element for planning the perioperative period and assessing the anesthetic risk.

The topic related to therapeutic options is poorly known by Italian Anesthesiologists, especially with regard to the potential role of cPAP in pediatric OSA (correct answers 33.5%). In this case, it appears that doctors in training possess superior knowledge compared to their more experienced colleagues, in fact the percentage of incorrect answers in anesthesiologists over 61 is 91% higher than that of specialists in the 1st–3rd year of training. analyzing the work environment, our work highlights how anesthesiologists working in adult facilities provided a percentage of incorrect answers 84% higher than their colleagues working in pediatric facilities.

Regarding the attitudes of anesthesiologists in the management of pediatric patients with OSA, our work highlights how there is agreement on the need to manage these patients preferably in pediatric centers and to adopt particular precautions in anesthetic management; however, apparently in contradiction to this, a relative discordance is highlighted regarding the administration of opioid drugs, in the intraoperative period, without specific considerations and at the standard dosage expected for any other pediatric patient.

A discordant opinion, among anesthesiologists, is also highlighted regarding the use of inhaled anesthetics and muscle relaxant drugs. Probably, these attitudes reflect indications from literature which are not always univocal [31–34].

All specialists, unlike the specialist over 61, would seem to agree with monitoring vital parameters for 24 h after a procedure under sedation/general anesthesia in case of suspected OSAs.

We noted a positive correlation between knowledge scores and clinical attitude, and this suggests that knowledge of the problem and related risk increases the propensity for prudential attitudes, favoring correct perioperative planning with consequent reduction of risk and increase in quality of care.

This work suggests, in agreement with the available literature, that an adequate process of acquisition and maintenance of skills in pediatric anesthesiology, as well as activity in centers dedicated to the pediatric population is the basis of safety in pediatric anesthesia, especially in OSA patients who present a higher perioperative risk [35].

Airway obstruction represents a critical issue in anesthetic management and must be identified before induction of general anesthesia, to define the anesthetic risk and plan for the possibility of having to manage a difficult airway. General anesthesia mimics physiological sleep and during the induction phases, there is always a decrease in pharyngeal muscle tone which requires postural adjustment of the head and neck in order to restore airway patency [36]. Given the increasing use of drug-induced sleep endoscopy (DISE) in diagnostic and therapeutic planning for children with OSA, this should be an acquired skill for anesthesiologists.

The DISE technique involves an assessment of the upper airway using a flexible endoscope through which the airway is explored while patients are in a pharmacologically induced sleep-like state.

The consequences of persistent OSA, especially if severe, such as metabolic and cardiocirculatory alterations, make the patient more fragile. All this makes it important that this condition be investigated and made known to the anesthetists who will have to subject the patient to sedation or general anesthesia [37]. Equally important for the anesthesiologist is to know the severity of the OSA for the choice of drugs to use in maintaining the correct sedation/anesthesia plan, also in function of the awakening and postoperative/postprocedural surveillance [38–40].

Furthermore, the anesthesiologist is increasingly assuming a central role in perioperative medicine, with increasingly detailed planning that goes from the patient's admission to the hospital to his discharge, and also in pain therapy at home [41–44]. It must be remembered that correct pain management eliminates the first cause of unplanned admission in pediatric day surgery, and this is an exquisitely anesthetic skill [45, 46].

We have highlighted how there is awareness of cultural gaps with predisposition of anesthesiologists to a self-assessment process and request for further information on specific aspects.

### Limitations

This survey has a fairly low response rate (1.48%). This may be explained by several factors. The first and most important is that pediatric anesthesia is a subspecialty within the world of anesthesia. For this reason, many anesthesiologists who routinely care for adult patients may have decided not to participate in the survey. There is another quota of professionals who may not have responded because they work in the field of critical care or pain medicine. All this certainly represents a limitation. However, it is the first time that a survey of this kind has been conducted in Italy by SIAARTI, and it can be a good baseline for future information and training programs. For example, given the increasing use of DISE in diagnostic and therapeutic planning for children with OSA, this should be an acquired skill for anesthesiologists.

The DISE technique involves an assessment of the upper airway using a flexible endoscope through which the airway is explored while patients are in a pharmacologically induced sleep-like state. However, a universally accepted anesthesia protocol for the correct performance of sleep endoscopy has not yet been agreed upon by experts, in fact there are few studies comparing different anesthesia regimens used for pediatric DISE [47–49].

## Conclusions

Pediatric OSA is a topic of interest for anesthesiologists who work with children and further research is desirable to ensure quality care for children who suffer from this syndrome and are exposed to a greater risk of anesthetic complications, especially when anesthesia is conducted by anesthesiologists who are not usually involved in pediatrics; we believe that OSAKA-PedAn can be a useful tool to investigate the level of knowledge of pediatric OSA among Anesthesiologists and provide information about the training needs of professionals.

In our opinion, it is necessary to continue the training path in relation to pediatric OSA and to strengthen the offer by preparing documents that can be support tools for daily practice.

## Supplementary Information

Supplementary Material 1. Appendix 1 OSAKA-PedAn Questionnaire.

Supplementary Material 2. Appendix 2.

## Data Availability

The datasets used and/or analyzed during the current study are available from the corresponding author on reasonable request.

## Abbreviations

ASA: American Society of Anesthesiologists
DISE: Drug-induced sleep endoscopy
GVA: SIAARTI Airway Management Study Group
IRCCS: Istituto di Ricerca e Cura a Carattere Scientifico
NORA: Non-operating room anesthesia
OSAKA: Obstructive Sleep Apnea Knowledge and Attitudes
OSAKA-PedAn: Obstructive Sleep Apnea Knowledge and Attitudes in Pediatric Population
OSAS: Sleep apnea syndrome
REM: Rapid eye movement
SA: Facility exclusively for adults
SADP: Adult Facility With Pediatric Department
SASAP: Sporadic pediatric activity
SDB: Sleep-disordered breathing
SIAARTI: Italian Society of Anesthesia, Analgesia, Resuscitation and Intensive Care
SP: Pediatric facility
