# Supplementary material for: Development of a measure of knowledge and attitudes about obstructive sleep apnea for pediatric anesthesia (OSAKA-PedAn) and survey of knowledge and attitudes about pediatric obstructive sleep apnea among Italian anesthesiologists
Source: J Anesth Analg Crit Care. 2025 Jul 1;5:39. doi: 10.1186/s44158-025-00260-z (PMC12219456; doi:10.1186/s44158-025-00260-z)
Supplement: Supplementary file 1 — Supplementary Material 1. Appendix 1 OSAKA-PedAn Questionnaire. [file 44158_2025_260_MOESM1_ESM.docx]

Appendix 1. OSAKA-PedAn Questionnaire

|  | | | **True** | **False** | **I don’t know** |  |  | |
| --- | --- | --- | --- | --- | --- | --- | --- | --- |
| **Section 1**  **Knowledge** | 1) Epidemiology | **1.** The incidence of pOSAs is 0.1-0.5% | **-** | **-** | **-** |  |  | |
|  |  | **2.** 10-15% of children have Sleep-Disordered Breathing | **-** | **-** | **-** |  |  | |
|  |  | **3.** POSAS is more common in females | **-** | **-** | **-** |  |  | |
|  | 2) Pathophysiology | **4.** Craniofacial malformations can cause OSAs | **-** | **-** | **-** |  |  | |
|  |  | **5.** The most common cause of OSA in the age group >8 years is adenotonsillar hypertrophy | **-** | **-** | **-** |  |  | |
|  |  | **6.** Upper airway muscle hypotonia during sleep contributes to pOSAs | **-** | **-** | **-** |  |  | |
|  | 3) Symptoms | **7.** Enuresis can be a symptom of pOSAs | **-** | **-** | **-** |  |  | |
|  |  | **8.** The child affected by OSA can be hyperactive | **-** | **-** | **-** |  |  | |
|  |  | **9.** Compensated respiratory acidosis may suggest pOSAs | **-** | **-** | **-** |  |  | |
|  | 4) Diagnosis | **10.** Polysomnography is the ideal test for the diagnosis of pOSAs | **-** | **-** | **-** |  |  | |
|  |  | **11.** A thorough ENT examination can exclude the diagnosis of pOSAs | **-** | **-** | **-** |  |  | |
|  |  | **12.** An apnea/hypopnea index of 0-5 on PSG is normal in children. | **-** | **-** | **-** |  |  | |
|  | 5) Treatment options | **13.** Unlike in adults, CPAP is never useful in pOSAs | **-** | **-** | **-** |  |  | |
|  |  | **14.** Adenotonsillectomy is mandatory in moderate/severe pOSAs | **-** | **-** | **-** |  |  | |
|  | | | **No** | **A little** | **Enough** | **Very** | | **Very much** |
| **Section 2**  **Attitude** | a) Importance of clinical disorder and stratification of anesthetic risk | **1.** Severe POSAS may cause pulmonary hypertension and therefore cardiac evaluation is indicated. | **-** | **-** | **-** | **-** | | **-** |
|  |  | **2.** Children with OSA have a higher anaesthetic risk and should be referred to centers with Pediatric Intensive Care. | **-** | **-** | **-** | **-** | | **-** |
|  | b) Importance of identifying the disorder | **3.** A child with OSA undergoing adenotonsillectomy does not require any particular post-operative precautions | **-** | **-** | **-** | **-** | | **-** |
|  | c) Pharmacological appropriateness | **4.** Opioid analgesics can be safely used at standard dosages in the intraoperative period | **-** | **-** | **-** | **-** | | **-** |
|  |  | **5.** In children with OSA it is preferable not to use inhaled anesthetics | **-** | **-** | **-** | **-** | | **-** |
|  |  | **6.** In children with OSA it is preferable to avoid neuromuscular blockade | **-** | **-** | **-** | **-** | | **-** |
|  | d) Post-operative/post-procedure management | **7.** In case of suspected pOSAs it is advisable to monitor vital signs for 24 hours after a procedure under sedation/AG | **-** | **-** | **-** | **-** | | **-** |
|  |  | **8.** Postoperative analgesia with opioids should be reduced in case of moderate/severe pOSAs | **-** | **-** | **-** | **-** | | **-** |
|  | e) Team Work and Multidisciplinary Management | **9.** It is advisable to set up shared multidisciplinary procedures for the management of children with OSA. | **-** | **-** | **-** | **-** | | **-** |
