# Supplementary material for: Development of a measure of knowledge and attitudes about obstructive sleep apnea for pediatric anesthesia (OSAKA-PedAn) and survey of knowledge and attitudes about pediatric obstructive sleep apnea among Italian anesthesiologists
Source: J Anesth Analg Crit Care. 2025 Jul 1;5:39. doi: 10.1186/s44158-025-00260-z (PMC12219456; doi:10.1186/s44158-025-00260-z)
Supplement: Supplementary file 2 — Supplementary Material 2. Appendix 2. [file 44158_2025_260_MOESM2_ESM.pdf]

Report Statistico

Sindrome delle Apnee Ostruttive del Sonno pediatriche

Elaborato di:

Dr. Matteo Velardo
Dott.ssa Angelica Del Vecchio

RISERVATO

Sommario

Introduzione 1

Statistiche descrittive 2

Genere 2

Età dell'intervistato 2

Status di specialità 2

Regione di appartenenza 3

Tipologia di struttura sanitaria 3

Tipologia ospedale 4

Presenza di HUB o centro di riferimento con T.I. pediatrica nella regione 4

Sedazioni e/o anestesie pediatriche effettuate in un anno 5

Sezione 1 – Conoscenza della pOSAs 5

Valutazione livello di conoscenza della pOSAs 5

Epidemiologia 5

Baseline per questionario: "Sbagliata", Baseline per N. Anestesie annuali: "20-39" 11

Fisiologia 11

Baseline per questionario: "Sbagliata", Baseline per N. Anestesie annuali: "20-39" 15

Baseline per questionario: "Sbagliata", Baseline per N. Anestesie annuali: "20-39" 21

Tematiche di interesse per la formazione 34

Diagnosi 34

Stratificazione del rischio 35

Trattamento e gestione 36

Decorso post-operatorio 37

NIV in pOSAs 38

Sezione 2 – Atteggiamento in pOSAs 39

Importanza del disturbo clinico e stratificazione del rischio anestesiologico 39

1- pOSAs severa può causare ipertensione polmonare è quindi indicata valutazione cardiologia 39

2- I bambini affetti da OSAs presentano un rischio anestesiologico maggiore e vanno indirizzati a centri dotati di Terapia Intensiva Pediatrica 40

Importanza dell'identificazione del disturbo 41

3- Il bambino affetto da OSAs sottoposto ad adenotonsillectomia necessita di particolari accorgimenti postoperatori 41

Appropriatezza farmacologica 42

4- Gli analgesici oppioidi possono essere utilizzati con tranquillità a dosaggio standard nel periodo intraoperatorio 42

5- Nel bambino affetto da OSAs è preferibile non utilizzare anestetici inalatori 43

6- Nei bambini affetti da OSAs è preferibile evitare il blocco neuromuscolare 44

Gestione del post-operatorio/post-proceduta 45

RISERVATO

7- In caso di pOSAs sospetta è opportuno monitorare i parametri vitali per 24h dopo una proceduta in sedazione/AG 45
8- L'analgesia postoperatoria con oppioidi deve prevedere dosaggi ridotti in caso di pOSAs moderata/severa 46
9- Team Work e gestione multidisciplinare 47

RISERVATO

Introduzione

Si presentano in questo report statistico i risultati di una analisi volta ad indagare sulle competenze degli intervistati in merito al pOSAs.
In accordo con l'obiettivo dello studio, si è proceduto ad un'analisi delle risposte aggregandole per "Esperienza" ovvero considerando sia l'anzianità lavorativa, e quindi distinguendo tra Specializzando (Specializzando 1°-3° anno e Specializzando 4°-5° anno) e Specialista (Specialista 31-40, Specialista 41-50, Specialista 51-60, Specialista over 61), che considerando il numero di il numero di sedazioni e/o anestesie pediatriche effettuate in un anno raggruppate nelle seguenti classi: 20-39, 40-149, 150-25, >251. Si considera un ulteriore aggregazione in accordo con la Struttura presso cui l'intervistato dichiara di svolgere prevalentemente la propria attività lavorativa (SP=Struttura pediatrica, SADP=Struttura per adulti con dipartimento pediatrico, SASAP=Struttura per adulti con sporadica attività pediatrica, SA=Struttura esclusivamente per adulti).

Nota metodologica

Per tutte le variabili di interesse si presentano distribuzioni di frequenza assolute e percentuali e relative interpretazioni grafiche.
Per le domande che valutano la conoscenza dell'intervistato in merito all'argomento di studio è stata effettuata un'analisi parametrica per indagare sull'eventuale presenza di una relazione di dipendenza tra la le probabilità di rispondere correttamente e le variabili indipendenti (Esperienza e Struttura)
Con la domanda 11 viene chiesto all'intervistato di dichiarare un numero di sedazioni/anestesie pediatriche effettuate mensilmente. Le modalità di risposta prevedevano classi di ampiezza pari a 5 anestesie definendo un intervallo totale da 0 a oltre 50 sedazioni/anestesie. Tale informazione è stata codificata in annuale a partire dal valore centrale di ogni classe e successivamente raggruppata in cluster come da esigenza di studio (20-39, 40-149, 150-250, >251). Con l'obiettivo di facilitare l'interpretazione del risultato, a seguito della codifica, si è deciso di unire la classe 40-99 con la classe 100-149.

Le domande 13 e 14 del questionario prevedono una modalità di risposta su scala di Likert:

- Per niente
- Poco
- Abbastanza
- Molto
- Moltissimo

- Fortemente in disaccordo
- In disaccordo
- Né d'accordo né in disaccordo
- D'accordo
- Fortemente d'accordo

Per queste domande si propone un indicatore che riassume le informazioni delle distribuzioni di frequenza restituendo un risultato più robusto che permette un confronto più immediato e meno distorto tra diversi livelli di Esperienza (Anzianità lavorativa – Numero di sedazioni/anestesie), tipologie di strutture. Questo strumento ci permette dunque di capire il grado di accordo espresso dagli intervistati verso un giudizio dell'Item che va da "Per niente" a "Moltissimo" per l'Item 13, "Fortemente in disaccordo" a "Fortemente d'accordo" per l'Item 14. Si ha massima concordanza verso il giudizio peggiore quando l'indicatore è uguale a 0, massima concordanza verso il giudizio migliore quando è uguale a 1 e 0,5 esprime il disaccordo tra gli intervistati.

RISERVATO

Statistiche descrittive

Si presentano le statistiche descrittive delle variabili di interesse relative ai soggetti coinvolti nella survey.

Genere

Distribuzione di frequenza assoluta e percentuale del genere. Si osserva che la maggior parte degli intervistati è di sesso femminile.

Genere	Frequenze
Femmina	143 (64,4%)
Maschio	79 (35,6%)

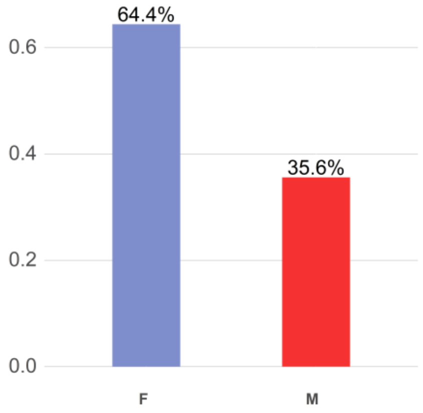

Età dell'intervistato

Si presenta il box-plot relativo all'età degli intervistati. L'età mediana di coloro che hanno partecipato alla survey è di 43 anni. L'età media è pari a 43,5 ±10,8.

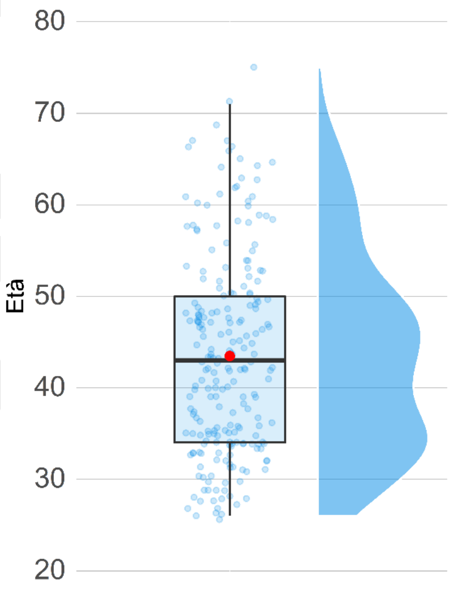

Status di specialità

Distribuzione di frequenza assoluta e percentuale dello status di specialità. La maggior parte degli intervistati risulta essere uno specialista tra i 31 e 50 anni.

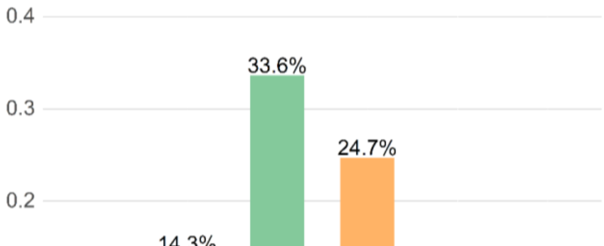

RISERVATO

Esperienza	Frequenze
Specialista over 61	19 (8,5%)
Specialista 51- 60	32 (14,3%)
Specialista 41- 50	75 (33,6%)
Specialista 31- 40	55 (24,7%)
Specializzando 4°-5° anno	18 (8,1%)
Specializzando 1°-3° anno	24 (10,8%)

Regione di appartenenza

Distribuzione di frequenza degli intervistati per Regione in cui dichiarano di svolgere la propria attività lavorativa. Si evince che gli intervistati sembrano distribuirsi omogeneamente sul territorio nazionale.

Regioni	Frequenze
Abruzzo	9 (4%)
Calabria	7 (3,1%)
Campania	8 (3,6%)
Emilia-Romagna	18 (8,1%)
Friuli-Venezia Giulia	6 (2,7%)
Lazio	25 (11,2%)
Liguria	6 (2,7%)
Lombardia	38 (17%)
Marche	13 (5,8%)
Piemonte	17 (7,6%)
Puglia	18 (8,1%)
Sardegna	2 (0,9%)
Sicilia	13 (5,8%)
Toscana	15 (6,7%)
Trentino-Alto Adige	4 (1,8%)
Umbria	7 (3,1%)
Valle d'Aosta	1 (0,4%)
Veneto	16 (7,2%)

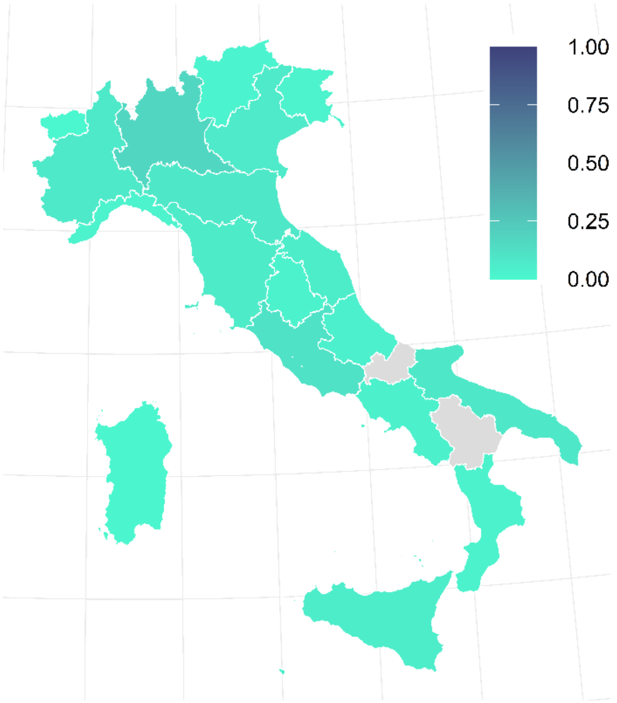

Tipologia di struttura sanitaria

Distribuzione di frequenza assoluta e percentuale della struttura sanitaria presso cui gli intervistati dichiarano di svolgere la propria attività lavorativa. Si nota come la maggior parte degli intervistati dichiara di lavorare presso un'azienda ospedaliera.

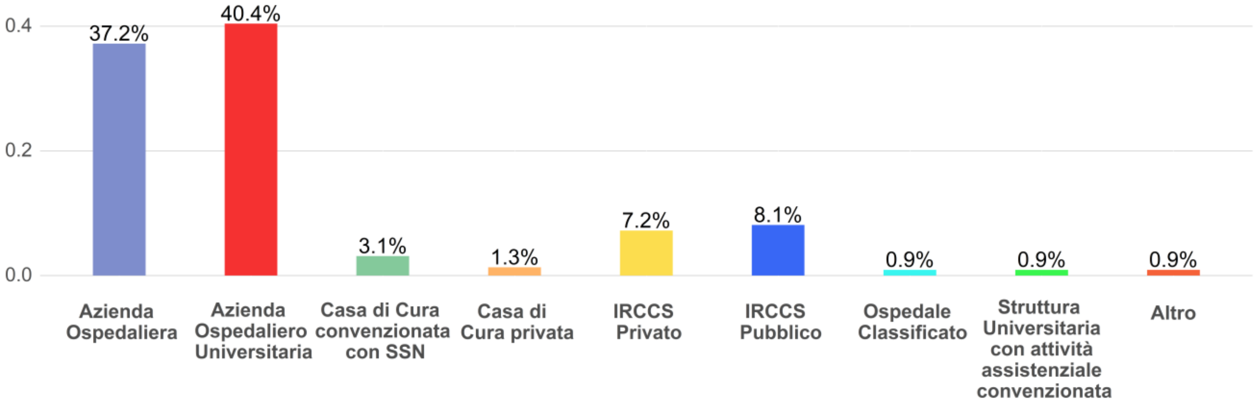

RISERVATO

Struttura	Frequenze
Azienda Ospedaliera	83 (37,2%)
Azienda Ospedaliero-Universitaria	90 (40,4%)
Struttura Universitaria con attività assistenziale convenzionata	2 (0,9%)
Casa di Cura convenzionata con SSN	7 (3,1%)
Casa di Cura privata	3 (1,3%)
IRCCS Privato	16 (7,2%)
IRCCS Pubblico	18 (8,1%)
Ospedale Classificato	2 (0,9%)
Altro	2 (0,9%)

Struttura - Altro	Frequenze
Presidio Ospedaliero della ASL	1 (0,4%)
Ospedale pubblico afferente ad ASL	1 (0,4%)

Tipologia ospedale

Distribuzione di frequenza assoluta e percentuale della tipologia di ospedale presso cui gli intervistati svolgono la propria attività lavorativa. La maggior parte degli intervistati dichiara di lavorare presso una struttura per adulti che svolge attività pediatrica.

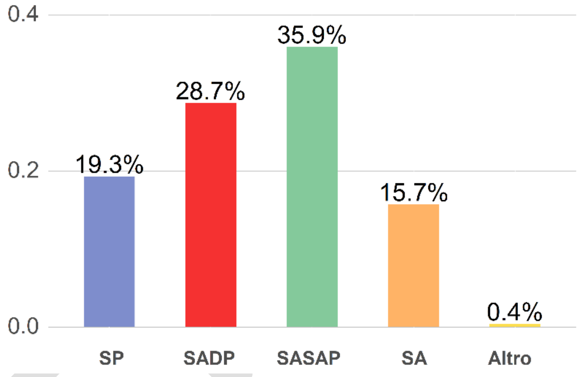

Tipologia Ospedale	Frequenze
Struttura pediatrica	43 (19,3%)
Struttura per adulti con dipartimento pediatrico	64 (28,7%)
Struttura per adulti con sporadica attività pediatrica	80 (35,9%)
Struttura esclusivamente per adulti	35 (15,7%)
Altro	1 (0,4%)

Presenza di HUB o centro di riferimento con T.I. pediatrica nella regione

Si presenta la distribuzione di frequenza assoluta e percentuale della presenza di HUB o centro di riferimento con terapia intensiva pediatrica. Quasi la totalità degli intervistati dichiara che nella propria regione è presente un HUB o un centro di riferimento con Terapia Intensiva pediatrica.

T.I. Pediatrica	Frequenze
Sì	198 (88,8%)
No	19 (8,5%)
Non lo so	6 (2,7%)

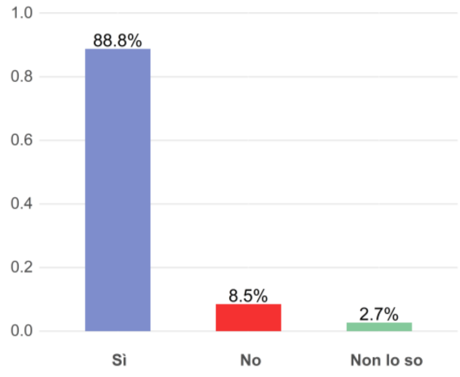

Sedazioni e/o anestesie pediatriche effettuate in un anno

Si presenta la distribuzione di frequenza assoluta e percentuale di sedazioni/anestesie pediatriche effettuate in un anno. Più del 50% degli intervistati dichiara di effettuare tra le 20 e le 39 sedazione/anestesie pediatriche l’anno.

Classi Anestesie Annue	Frequenze
**20-39**	121 (54,3%)
**40-149**	26 (11,7%)
**150-250**	23 (10,3%)
**>251**	53 (23,8%)

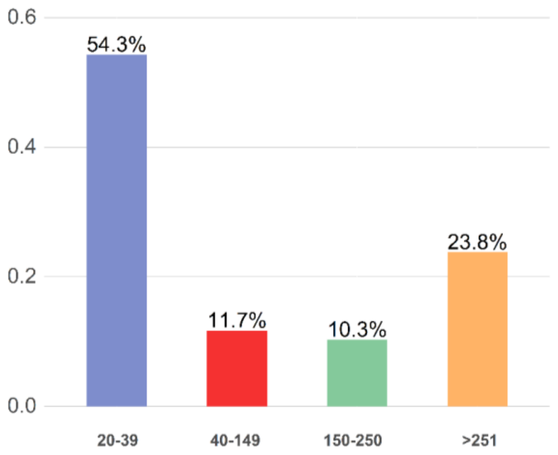

RISERVA

Sezione 1 – Conoscenza della pOSAs

Valutazione livello di conoscenza della pOSAs

Considerando le domande del questionario riferite alla valutazione della conoscenza dell’argomento di interesse pOSAs da parte degli intervistati, si presentano le distribuzioni di frequenza assoluta e percentuale delle risposte individuate come “Corrette” e “Sbagliate”. Non potendo attribuire una valutazione alla risposta “Non lo so”, questa è stata interpretata come volontà da parte dell’intervistato di non rispondere (causa insufficiente conoscenza dell’argomento) e codificata come “Astenuto”. Si presentano inoltre i risultati di una analisi parametrica volta ad indagare come le variabili esplicative “Esperienza”, “Struttura” e “Numero di Anestesie Annue” possano influenzare la probabilità di rispondere correttamente.

Epidemiologia

1. L’incidenza di pOSAs

È stato chiesto agli intervistati se l'incidenza di pOSAs è di 0.1-0.5%, 65 intervistati (29%) ha risposto correttamente, 64 intervistati (29%) ha risposto erroneamente e 94 intervistati (42%) ha dichiarato di non conoscere la risposta. Si presentano in tabella le distribuzioni di frequenza delle risposte Corrette/Sbagliate e degli Astenuti, aggregate in accordo con le variabili di interesse.

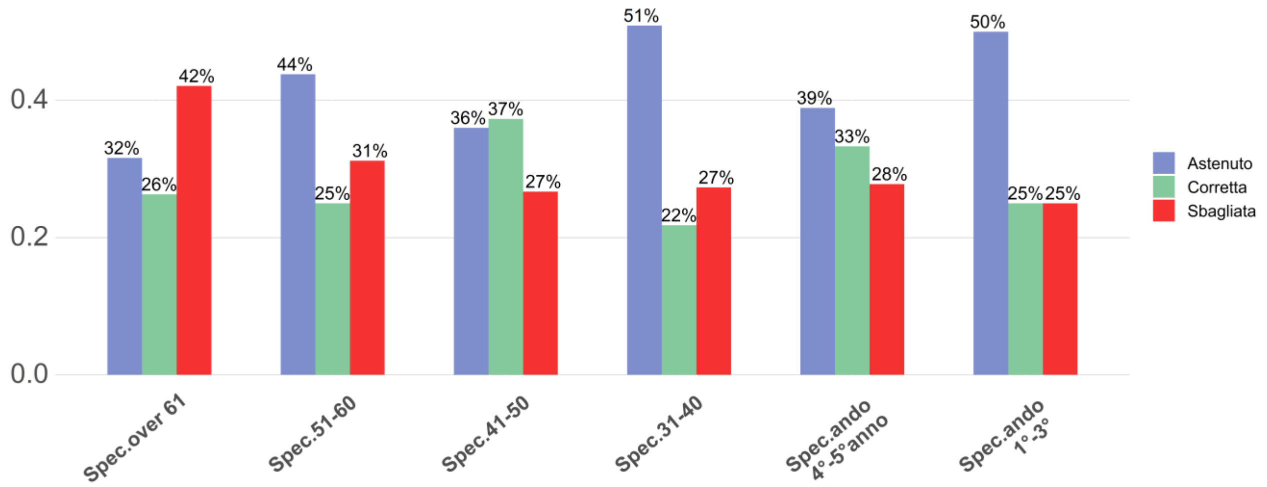

Esperienza	Corrette	Sbagliate	Astenuto
**Specialista over 61**	5 (26,3%)	8 (42,1%)	6 (31,6%)
**Specialista 51-60**	8 (25%)	10 (31,2%)	14 (43,8%)
**Specialista 41-50**	28 (37,3%)	20 (26,7%)	27 (36%)
**Specialista 31-40**	12 (21,8%)	15 (27,3%)	28 (50,9%)
**Specializzando 4°-5° anno**	6 (33,3%)	5 (27,8%)	7 (38,9%)
**Specializzando 1°-3° anno**	6 (25%)	6 (25%)	12 (50%)

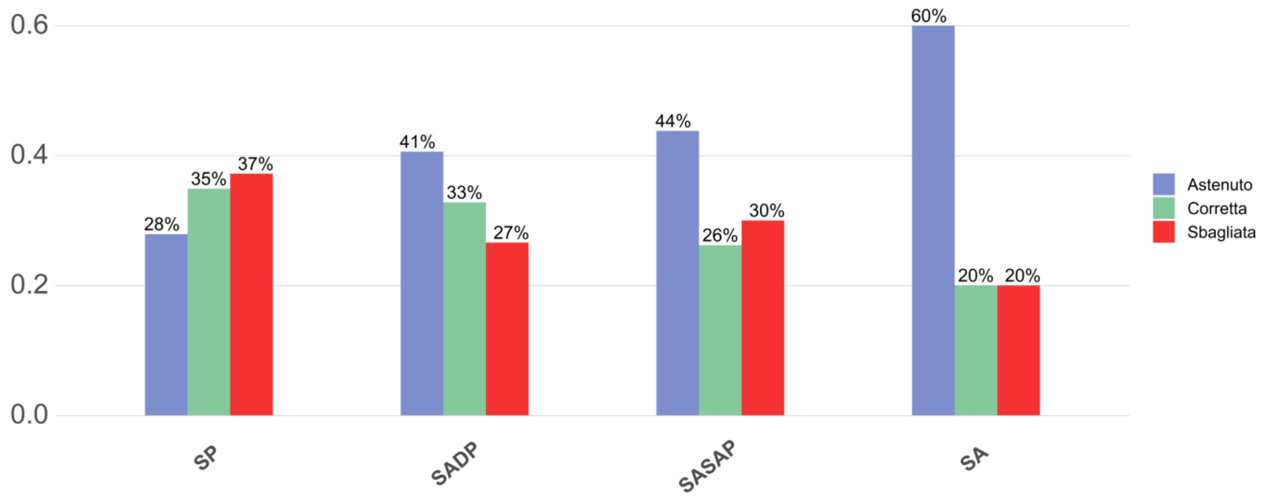

Struttura	Corrette	Sbagliate	Astenuto
**Struttura pediatrica**	15 (34,9%)	16 (37,2%)	12 (27,9%)
**Struttura per adulti con dipartimento pediatrico**	21 (32,8%)	17 (26,6%)	26 (40,6%)
**Struttura per adulti con sporadica attività pediatrica**	21 (26,2%)	24 (30%)	35 (43,8%)
**Struttura esclusivamente per adulti**	7 (20%)	7 (20%)	21 (60%)

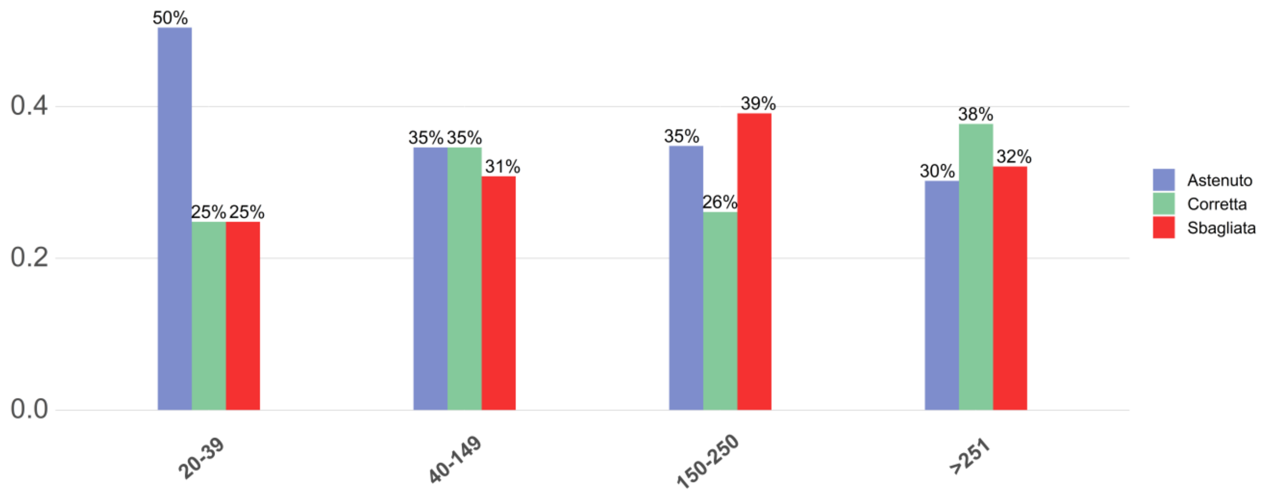

N. Anestesie Annue	Corrette	Sbagliate	Astenuto
**20-39**	30 (24,8%)	30 (24,8%)	61 (50,4%)
**40-149**	9 (34,6%)	8 (30,8%)	9 (34,6%)
**150-250**	6 (26,1%)	9 (39,1%)	8 (34,8%)
**>251**	20 (37,7%)	17 (32,1%)	16 (30,2%)

Osservando le distribuzioni delle risposte rispetto al tipo di struttura in cui esercita l'intervistato e all'esperienza (misurata da anzianità lavorativa e numero di anestesia annualmente praticate), non sembra esservi la presenza di una associazione. I risultati dell'applicazione di un modello di regressione logistica che vede come variabile dipendente la "risposta" (Corretta/Sbagliata), confermano l'assenza di una relazione di dipendenza (p>.05)

2. Disturbi Respiratori del Sonno nei bambini

È stato chiesto agli intervistati se il 10-15% dei bambini presenta Disturbi Respiratori del Sonno, 21 intervistati (9%) ha risposto correttamente, 51 intervistati (23%) ha risposto erroneamente e 151 intervistati (68%) ha dichiarato di non conoscere la risposta. Si presentano in tabella le distribuzioni di frequenza delle risposte Corrette/Sbagliate e degli Astenuti, aggregate in accordo con le variabili di interesse.

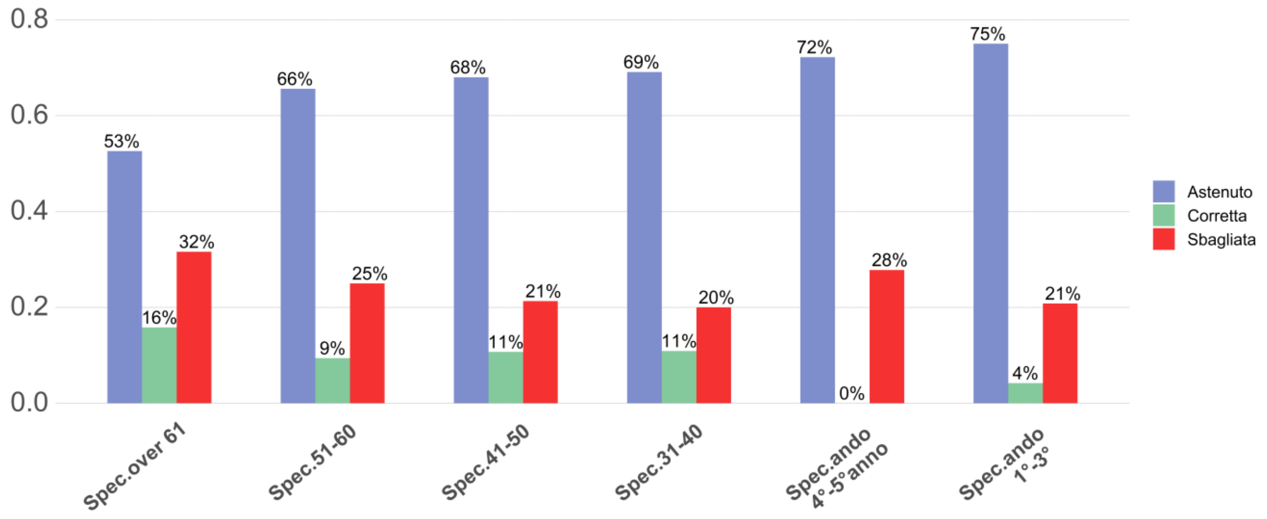

Esperienza	Corrette	Sbagliate	Astenuto

**Specialista over 61**	3 (15,8%)	6 (31,6%)	10 (52,6%)
**Specialista 51-60**	3 (9,4%)	8 (25%)	21 (65,6%)
**Specialista 41-50**	8 (10,7%)	16 (21,3%)	51 (68%)
**Specialista 31-40**	6 (10,9%)	11 (20%)	38 (69,1%)
**Specializzando 4°-5° anno**	0 (0%)	5 (27,8%)	13 (72,2%)
**Specializzando 1°-3° anno**	1 (4,2%)	5 (20,8%)	18 (75%)

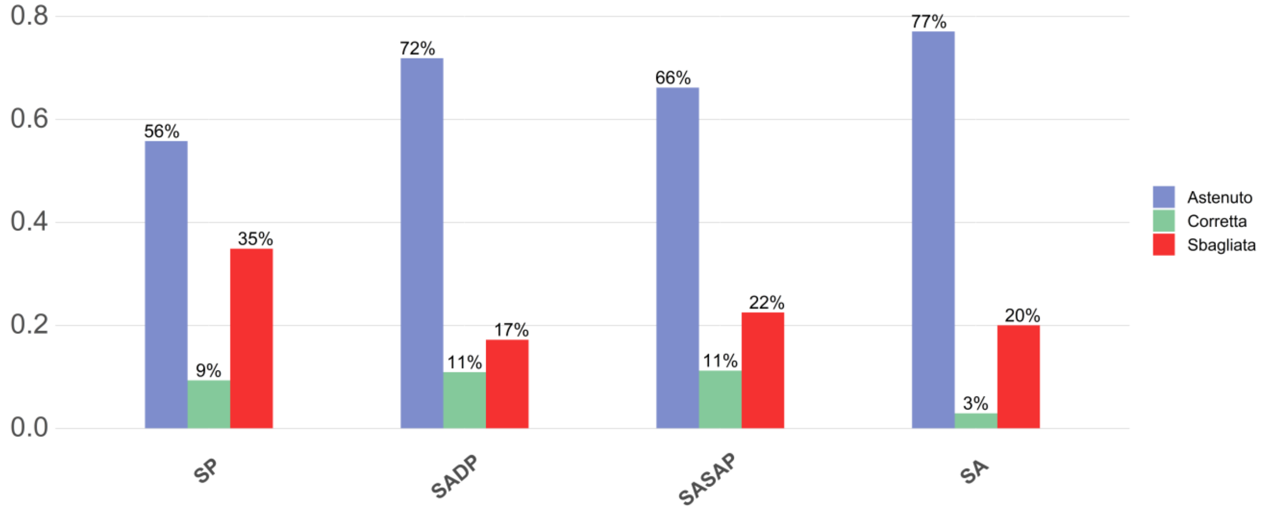

**Struttura**	**Corrette**	**Sbagliate**	**Astenuto**
**Struttura pediatrica**	4 (9,3%)	15 (34,9%)	24 (55,8%)
**Struttura per adulti con dipartimento pediatrico**	7 (10,9%)	11 (17,2%)	46 (71,9%)
**Struttura per adulti con sporadica attività pediatrica**	9 (11,2%)	18 (22,5%)	53 (66,2%)
**Struttura esclusivamente per adulti**	1 (2,9%)	7 (20%)	27 (77,1%)

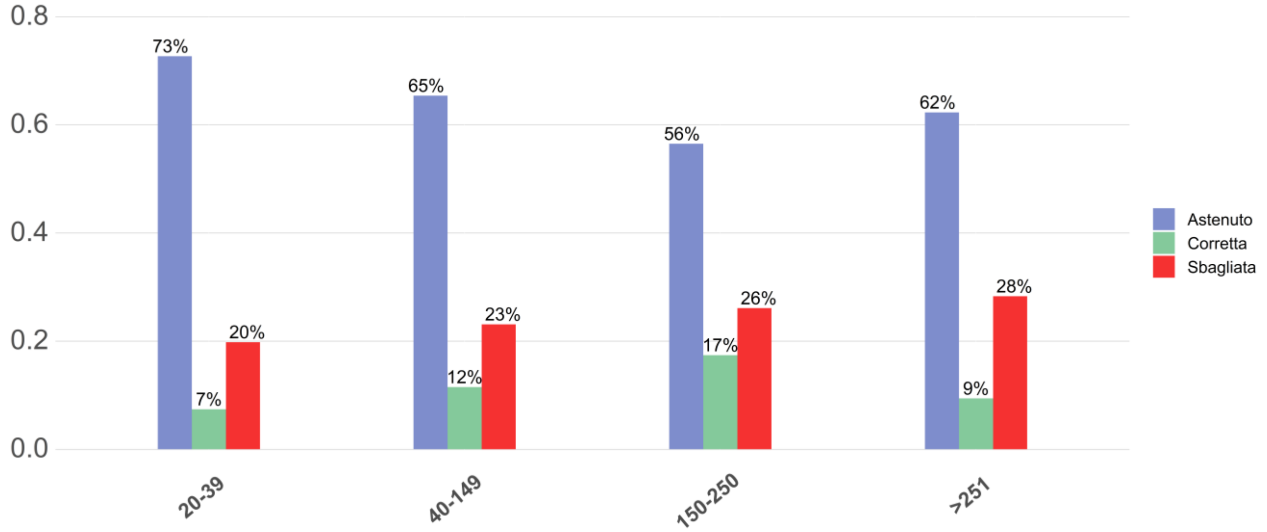

**N. Anestesie Annue**	**Corrette**	**Sbagliate**	**Astenuto**
**20-39**	9 (7,4%)	24 (19,8%)	88 (72,7%)
**40-149**	3 (11,5%)	6 (23,1%)	17 (65,4%)
**150-250**	4 (17,4%)	6 (26,1%)	13 (56,5%)
**>251**	5 (9,4%)	15 (28,3%)	33 (62,3%)

Osservando le distribuzioni delle risposte rispetto al tipo di struttura in cui esercita l'intervistato e all'esperienza (misurata da anzianità lavorativa e numero di anestesia annualmente praticate), non sembra esservi la presenza di una associazione. I risultati dell'applicazione di un modello di regressione logistica che vede come variabile dipendente la "risposta" (Corretta/Sbagliata), confermano l'assenza di una relazione di dipendenza (p>.05)

3. pOSAs nel sesso femminile

È stato chiesto agli intervistati se pOSAs è più diffuso nel sesso femminile, 131 intervistati (60%) ha risposto correttamente, 21 intervistati (9%) ha risposto erroneamente e 68 intervistati (31%) ha dichiarato di non conoscere la risposta. Si presentano in tabella le distribuzioni di frequenza delle risposte Corrette/Sbagliate e degli Astenuti, aggregate in accordo con le variabili di interesse.

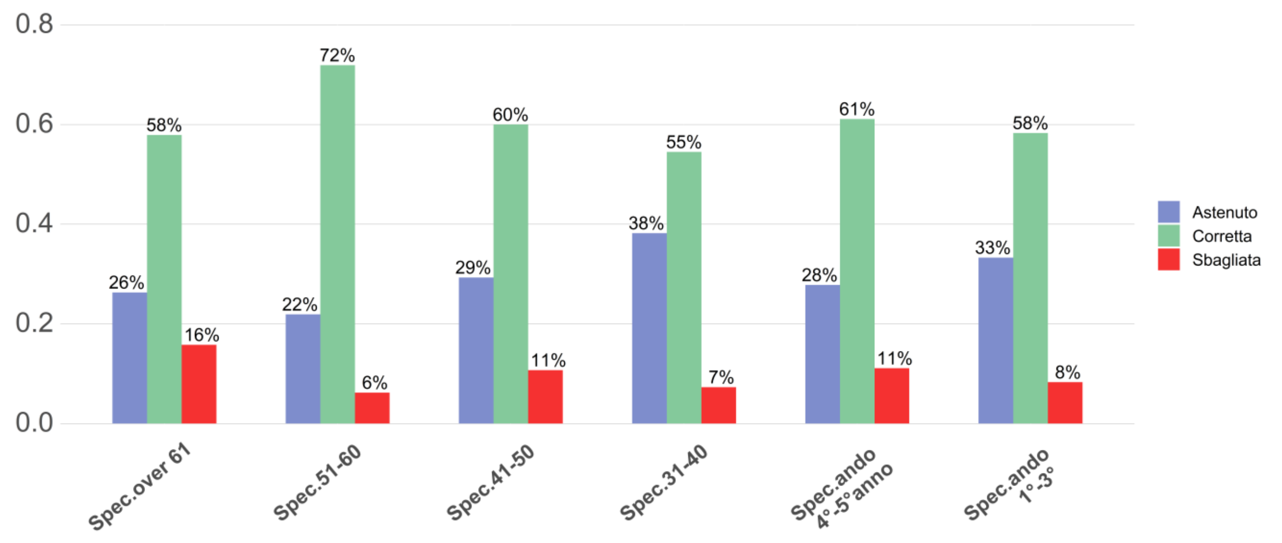

Esperienza	Corrette	Sbagliate	Astenuto
**Specialista over 61**	11 (57,9%)	3 (15,8%)	5 (26,3%)

**Specialista 51-60**	23 (71,9%)	2 (6,2%)	7 (21,9%)
**Specialista 41-50**	45 (60%)	8 (10,7%)	22 (29,3%)
**Specialista 31-40**	30 (54,5%)	4 (7,3%)	21 (38,2%)
**Specializzando 4°-5° anno**	11 (61,1%)	2 (11,1%)	5 (27,8%)
**Specializzando 1°-3° anno**	14 (58,3%)	2 (8,3%)	8 (33,3%)

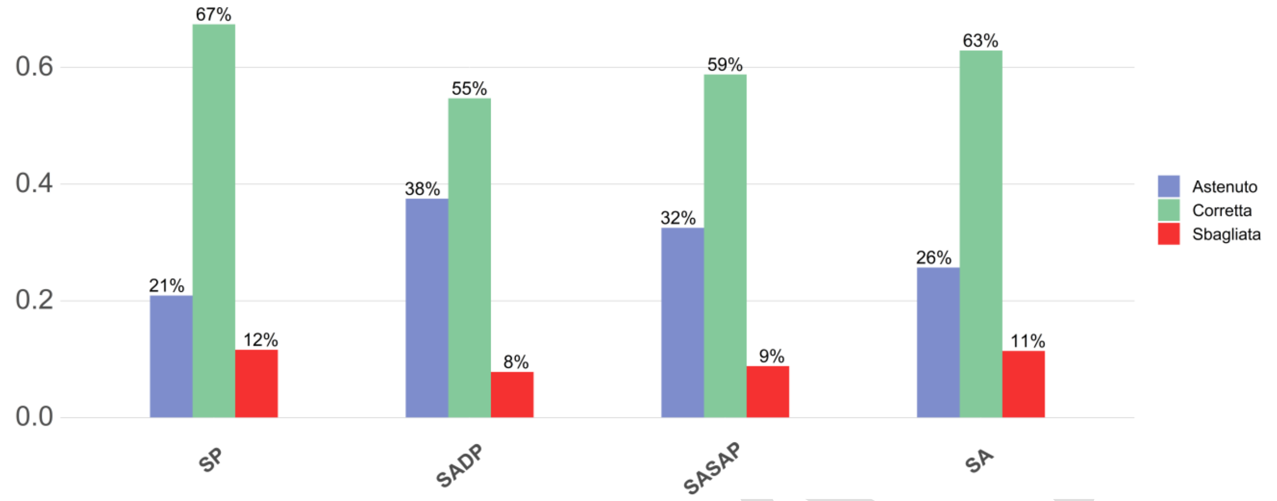

**Struttura**	**Corrette**	**Sbagliate**	**Astenuto**
**Struttura pediatrica**	29 (67,4%)	5 (11,6%)	9 (20,9%)
**Struttura per adulti con dipartimento pediatrico**	35 (54,7%)	5 (7,8%)	24 (37,5%)
**Struttura per adulti con sporadica attività pediatrica**	47 (58,8%)	7 (8,8%)	26 (32,5%)
**Struttura esclusivamente per adulti**	22 (62,9%)	4 (11,4%)	9 (25,7%)

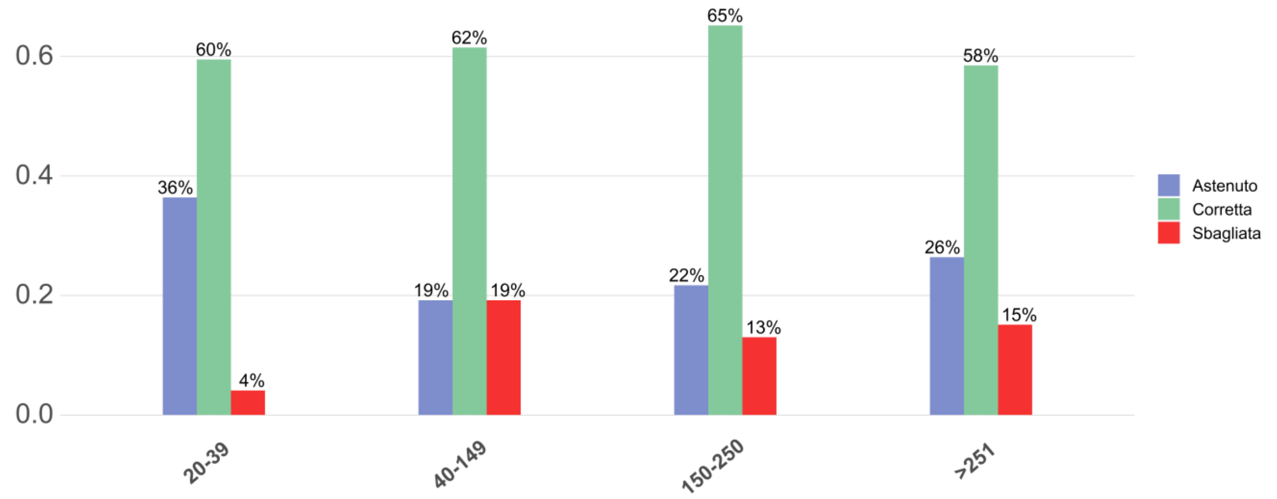

**N. Anestesie Annue**	**Corrette**	**Sbagliate**	**Astenuto**
**20-39**	72 (59,5%)	5 (4,1%)	44 (36,4%)
**40-149**	16 (61,5%)	5 (19,2%)	5 (19,2%)
**150-250**	15 (65,2%)	3 (13%)	5 (21,7%)
**>251**	31 (58,5%)	8 (15,1%)	14 (26,4%)

Osservando le distribuzioni delle risposte rispetto al tipo di struttura in cui esercita l'intervistato e all'esperienza (misurata da anzianità lavorativa), non sembra esservi la presenza di una associazione. I risultati dell'applicazione di un modello di regressione logistica che vede come variabile dipendente la "risposta" (Corretta/Sbagliata), confermano l'assenza di una relazione di dipendenza (p>.05)

Osservando le distribuzioni delle risposte rispetto al numero di anestesia annualmente praticate annualmente, vi è la presenza di una associazione. Dai risultati dell'applicazione di un modello di regressione logistica che vede come variabile dipendente la "risposta" (Corretta/Sbagliata), si evince che è presente una relazione significativa di dipendenza. La quota degli anestesisti che dichiara di effettuare più di 251 anestesie in un anno che hanno risposto correttamente al questionario è 0,27 volte inferiore la quota degli anestesisti che dichiarano di eseguire 20-39 anestesie annue che hanno risposto correttamente alla domanda. La probabilità di rispondere correttamente alle domande è dunque il 27% minore per chi esegue più di 251 anestesie l'anno rispetto a chi ne esegue 20-39 l'anno.

Risposta Test	Numero Anestesie annuali	β	p-value	OR
Corretta	**40 - 149**	-1,5041	0,031	0,222
	**150 – 250**	-1,0578	0,177	0,347
	**>251**	-1,3127	**0,03**	0,270

Baseline per questionario: "Sbagliata", Baseline per N. Anestesie annuali: "20-39"

Fisiologia

4. Causa di OSAs

È stato chiesto agli intervistati se le malformazioni craniofacciali possono essere causa di OSAs, 213 intervistati (96%) ha risposto correttamente, nessuno ha risposto erroneamente e 10 intervistati (4%) ha dichiarato di non conoscere la risposta. Si presentano in tabella le distribuzioni di frequenza delle risposte Corrette/Sbagliate e degli Astenuti, aggregate in accordo con le variabili di interesse.

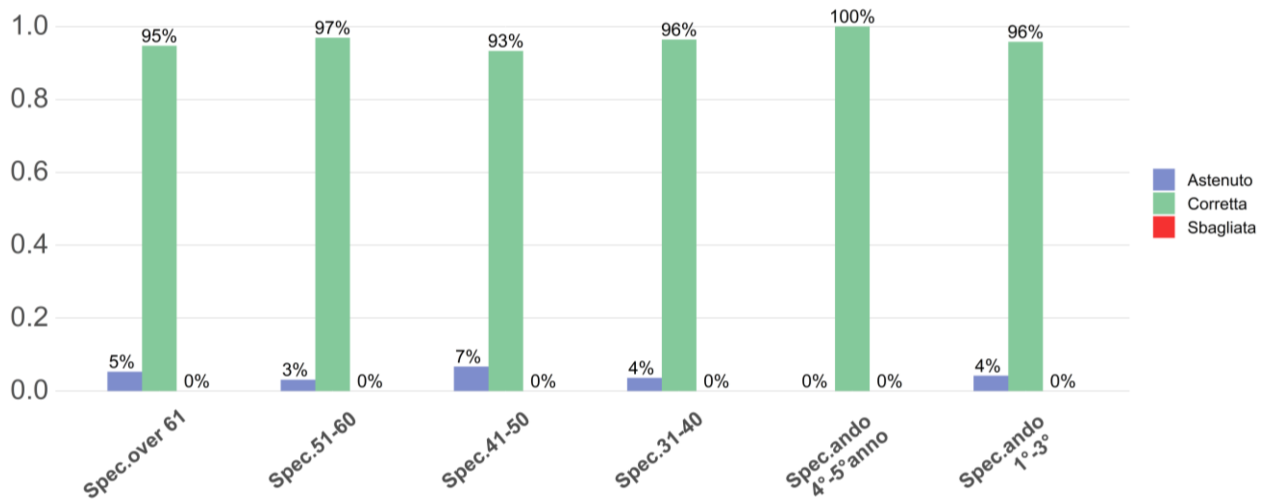

Esperienza	Corrette	Sbagliate	Astenuto
**Specialista over 61**	18 (94,7%)	0 (0%)	1 (5,3%)

**Specialista 51-60**	31 (96,9%)	0 (0%)	1 (3,1%)
**Specialista 41-50**	70 (93,3%)	0 (0%)	5 (6,7%)
**Specialista 31-40**	53 (96,4%)	0 (0%)	2 (3,6%)
**Specializzando 4°-5° anno**	18 (100%)	0 (0%)	0 (0%)
**Specializzando 1°-3° anno**	23 (95,8%)	0 (0%)	1 (4,2%)

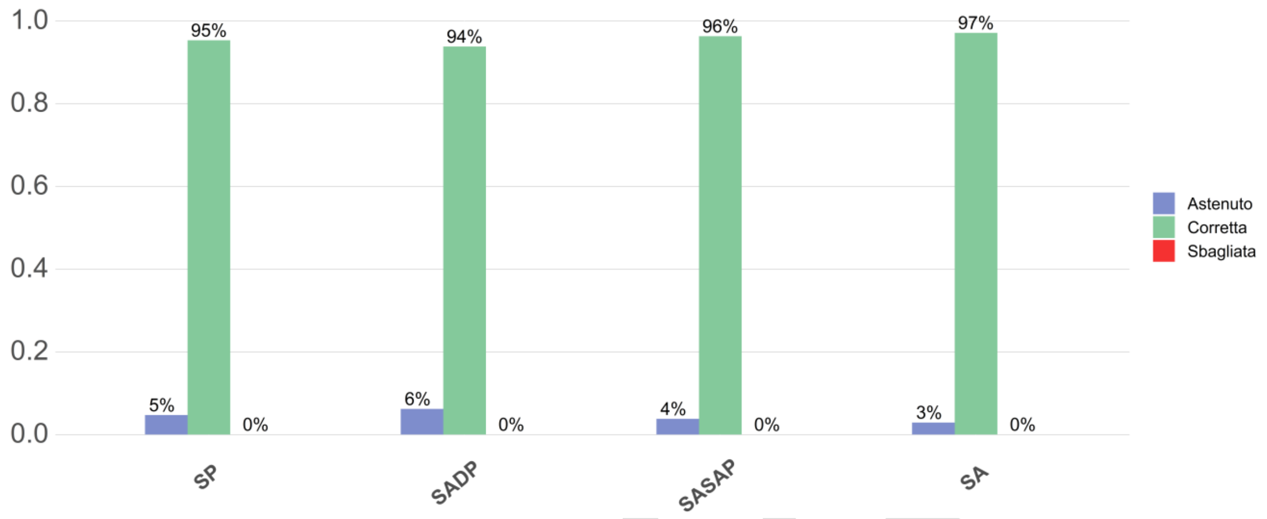

**Struttura**	**Corrette**	**Sbagliate**	**Astenuto**
**Struttura pediatrica**	41 (0%)	0.047 (0%)	2 (95,3%)
**Struttura per adulti con dipartimento pediatrico**	60 (0%)	0.062 (0%)	4 (93,8%)
**Struttura per adulti con sporadica attività pediatrica**	77 (0%)	0.038 (0%)	3 (96,3%)
**Struttura esclusivamente per adulti**	34 (0%)	0.029 (0%)	1 (97,1%)

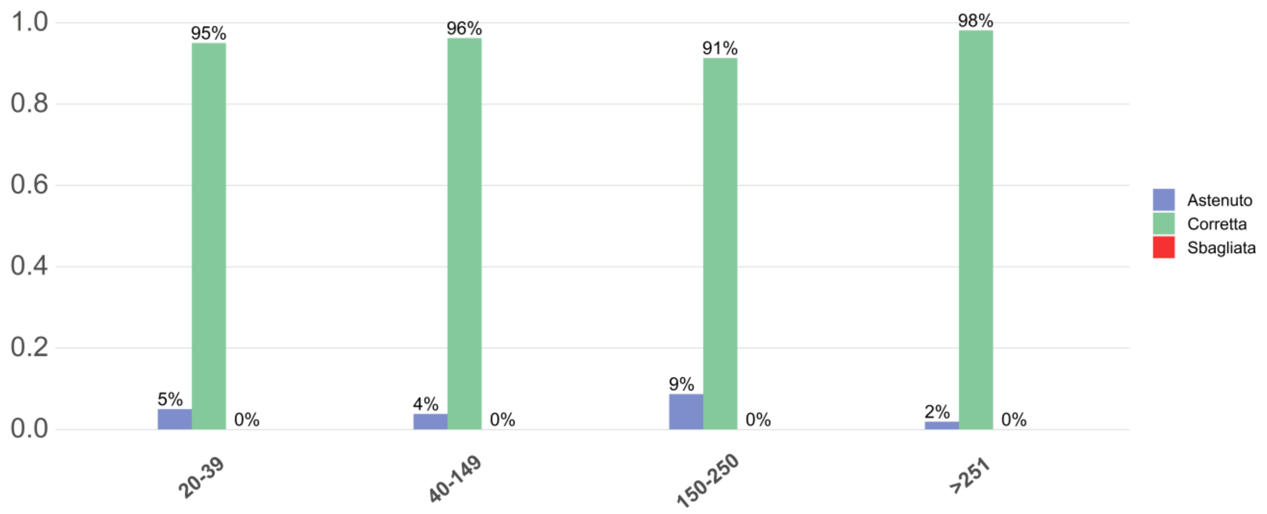

**N. Anestesie Annue**	**Corrette**	**Sbagliate**	**Astenuti**
**20-39**	115 (95%)	0 (0%)	6 (5%)
**40-149**	25 (96,2%)	0 (0%)	1 (3,8%)
**150-250**	21 (91,3%)	0 (0%)	2 (8,7%)
**>251**	52 (98,1%)	0 (0%)	1 (1,9%)

Osservando le distribuzioni delle risposte rispetto al tipo di struttura in cui esercita l'intervistato e all'esperienza (misurata da anzianità lavorativa e numero di anestesia annualmente praticate), non sembra esservi la presenza di una associazione. I risultati dell'applicazione di un modello di regressione logistica che

vede come variabile dipendente la “risposta” (Corretta/Sbagliata), confermano l’assenza di una relazione di dipendenza (p>.05)

5. La causa OSAs nella fascia di età>8aa

È stato chiesto agli intervistati se la causa più frequente di OSAs nella fascia di età>8aa è l’ipertrofia adenotonsillare, 39 intervistati (18%) ha risposto correttamente, 50 intervistati (22%) ha risposto erroneamente e 134 intervistati (60%) ha dichiarato di non conoscere la risposta. Si presentano in tabella le distribuzioni di frequenza delle risposte Corrette/Sbagliate e degli Astenuti, aggregate in accordo con le variabili di interesse.

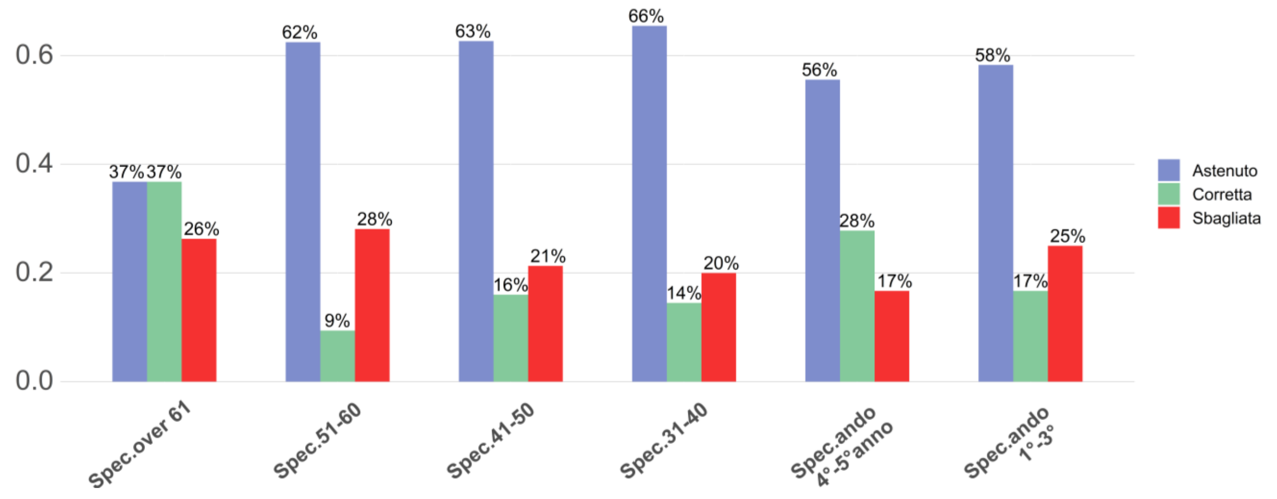

Esperienza	Corrette	Sbagliate	Astenuto
**Specialista over 61**	7 (36,8%)	5 (26,3%)	7 (36,8%)
**Specialista 51-60**	3 (9,4%)	9 (28,1%)	20 (62,5%)
**Specialista 41-50**	12 (16%)	16 (21,3%)	47 (62,7%)
**Specialista 31-40**	8 (14,5%)	11 (20%)	36 (65,5%)
**Specializzando 4°-5° anno**	5 (27,8%)	3 (16,7%)	10 (55,6%)

**Specializzando 1°-3° anno**	4 (16,7%)	6 (25%)	14 (58,3%)

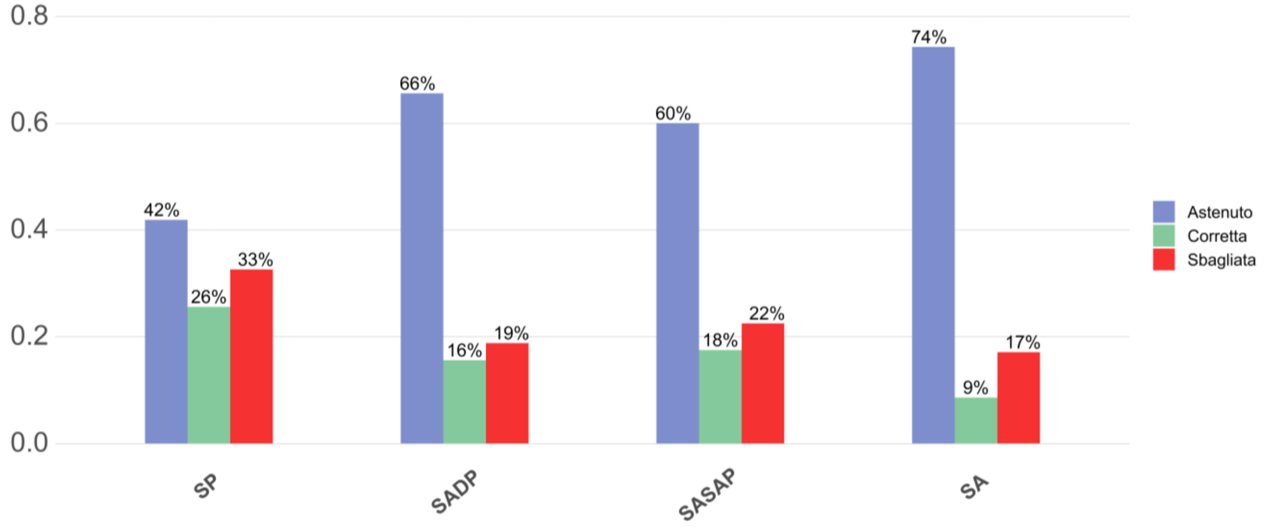

Struttura	Corrette	Sbagliate	Astenuto
**Struttura pediatrica**	11 (25,6%)	14 (32,6%)	18 (41,9%)
**Struttura per adulti con dipartimento pediatrico**	10 (15,6%)	12 (18,8%)	42 (65,6%)
**Struttura per adulti con sporadica attività pediatrica**	14 (17,5%)	18 (22,5%)	48 (60%)
**Struttura esclusivamente per adulti**	3 (8,6%)	6 (17,1%)	26 (74,3%)

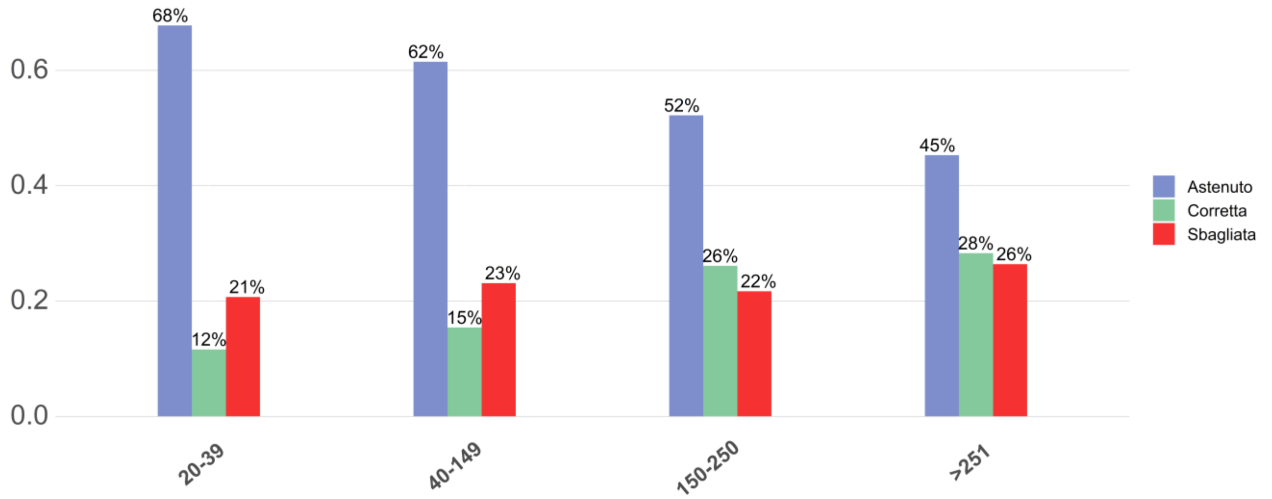

N. Anestesie Annue	Corrette	Sbagliate	Astenuto
**20-39**	14 (11,6%)	25 (20,7%)	82 (67,8%)
**40-149**	4 (15,4%)	6 (23,1%)	16 (61,5%)
**150-250**	6 (26,1%)	5 (21,7%)	12 (52,2%)
**>251**	15 (28,3%)	14 (26,4%)	24 (45,3%)

Osservando le distribuzioni delle risposte rispetto al tipo di struttura in cui esercita l'intervistato e all'esperienza (misurata da anzianità lavorativa), non sembra esservi la presenza di una associazione. I risultati dell'applicazione di un modello di regressione logistica che vede come variabile dipendente la "risposta" (Corretta/Sbagliata), confermano l'assenza di una relazione di dipendenza (p>.05)

Osservando le distribuzioni delle risposte rispetto al numero di anestesia annualmente praticate annualmente, non sembra esservi la presenza di una associazione. Dai risultati dell'applicazione di un modello di regressione logistica che vede come variabile dipendente la "risposta" (Corretta/Sbagliata), si evince che è presente una relazione significativa di dipendenza. La quota degli anestesisti che dichiara di effettuare più di 251 anestesie in un anno che hanno risposto correttamente al questionario è 1,30 volte la quota degli anestesisti che dichiarano di eseguire 20-39 anestesie annue che hanno risposto correttamente alla domanda. La probabilità di rispondere correttamente alle domande è dunque il 30% maggiore per chi esegue più di 251 anestesie l'anno rispetto a chi ne esegue 20-39 l'anno.

Risposta Test	Numero Anestesie annuali	β	p-value	OR
Corretta	**40 - 149**	0,2614	0,670	2,976
	**150 – 250**	0,9853	0,077	2,679
	**>251**	-1,0906	0,009	1,299

Baseline per questionario: "Sbagliata", Baseline per N. Anestesie annuali: "20-39"

6. L'ipotonia muscolare delle vie aeree e pOSAs

È stato chiesto agli intervistati se l'ipotonia muscolare delle vie aeree superiori durante il sonno contribuisce alla pOSAs, 202 intervistati (91%) ha risposto correttamente, 7 intervistati (3%) ha risposto erroneamente e 14 intervistati (6%) ha dichiarato di non conoscere la risposta. Si presentano in tabella le distribuzioni di frequenza delle risposte Corrette/Sbagliate e degli Astenuti, aggregate in accordo con le variabili di interesse.

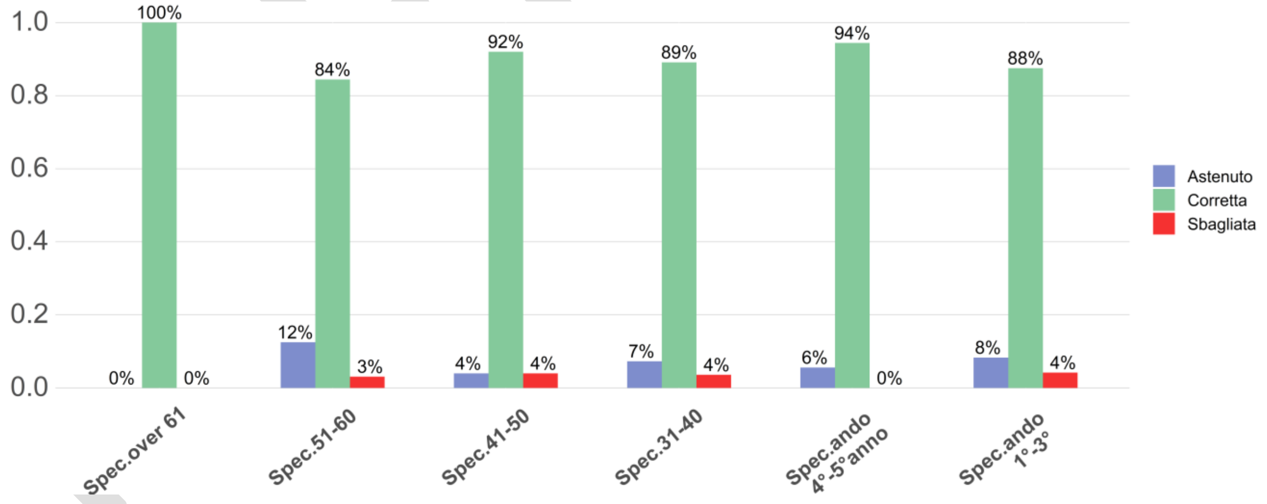

Esperienza	Corrette	Sbagliate	Astenuto
**Specialista over 61**	19 (100%)	0 (0%)	0 (0%)
**Specialista 51-60**	27 (84,4%)	1 (3,1%)	4 (12,5%)
**Specialista 41-50**	69 (92%)	3 (4%)	3 (4%)
**Specialista 31-40**	49 (89,1%)	2 (3,6%)	4 (7,3%)
**Specializzando 4°-5° anno**	17 (94,4%)	0 (0%)	1 (5,6%)
**Specializzando 1°-3° anno**	21 (87,5%)	1 (4,2%)	2 (8,3%)

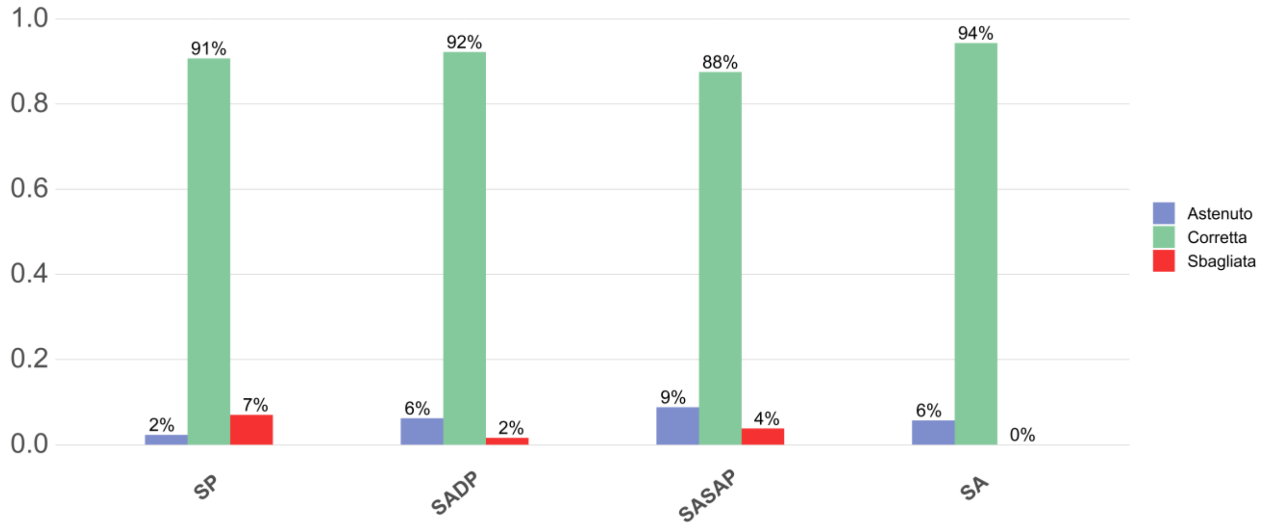

Struttura	Corrette	Sbagliate	Astenuto
**Struttura pediatrica**	39 (90,7%)	3 (7%)	1 (2,3%)
**Struttura per adulti con dipartimento pediatrico**	59 (92,2%)	1 (1,6%)	4 (6,2%)
**Struttura per adulti con sporadica attività pediatrica**	70 (87,5%)	3 (3,8%)	7 (8,8%)
**Struttura esclusivamente per adulti**	33 (94,3%)	0 (0%)	2 (5,7%)

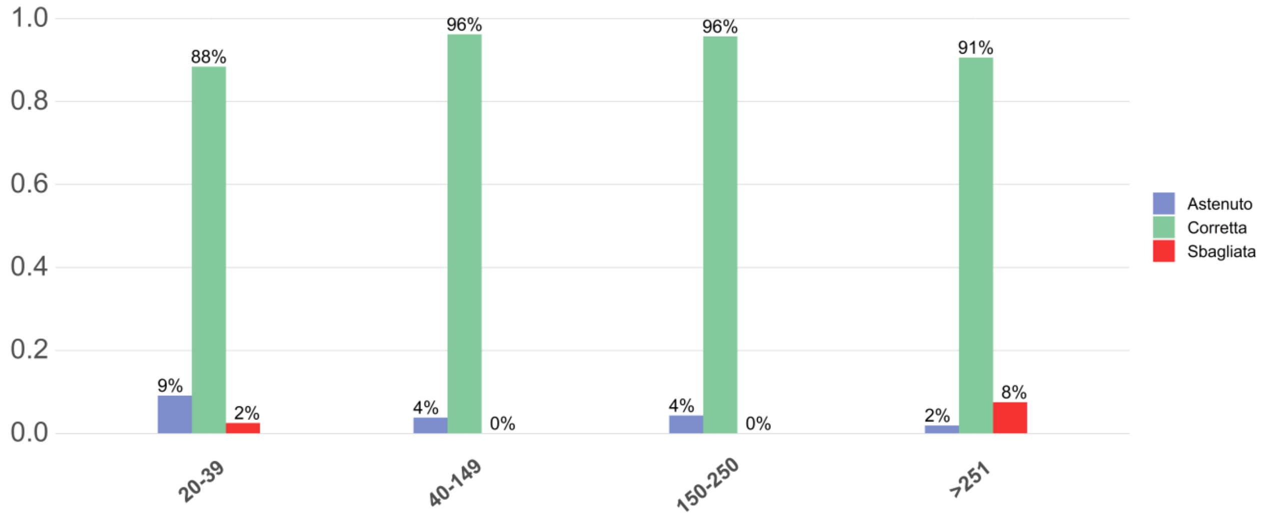

N. Anestesie Annue	Corrette	Sbagliate	Astenuto
**20-39**	107 (88,4%)	3 (2,5%)	11 (9,1%)
**40-149**	25 (96,2%)	0 (0%)	1 (3,8%)
**150-250**	22 (95,7%)	0 (0%)	1 (4,3%)
**>251**	48 (90,6%)	4 (7,5%)	1 (1,9%)

Osservando le distribuzioni delle risposte rispetto al tipo di struttura in cui esercita l'intervistato e all'esperienza (misurata da anzianità lavorativa e numero di anestesia annualmente praticate), non sembra esservi la presenza di una associazione. I risultati dell'applicazione di un modello di regressione logistica che vede come variabile dipendente la "risposta" (Corretta/Sbagliata), confermano l'assenza di una relazione di dipendenza ($p>.05$)

Sintomi

7. Sintomi di pOSAs

È stato chiesto agli intervistati se enuresi può essere sintomo di pOSAs, 141 intervistati (63%) ha risposto correttamente, 16 intervistati (7%) ha risposto erroneamente e 66 intervistati (30%) ha dichiarato di non conoscere la risposta. Si presentano in tabella le distribuzioni di frequenza delle risposte Corrette/Sbagliate e degli Astenuti, aggregate in accordo con le variabili di interesse.

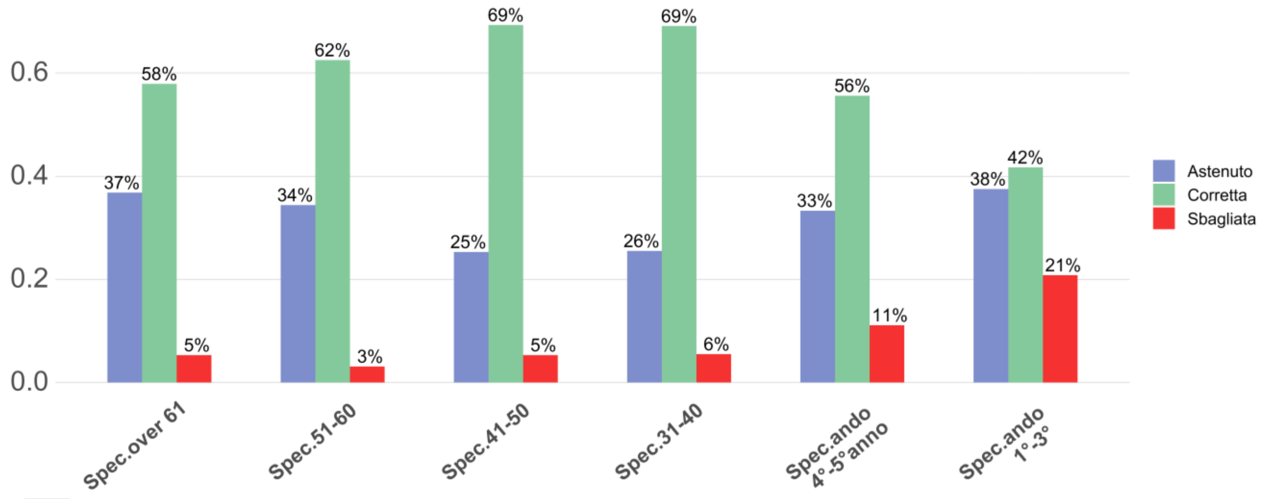

Esperienza	Corrette	Sbagliate	Astenuto
Specialista over 61	11 (57,9%)	1 (5,3%)	7 (36,8%)
Specialista 51-60	20 (62,5%)	1 (3,1%)	11 (34,4%)
Specialista 41-50	52 (69,3%)	4 (5,3%)	19 (25,3%)
Specialista 31-40	38 (69,1%)	3 (5,5%)	14 (25,5%)
Specializzando 4°-5° anno	10 (55,6%)	2 (11,1%)	6 (33,3%)
Specializzando 1°-3° anno	10 (41,7%)	5 (20,8%)	9 (37,5%)

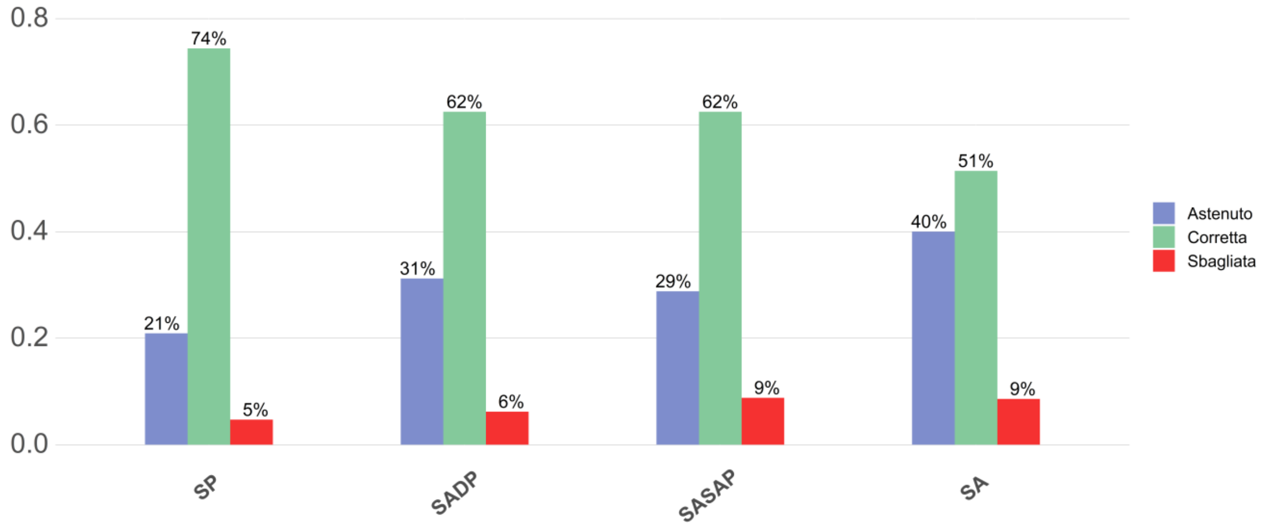

Struttura	Corrette	Sbagliate	Astenuto
Struttura pediatrica	32 (74,4%)	2 (4,7%)	9 (20,9%)
Struttura per adulti con dipartimento pediatrico	40 (62,5%)	4 (6,2%)	20 (31,2%)
Struttura per adulti con sporadica attività pediatrica	50 (62,5%)	7 (8,8%)	23 (28,8%)
Struttura esclusivamente per adulti	18 (51,4%)	3 (8,6%)	14 (40%)

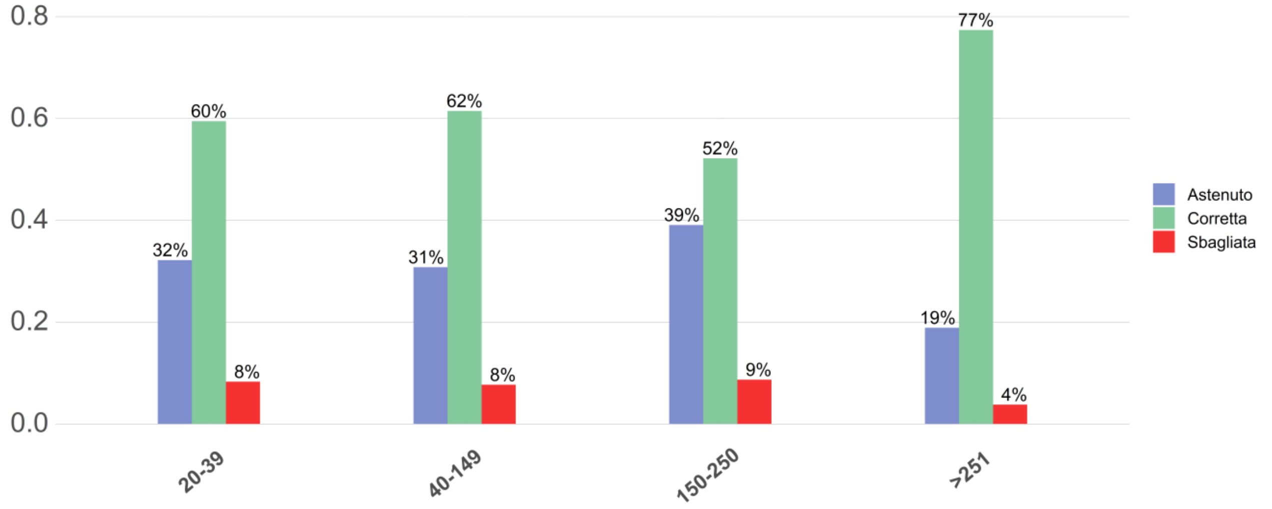

N. Anestesie Annue	Corrette	Sbagliate	Astenuto
20-39	72 (59,5%)	10 (8,3%)	39 (32,2%)
40-149	16 (61,5%)	2 (7,7%)	8 (30,8%)
150-250	12 (52,2%)	2 (8,7%)	9 (39,1%)
>251	41 (77,4%)	2 (3,8%)	10 (18,9%)

Osservando le distribuzioni delle risposte rispetto al tipo di struttura in cui esercita l’intervistato e all’esperienza (misurata da anzianità lavorativa e numero di anestesia annualmente praticate), non sembra esservi la presenza di una associazione. I risultati dell’applicazione di un modello di regressione logistica che vede come variabile dipendente la “risposta” (Corretta/Sbagliata), confermano l’assenza di una relazione di dipendenza (p>.05)

8. OSAs pediatrica ed iperattività

È stato chiesto agli intervistati se il bambino affetto da OSAs può essere iperattivo, 119 intervistati (53%) ha risposto correttamente, 24 intervistati (11%) ha risposto erroneamente e 80 intervistati (36%) ha dichiarato di non conoscere la risposta. Si presentano in tabella le distribuzioni di frequenza delle risposte Corrette/Sbagliate e degli Astenuti, aggregate in accordo con le variabili di interesse.

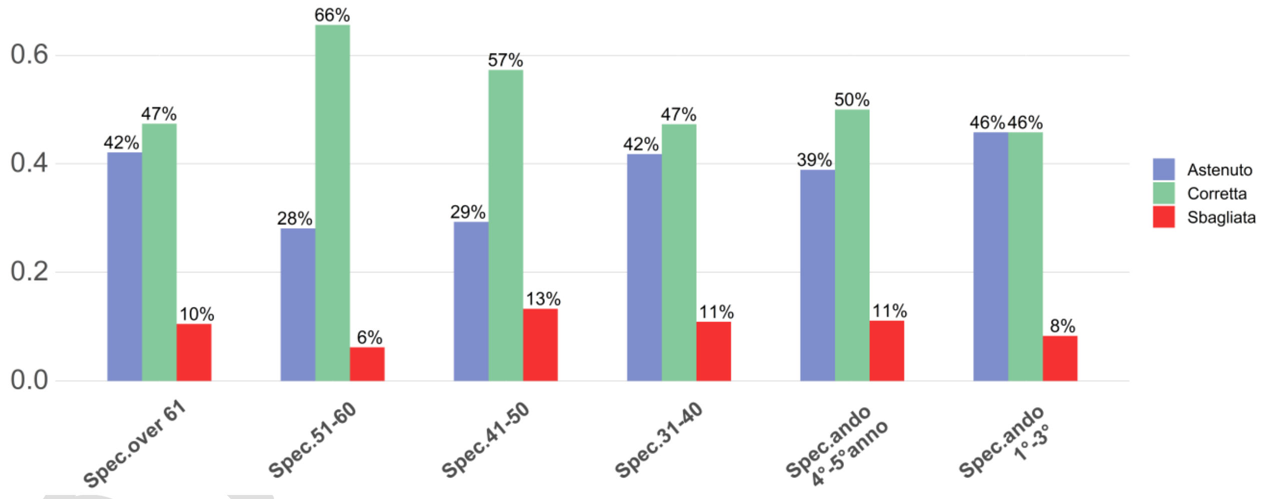

Esperienza	Corrette	Sbagliate	Astenuto
**Specialista over 61**	9 (47,4%)	2 (10,5%)	8 (42,1%)
**Specialista 51-60**	21 (65,6%)	2 (6,2%)	9 (28,1%)
**Specialista 41-50**	43 (57,3%)	10 (13,3%)	22 (29,3%)
**Specialista 31-40**	26 (47,3%)	6 (10,9%)	23 (41,8%)
**Specializzando 4°-5° anno**	9 (50%)	2 (11,1%)	7 (38,9%)
**Specializzando 1°-3° anno**	11 (45,8%)	2 (8,3%)	11 (45,8%)

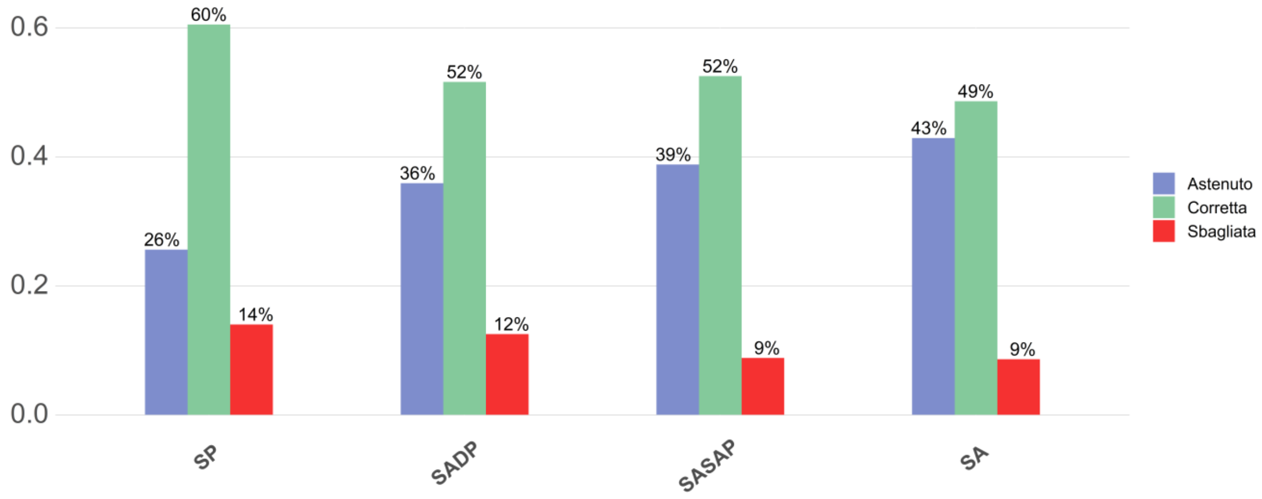

Struttura	Corrette	Sbagliate	Astenuto
Struttura pediatrica	26 (60,5%)	6 (14%)	11 (25,6%)
Struttura per adulti con dipartimento pediatrico	33 (51,6%)	8 (12,5%)	23 (35,9%)
Struttura per adulti con sporadica attività pediatrica	42 (52,5%)	7 (8,8%)	31 (38,8%)
Struttura esclusivamente per adulti	17 (48,6%)	3 (8,6%)	15 (42,9%)

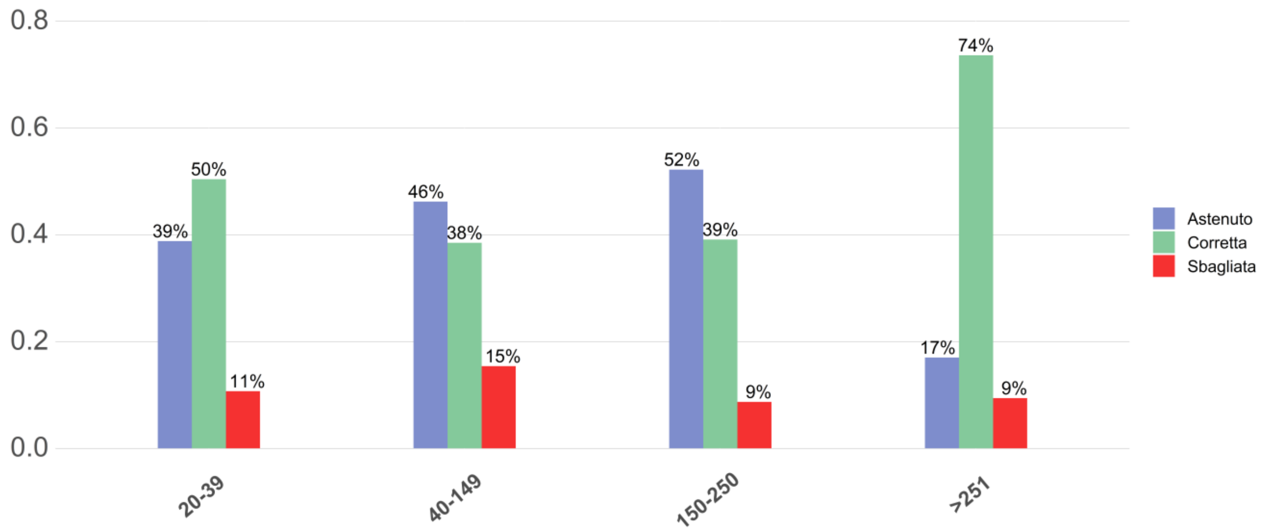

N. Anestesie Annue	Corrette	Sbagliate	Astenuto
20-39	61 (50,4%)	13 (10,7%)	47 (38,8%)
40-149	10 (38,5%)	4 (15,4%)	12 (46,2%)
150-250	9 (39,1%)	2 (8,7%)	12 (52,2%)
>251	39 (73,6%)	5 (9,4%)	9 (17%)

Osservando le distribuzioni delle risposte rispetto al tipo di struttura in cui esercita l'intervistato e all'esperienza (misurata da anzianità lavorativa), non sembra esservi la presenza di una associazione. I risultati dell'applicazione di un modello di regressione logistica che vede come variabile dipendente la "risposta" (Corretta/Sbagliata), confermano l'assenza di una relazione di dipendenza ($p>.05$)

Osservando le distribuzioni delle risposte rispetto al numero di anestesia annualmente praticate annualmente, non sembra esservi la presenza di una associazione. Dai risultati dell'applicazione di un modello di regressione logistica che vede come variabile dipendente la "risposta" (Corretta/Sbagliata), si evince che è presente una relazione significativa di dipendenza. La quota degli anestesisti che dichiara di effettuare più di 251 anestesie in un anno che hanno risposto correttamente al questionario è 2,4 volte la quota degli anestesisti che dichiarano di eseguire 20-39 anestesie annue che hanno risposto correttamente alla domanda. La probabilità di rispondere correttamente alle domande è dunque il 14% maggiore per chi esegue più di 251 anestesie l'anno rispetto a chi ne esegue 20-39 l'anno.

Risposta Test	Numero Anestesie annuali	β	p-value	OR
Corretta	**40 - 149**	-0,569	0,239	0,566
	**150 – 250**	-0,473	0,358	0,623
	**>251**	0,888	**0,031**	2,430

Baseline per questionario: "Sbagliata", Baseline per N. Anestesie annuali: "20-39"

9. Acidosi respiratoria compensata indicatore di pOSAs

È stato chiesto agli intervistati se acidosi respiratoria compensata può suggerire pOSAs, 175 intervistati (79%) ha risposto correttamente, 11 intervistati (5%) ha risposto erroneamente e 37 intervistati (17%) ha dichiarato di non conoscere la risposta. Si presentano in tabella le distribuzioni di frequenza delle risposte Corrette/Sbagliate e degli Astenuti, aggregate in accordo con le variabili di interesse.

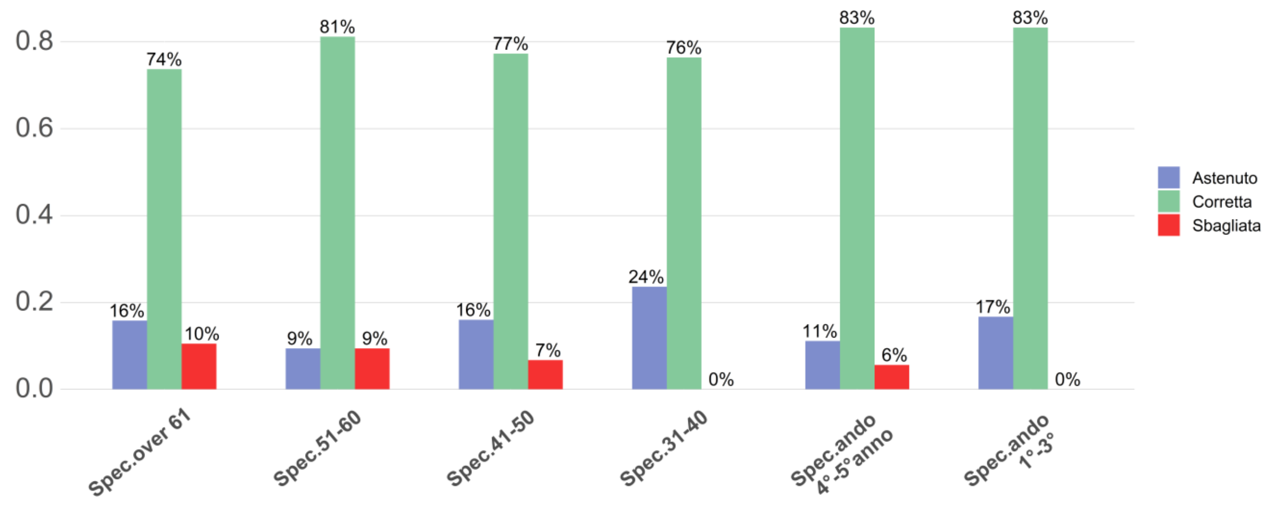

Esperienza	Corrette	Sbagliate	Astenuto
**Specialista over 61**	14 (73,7%)	2 (10,5%)	3 (15,8%)
**Specialista 51-60**	26 (81,2%)	3 (9,4%)	3 (9,4%)
**Specialista 41-50**	58 (77,3%)	5 (6,7%)	12 (16%)
**Specialista 31-40**	42 (76,4%)	0 (0%)	13 (23,6%)
**Specializzando 4°-5° anno**	15 (83,3%)	1 (5,6%)	2 (11,1%)

**Specializzando 1°-3° anno**	20 (83,3%)	0 (0%)	4 (16,7%)

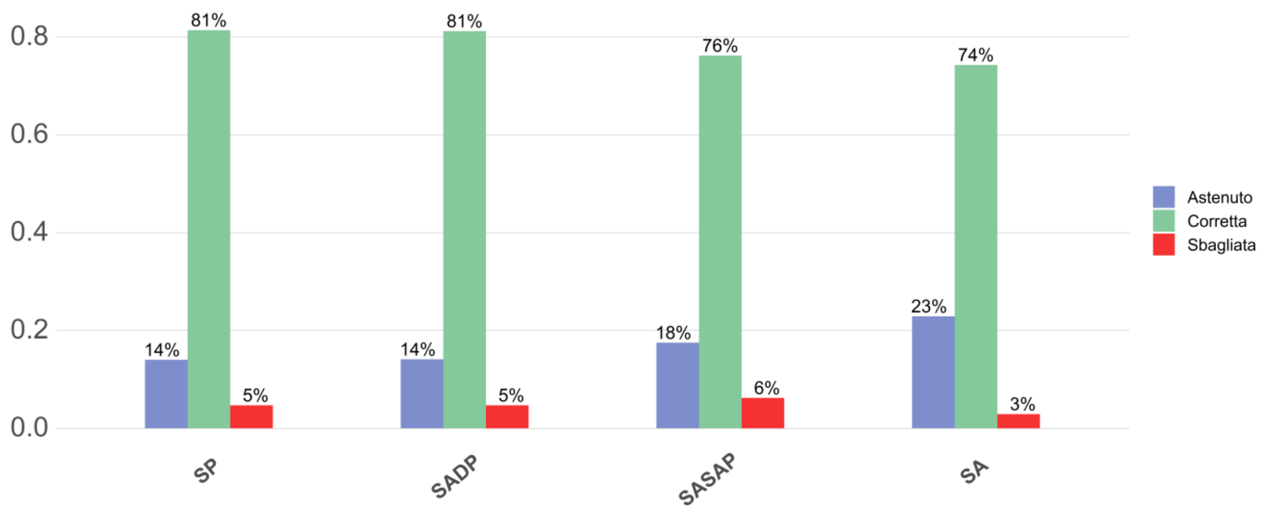

Struttura	Corrette	Sbagliate	Astenuto
**Struttura pediatrica**	35 (81,4%)	2 (4,7%)	6 (14%)
**Struttura per adulti con dipartimento pediatrico**	52 (81,2%)	3 (4,7%)	9 (14,1%)
**Struttura per adulti con sporadica attività pediatrica**	61 (76,2%)	5 (6,2%)	14 (17,5%)
**Struttura esclusivamente per adulti**	26 (74,3%)	1 (2,9%)	8 (22,9%)

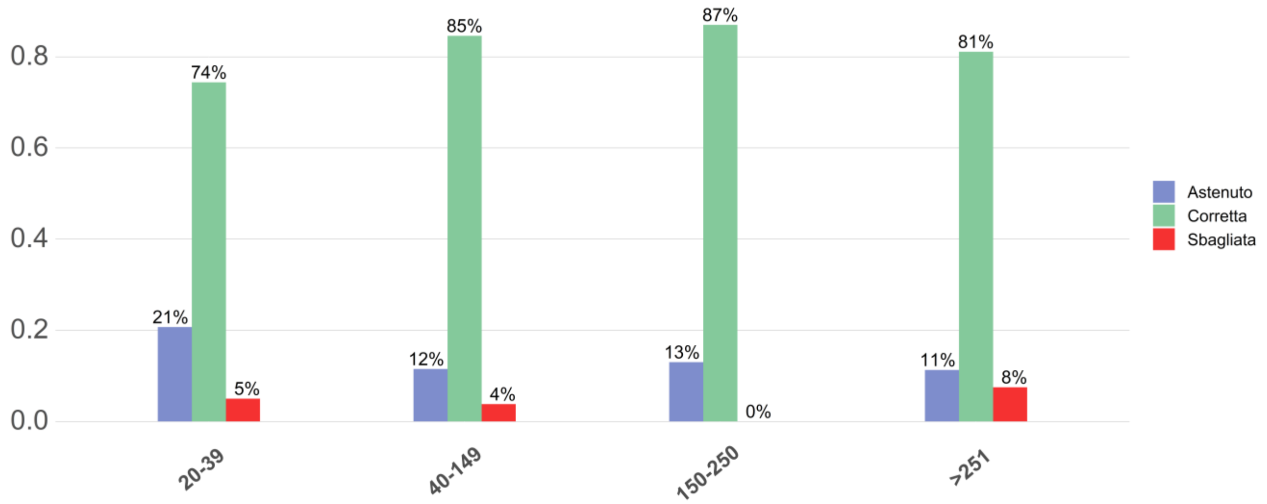

N. Anestesie Annue	Corrette	Sbagliate	Astenuto
**20-39**	90 (74,4%)	6 (5%)	25 (20,7%)
**40-149**	22 (84,6%)	1 (3,8%)	3 (11,5%)
**150-250**	20 (87%)	0 (0%)	3 (13%)
**>251**	43 (81,1%)	4 (7,5%)	6 (11,3%)

Osservando le distribuzioni delle risposte rispetto al tipo di struttura in cui esercita l'intervistato e all'esperienza (misurata da anzianità lavorativa e numero di anestesia annualmente praticate), non sembra esservi la presenza di una associazione. I risultati dell'applicazione di un modello di regressione logistica che vede come variabile dipendente la "risposta" (Corretta/Sbagliata), confermano l'assenza di una relazione di dipendenza (p>.05)

Diagnosi

10. Esami per la diagnosi di pOSAs

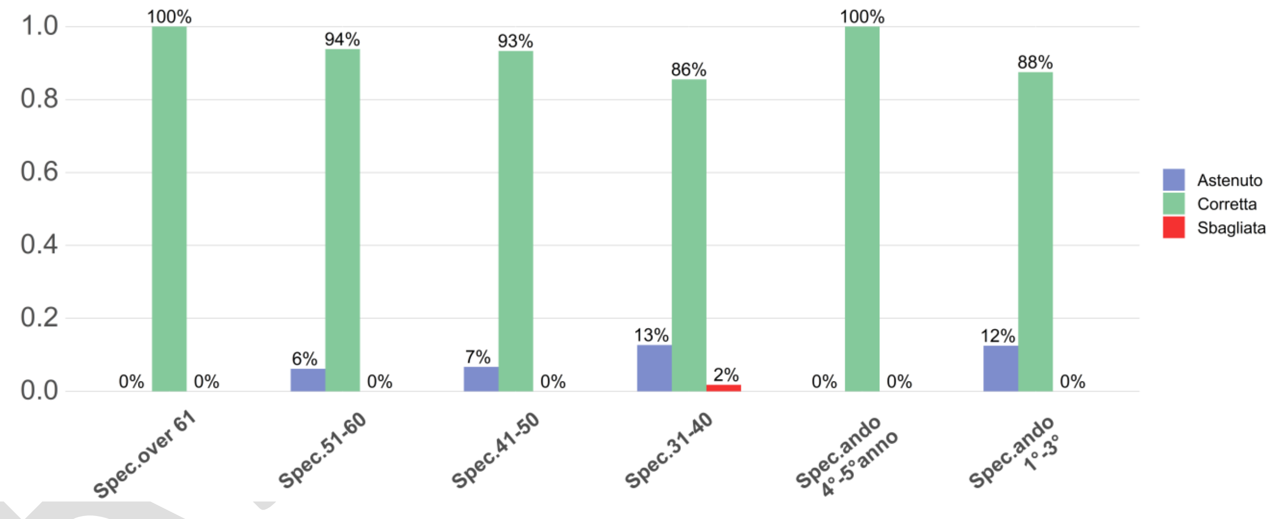

È stato chiesto agli intervistati se la polisonnografia (PSG) è l'esame ideale per la diagnosi di pOSAs, 205 intervistati (92%) ha risposto correttamente, 1 intervistato (0,4%) ha risposto erroneamente e 17 intervistati (7,6%) ha dichiarato di non conoscere la risposta. Si presentano in tabella le distribuzioni di frequenza delle risposte Corrette/Sbagliate e degli Astenuti, aggregate in accordo con le variabili di interesse.

Esperienza	Corrette	Sbagliate	Astenuto
**Specialista over 61**	19 (100%)	0 (0%)	0 (0%)
**Specialista 51-60**	30 (93,8%)	0 (0%)	2 (6,2%)
**Specialista 41-50**	70 (93,3%)	0 (0%)	5 (6,7%)
**Specialista 31-40**	47 (85,5%)	1 (1,8%)	7 (12,7%)
**Specializzando 4°-5° anno**	18 (100%)	0 (0%)	0 (0%)

Specializzando 1°-3° anno	21 (87,5%)	0 (0%)	3 (12,5%)

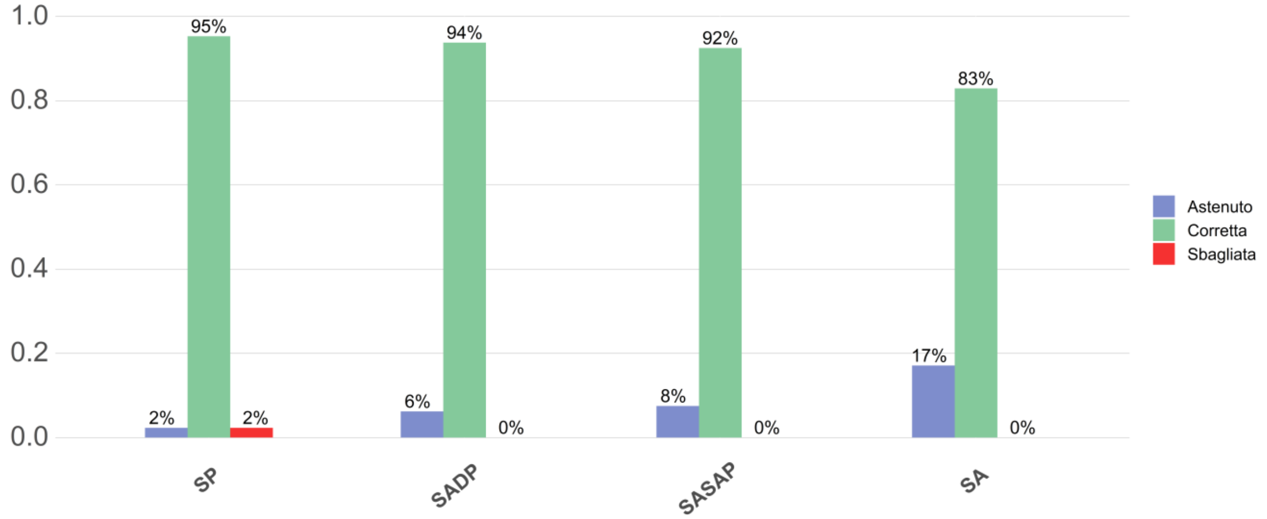

Struttura	Corrette	Sbagliate	Astenuto
Struttura pediatrica	41 (95,3%)	1 (2,3%)	1 (2,3%)
Struttura per adulti con dipartimento pediatrico	60 (93,8%)	0 (0%)	4 (6,2%)
Struttura per adulti con sporadica attività pediatrica	74 (92,5%)	0 (0%)	6 (7,5%)
Struttura esclusivamente per adulti	29 (82,9%)	0 (0%)	6 (17,1%)

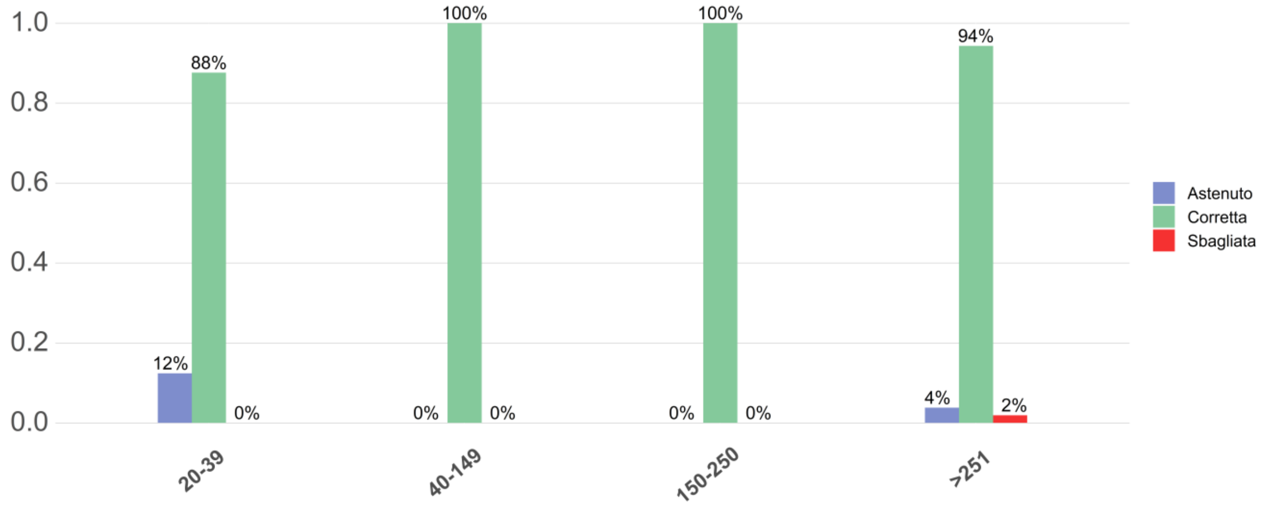

N. Anestesie Annue	Corrette	Sbagliate	Astenuto
20-39	106 (87,6%)	0 (0%)	15 (12,4%)
40-149	26 (100%)	0 (0%)	0 (0%)
150-250	23 (100%)	0 (0%)	0 (0%)
>251	50 (94,3%)	1 (1,9%)	2 (3,8%)

Osservando le distribuzioni delle risposte rispetto al tipo di struttura in cui esercita l’intervistato e all’esperienza (misurata da anzianità lavorativa e numero di anestesia annualmente praticate), non sembra esservi la presenza di una associazione. I risultati dell’applicazione di un modello di regressione logistica che vede come variabile dipendente la “risposta” (Corretta/Sbagliata), confermano l’assenza di una relazione di dipendenza ($p>.05$)

11. Visita otorinolaringoiatrica per esclusione di pOSAs

È stato chiesto agli intervistati se una accurata visita otorinolaringoiatrica può escludere la diagnosi si pOSAs, 13 intervistati (59%) ha risposto correttamente, 32 intervistati (14%) ha risposto erroneamente e 60 intervistati (27%) ha dichiarato di non conoscere la risposta. Si presentano in tabella le distribuzioni di frequenza delle risposte Corrette/Sbagliate e degli Astenuti, aggregate in accordo con le variabili di interesse.

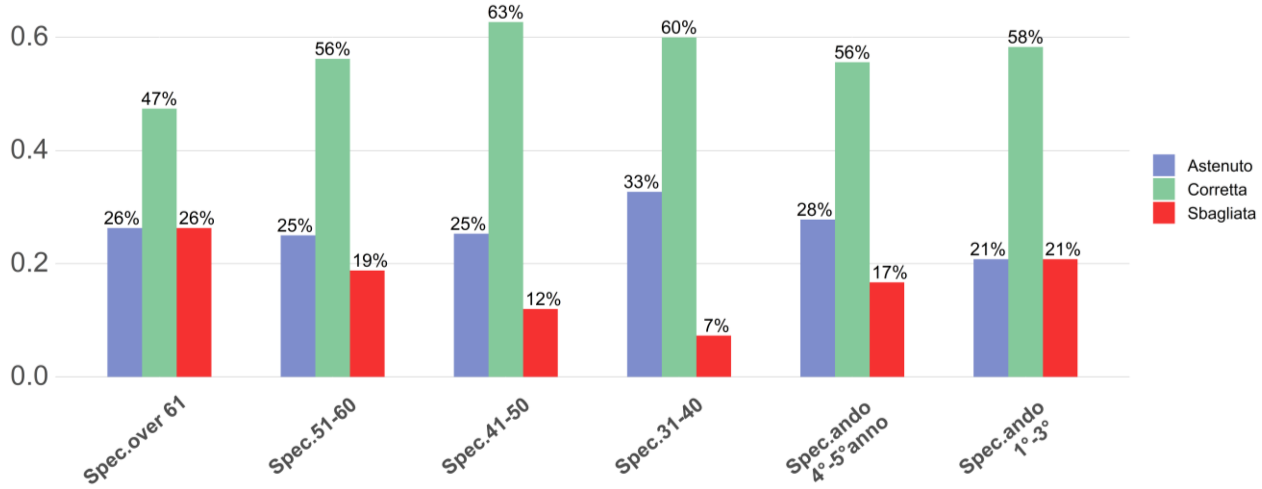

Esperienza	Corrette	Sbagliate	Astenuto
**Specialista over 61**	9 (47,4%)	5 (26,3%)	5 (26,3%)
**Specialista 51-60**	18 (56,2%)	6 (18,8%)	8 (25%)
**Specialista 41-50**	47 (62,7%)	9 (12%)	19 (25,3%)
**Specialista 31-40**	33 (60%)	4 (7,3%)	18 (32,7%)
**Specializzando 4°-5° anno**	10 (55,6%)	3 (16,7%)	5 (27,8%)
**Specializzando 1°-3° anno**	14 (58,3%)	5 (20,8%)	5 (20,8%)

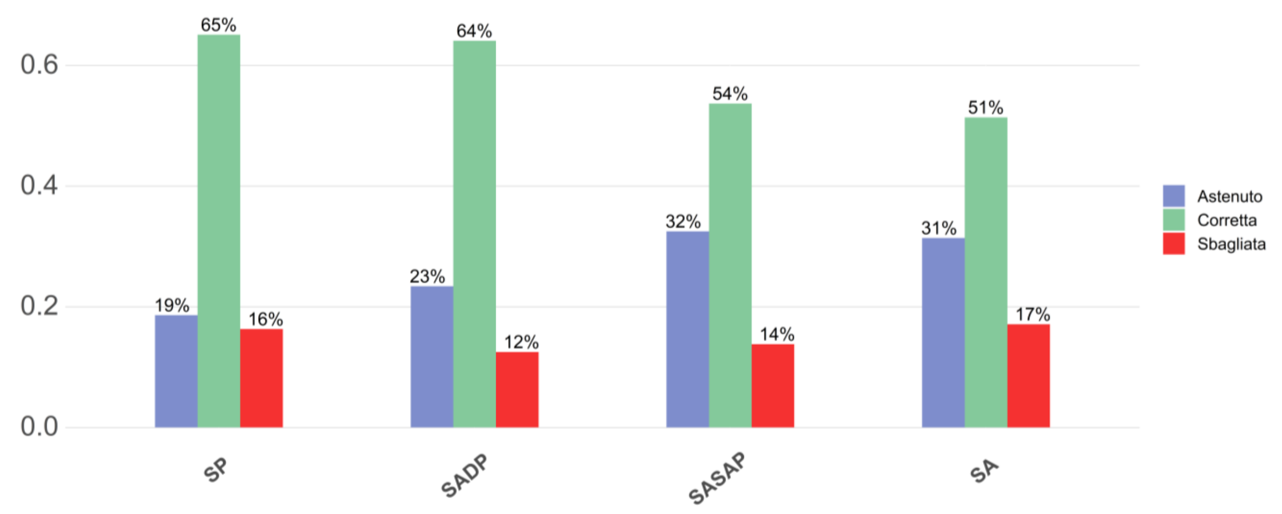

Struttura	Corrette	Sbagliate	Astenuto
**Struttura pediatrica**	28 (65,1%)	7 (16,3%)	8 (18,6%)
**Struttura per adulti con dipartimento pediatrico**	41 (64,1%)	8 (12,5%)	15 (23,4%)
**Struttura per adulti con sporadica attività pediatrica**	43 (53,7%)	11 (13,8%)	26 (32,5%)
**Struttura esclusivamente per adulti**	18 (51,4%)	6 (17,1%)	11 (31,4%)

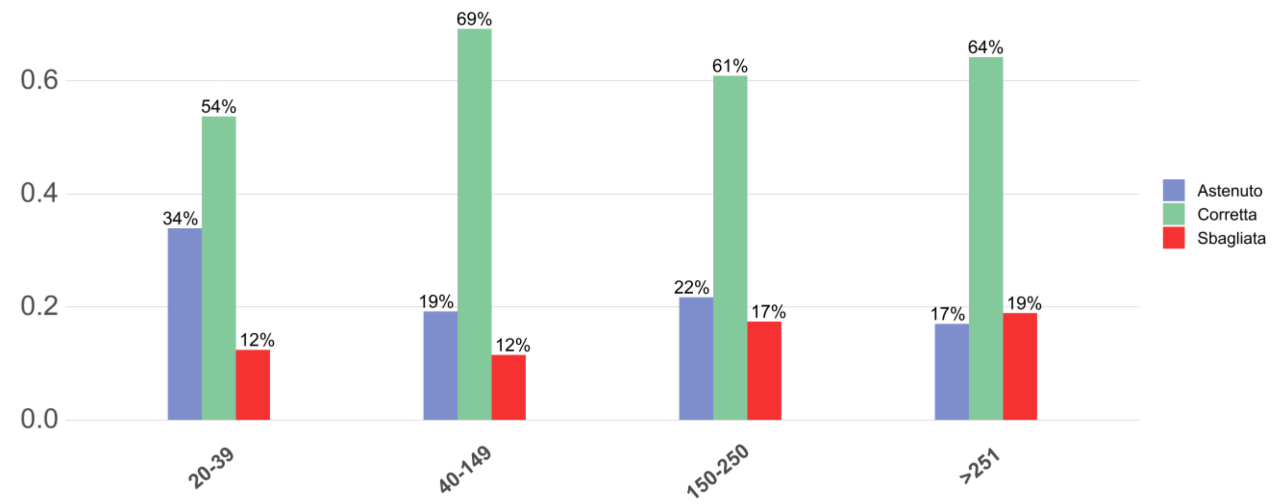

N. Anestesie Annue	Corrette	Sbagliate	Astenuto
**20-39**	65 (53,7%)	15 (12,4%)	41 (33,9%)
**40-149**	18 (69,2%)	3 (11,5%)	5 (19,2%)
**150-250**	14 (60,9%)	4 (17,4%)	5 (21,7%)
**>251**	34 (64,2%)	10 (18,9%)	9 (17%)

Osservando le distribuzioni delle risposte rispetto al tipo di struttura in cui esercita l'intervistato e all'esperienza (misurata da anzianità lavorativa e numero di anestesia annualmente praticate), non sembra esservi la presenza di una associazione. I risultati dell'applicazione di un modello di regressione logistica che

vede come variabile dipendente la "risposta" (Corretta/Sbagliata), confermano l'assenza di una relazione di dipendenza (p>.05)

12. Indice apnea/ipopnea nel bambino

È stato chiesto agli intervistati se un indice apnea/ipopnea 0-5 alla PSG è normale nel bambino, 38 intervistati (17%) ha risposto correttamente, 52 intervistati (23%) ha risposto erroneamente e 133 intervistati (60%) ha dichiarato di non conoscere la risposta. Si presentano in tabella le distribuzioni di frequenza delle risposte Corrette/Sbagliate e degli Astenuti, aggregate in accordo con le variabili di interesse.

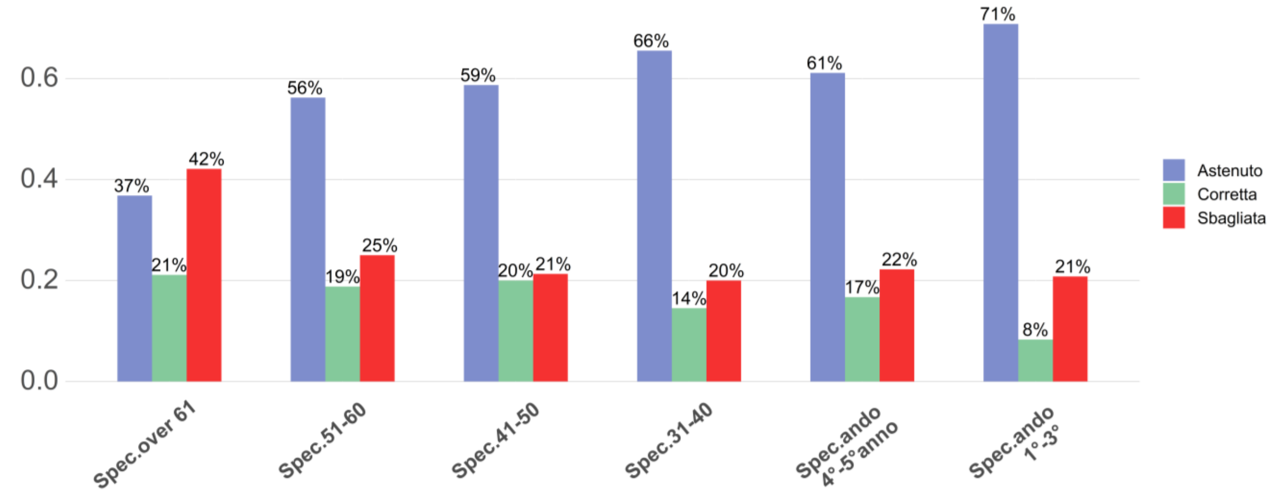

Esperienza	Corrette	Sbagliate	Astenuto
Specialista over 61	4 (21,1%)	8 (42,1%)	7 (36,8%)
Specialista 51-60	6 (18,8%)	8 (25%)	18 (56,2%)
Specialista 41-50	15 (20%)	16 (21,3%)	44 (58,7%)
Specialista 31-40	8 (14,5%)	11 (20%)	36 (65,5%)
Specializzando 4°-5° anno	3 (16,7%)	4 (22,2%)	11 (61,1%)
Specializzando 1°-3° anno	2 (8,3%)	5 (20,8%)	17 (70,8%)

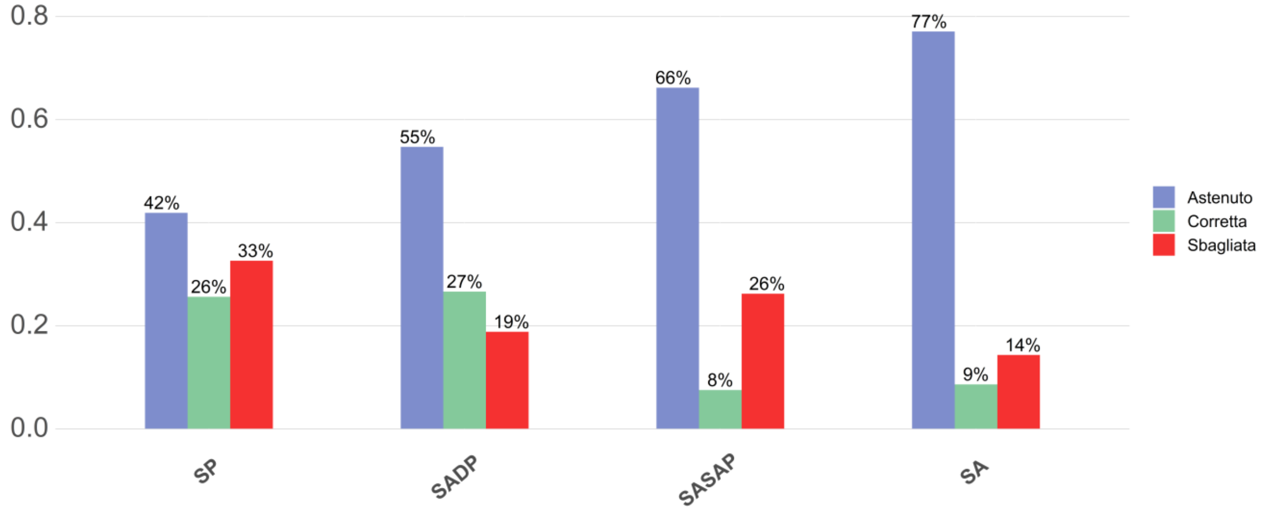

**Struttura**	**Corrette**	**Sbagliate**	**Astenuto**
**Struttura pediatrica**	11 (25,6%)	14 (32,6%)	18 (41,9%)
**Struttura per adulti con dipartimento pediatrico**	17 (26,6%)	12 (18,8%)	35 (54,7%)
**Struttura per adulti con sporadica attività pediatrica**	6 (7,5%)	21 (26,2%)	53 (66,2%)
**Struttura esclusivamente per adulti**	3 (8,6%)	5 (14,3%)	27 (77,1%)

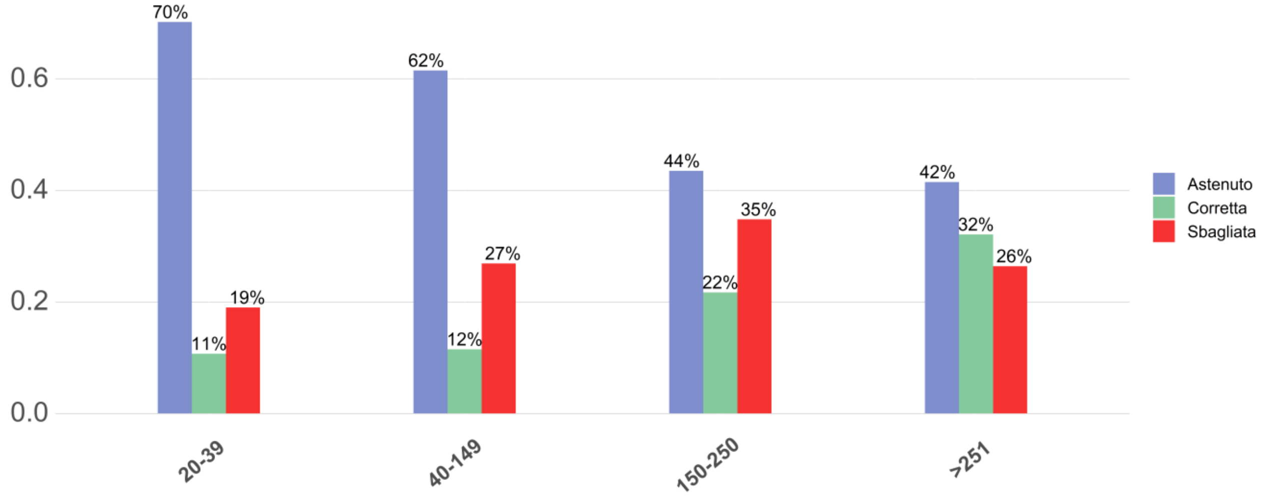

**N. Anestesie Annue**	**Corrette**	**Sbagliate**	**Astenuto**
**20-39**	13 (10,7%)	23 (19%)	85 (70,2%)
**40-149**	3 (11,5%)	7 (26,9%)	16 (61,5%)
**150-250**	5 (21,7%)	8 (34,8%)	10 (43,5%)
**>251**	17 (32,1%)	14 (26,4%)	22 (41,5%)

Osservando le distribuzioni delle risposte rispetto al tipo di struttura in cui esercita l’intervistato e all’esperienza (misurata da anzianità lavorativa e numero di anestesia annualmente praticate), non sembra esservi la presenza di una associazione. I risultati dell’applicazione di un modello di regressione logistica che vede come variabile dipendente la “risposta” (Corretta/Sbagliata), confermano l’assenza di una relazione di dipendenza ($p>.05$)

13. CPAP nella pOSAs

È stato chiesto agli intervistati se contrariamente all'adulto, CPAP non è mai utile nella pOSAs, 106 intervistati (48%) ha risposto correttamente, 28 intervistati (13%) ha risposto erroneamente e 89 intervistati (40%) ha dichiarato di non conoscere la risposta. Si presentano in tabella le distribuzioni di frequenza delle risposte Corrette/Sbagliate e degli Astenuti, aggregate in accordo con le variabili di interesse.

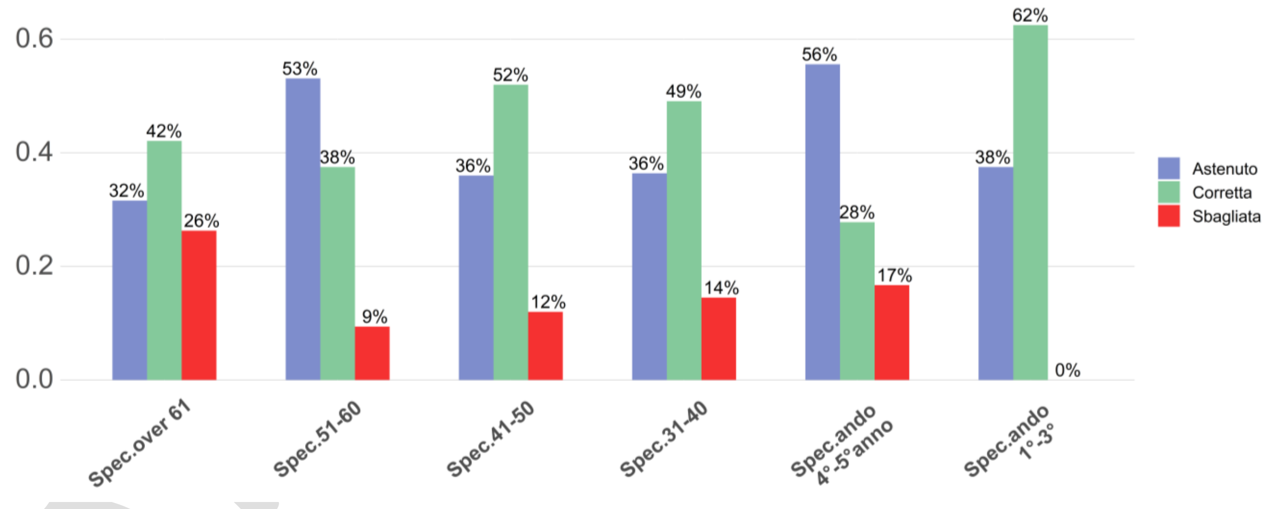

Esperienza	Corrette	Sbagliate	Astenuto
**Specialista over 61**	8 (42,1%)	5 (26,3%)	6 (31,6%)
**Specialista 51-60**	12 (37,5%)	3 (9,4%)	17 (53,1%)
**Specialista 41-50**	39 (52%)	9 (12%)	27 (36%)
**Specialista 31-40**	27 (49,1%)	8 (14,5%)	20 (36,4%)
**Specializzando 4°-5° anno**	5 (27,8%)	3 (16,7%)	10 (55,6%)
**Specializzando 1°-3° anno**	15 (62,5%)	0 (0%)	9 (37,5%)

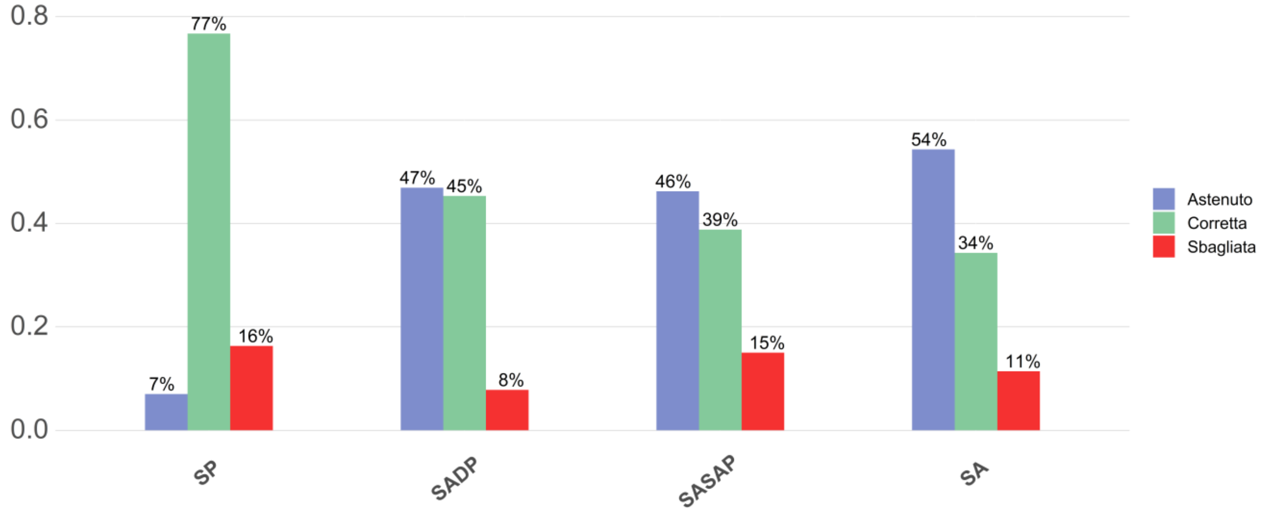

Struttura	Corrette	Sbagliate	Astenuto
**Struttura pediatrica**	33 (76,7%)	7 (16,3%)	3 (7%)
**Struttura per adulti con dipartimento pediatrico**	29 (45,3%)	5 (7,8%)	30 (46,9%)
**Struttura per adulti con sporadica attività pediatrica**	31 (38,8%)	12 (15%)	37 (46,2%)
**Struttura esclusivamente per adulti**	12 (34,3%)	4 (11,4%)	19 (54,3%)

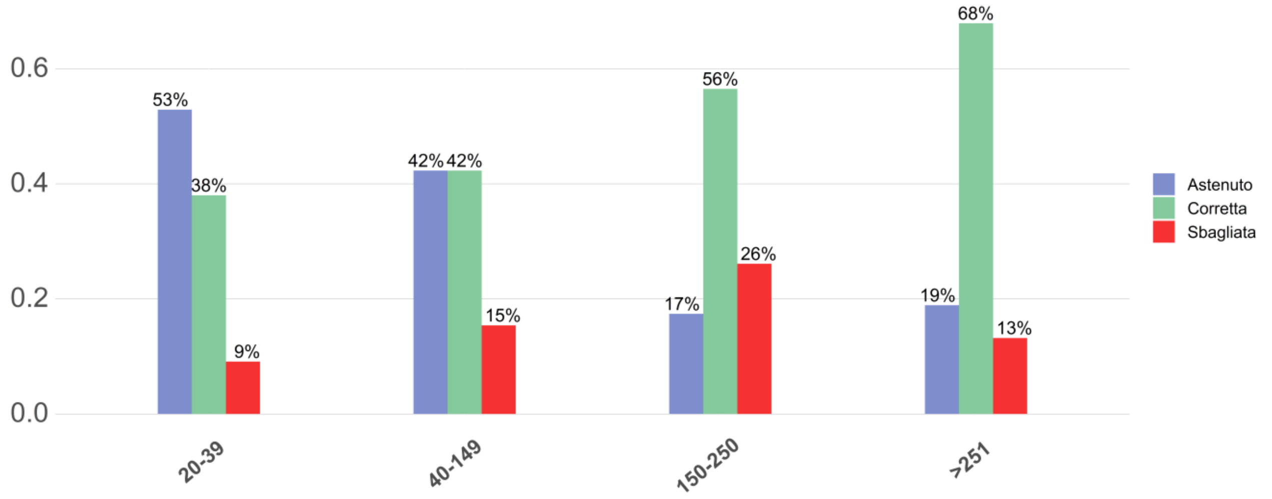

N. Anestesie Annue	Corrette	Sbagliate	Astenuto
**20-39**	46 (38%)	11 (9,1%)	64 (52,9%)
**40-149**	11 (42,3%)	4 (15,4%)	11 (42,3%)
**150-250**	13 (56,5%)	6 (26,1%)	4 (17,4%)
**>251**	36 (67,9%)	7 (13,2%)	10 (18,9%)

Osservando le distribuzioni delle risposte rispetto al numero di anestesia annualmente praticate annualmente, non sembra esservi la presenza di una associazione. I risultati dell’applicazione di un modello di regressione logistica che vede come variabile dipendente la “risposta” (Corretta/Sbagliata), confermano l’assenza di una relazione di dipendenza ($p > .05$)

Osservando le distribuzioni delle risposte rispetto al tipo di struttura in cui esercita l’intervistato e all’esperienza (misurata da anzianità lavorativa), non sembra esservi la presenza di una associazione. Dai

risultati dell'applicazione di un modello di regressione logistica che vede come variabile dipendente la "risposta" (Corretta/Sbagliata), si evince che è presente una relazione significativa di dipendenza.

Risposta Test	Esperienza	β	p-value	OR
Corretta	**Specialista over 61**	-24204	**0,04**	0,090
	**Specialista 51-60**	-21691	0,06	0,114
	**Specialista 41-50**	-18171	0,09	0,163
	**Specialista 31-40**	-0,7985	0,49	0,450
	**Specializzando 4°-5° anno**	-17918	0,18	0,167

Baseline per questionario: "Sbagliata", Baseline Esperienza: "specializzando 1-3 anno"

La quota degli Specialisti over 61 che hanno risposto correttamente al questionario è 0,91 volte inferiore la quota degli Specializzando 1°-3° anno che hanno risposto correttamente alla domanda. La probabilità di rispondere correttamente alle domande è dunque il 91% minore per gli specialisti over 61, rispetto agli specializzandi 1°-3° anno.

Risposta Test	Struttura	β	p-value	OR
Corretta	**Struttura esclusivamente per adulti**		**0,01**	0,161
	**Struttura per adulti con dipartimento pediatrico**		0,31	0,502
	**Struttura per adulti con sporadica attività pediatrica**		0,02	0,235

Baseline per questionario: "Sbagliata", Baseline per Struttura: "Struttura pediatrica"

La quota degli anestesisti che svolgono la propria attività presso una struttura esclusivamente per adulti, ed hanno risposto correttamente al questionario è 0,84 volte inferiore la quota anestesisti che svolgono la propria attività presso una struttura pediatrica che hanno risposto correttamente alla domanda. La probabilità di rispondere correttamente alle domande è dunque l' 84% minore chi dichiara di lavorare presso una struttura esclusivamente per adulti rispetto a chi lavora in una struttura pediatrica.

RISERVATO

14. Adenotonsillectomia e pOSAs

È stato chiesto agli intervistati se l'adenotonsillectomia è mandatoria in pOSAs moderata/severa, 42 intervistati (19%) ha risposto correttamente, 53 intervistati (24%) ha risposto erroneamente e 128 intervistati (58%) ha dichiarato di non conoscere la risposta. Si presentano in tabella le distribuzioni di frequenza delle risposte Corrette/Sbagliate e degli Astenuti, aggregate in accordo con le variabili di interesse.

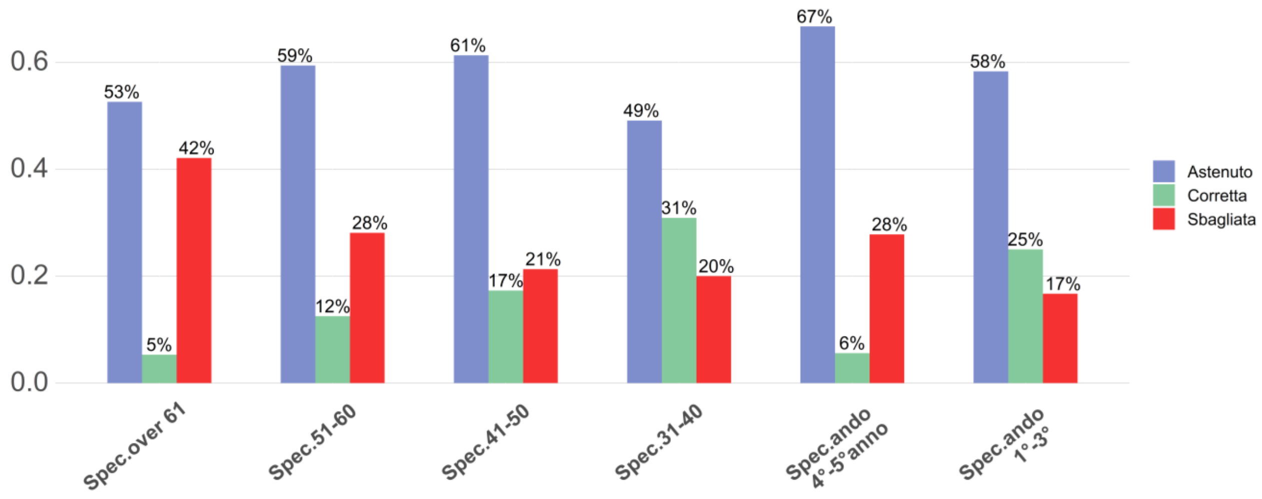

Esperienza	Corrette	Sbagliate	Astenuto
Specialista over 61	1 (5,3%)	8 (42,1%)	10 (52,6%)
Specialista 51-60	4 (12,5%)	9 (28,1%)	19 (59,4%)
Specialista 41-50	13 (17,3%)	16 (21,3%)	46 (61,3%)
Specialista 31-40	17 (30,9%)	11 (20%)	27 (49,1%)
Specializzando 4°-5° anno	1 (5,6%)	5 (27,8%)	12 (66,7%)
Specializzando 1°-3° anno	6 (25%)	4 (16,7%)	14 (58,3%)

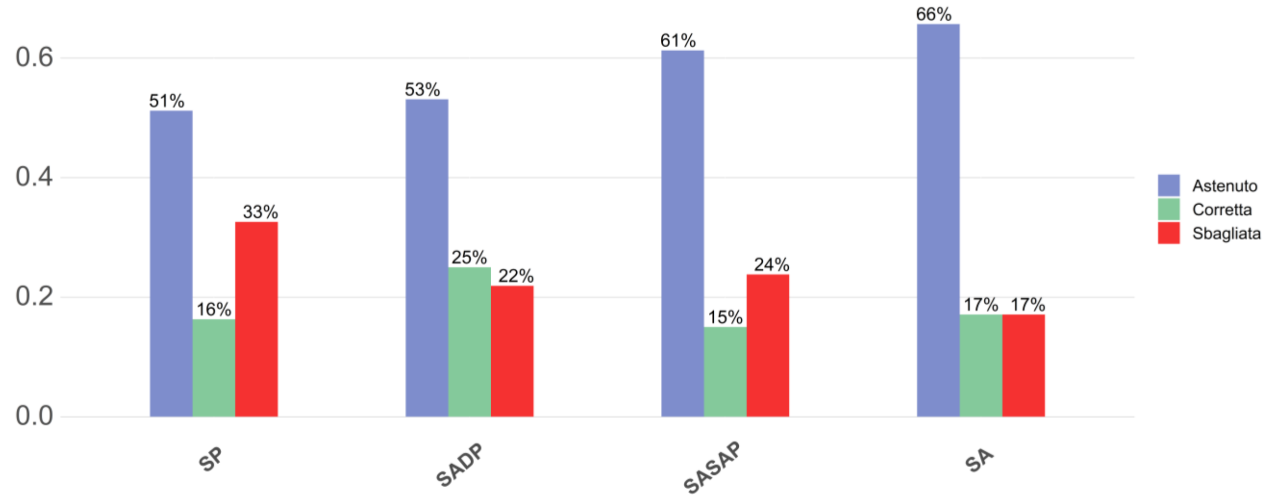

Struttura	Corrette	Sbagliate	Astenuto
Struttura pediatrica	7 (16,3%)	14 (32,6%)	22 (51,2%)
Struttura per adulti con dipartimento pediatrico	16 (25%)	14 (21,9%)	34 (53,1%)
Struttura per adulti con sporadica attività pediatrica	12 (15%)	19 (23,8%)	49 (61,3%)
Struttura esclusivamente per adulti	6 (17,1%)	6 (17,1%)	23 (65,7%)

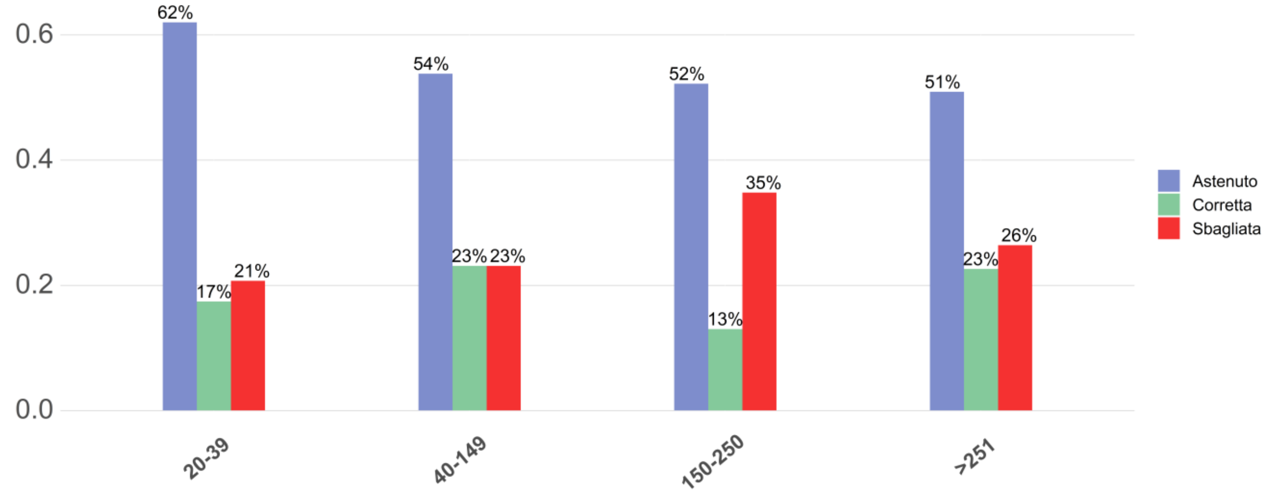

N. Anestesie Annue	Corrette	Sbagliate	Astenuto
20-39	21 (17,4%)	25 (20,7%)	75 (62%)
40-149	6 (23,1%)	6 (23,1%)	14 (53,8%)
150-250	3 (13%)	8 (34,8%)	12 (52,2%)
>251	12 (22,6%)	14 (26,4%)	27 (50,9%)

Osservando le distribuzioni delle risposte rispetto al tipo di struttura in cui esercita l'intervistato e all'esperienza (misurata da anzianità lavorativa e numero di anestesia annualmente praticate), non sembra esservi la presenza di una associazione. I risultati dell'applicazione di un modello di regressione logistica che vede come variabile dipendente la "risposta" (Corretta/Sbagliata), confermano l'assenza di una relazione di dipendenza (p>.05)

Tematiche di interesse per la formazione

È stato chiesto agli intervistati di esprimere una preferenza sulle tematiche inerenti al pOSAs, di interesse per un approfondimento in formazione. Con il fine di indagare se il tipo di struttura in cui esercita l'intervistato o l'esperienza (misurata da anzianità lavorativa e numero di anestesie annualmente praticate), possano influenzare la scelta di un argomento di approfondimento, è stato definito un indicatore di preferenza degli intervistati verso l'argomento di interesse che varia tra 0 e 1 (esprimendo massima accordo verso un giudizio positivo di preferenza se uguale a 1 e massimo accordo verso un giudizio negativo di preferenza se uguale a 0)

Indicatore di accordo sul tema "Diagnosi" come argomento di approfondimento

Diagnosi

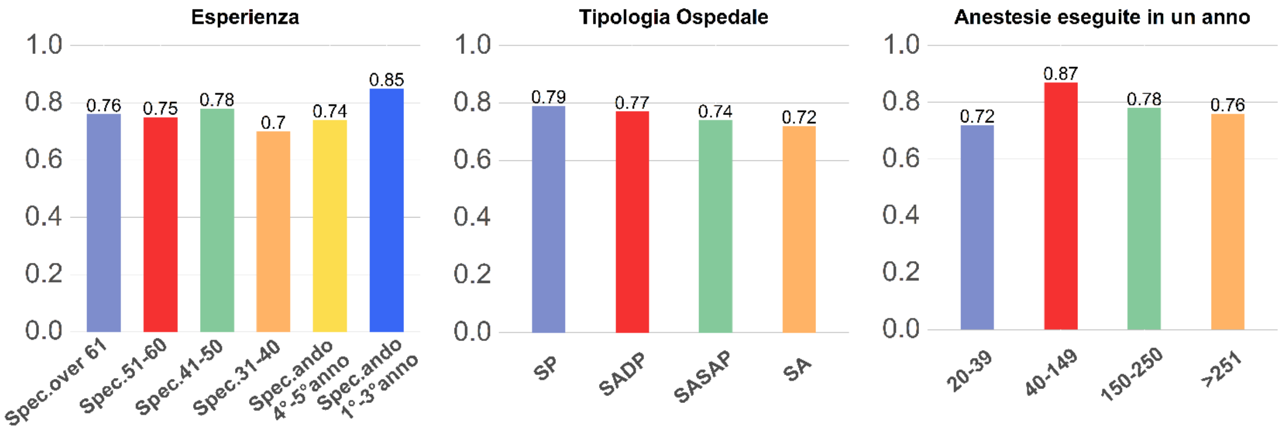

Esperienza	Per niente	Poco	Abbastanza	Molto	Moltissimo	Indicatore
**Specialista over 61**	0,0%	15,8%	21,1%	52,6%	10,5%	**0,76**
**Specialista 51- 60**	0,0%	9,4%	46,9%	34,4%	9,4%	**0,75**
**Specialista 41- 50**	0,0%	9,3%	36,0%	36,0%	18,7%	**0,78**
**Specialista 31- 40**	1,8%	10,9%	43,6%	32,7%	10,9%	**0,70**
**Specializzando 4°-5° anno**	0,0%	5,6%	66,7%	16,7%	11,1%	**0,74**

**Specializzando 1°-3° anno**	0,0%	0,0%	41,7%	41,7%	16,7%	**0,85**

Struttura	Per niente	Poco	Abbastanza	Molto	Moltissimo	Indicatore
**Pediatrica**	0,0%	2,3%	53,5%	30,2%	14,0%	**0,79**
**Per adulti con dipartimento pediatrico**	0,0%	9,4%	40,6%	31,2%	18,8%	**0,77**
**Per adulti con sporadica attività pediatrica**	1,3%	8,8%	35,0%	45,0%	10,0%	**0,74**
**Esclusivamente per adulti**	0,0%	17,1%	42,9%	25,7%	14,3%	**0,72**

N. Anestesie Annue	Per niente	Poco	Abbastanza	Molto	Moltissimo	Indicatore
**20-39**	0,8%	11,6%	39,7%	36,4%	11,6%	**0,72**
**40-149**	0,0%	0,0%	34,6%	42,3%	23,1%	**0,87**
**150-250**	0,0%	4,3%	56,5%	21,7%	17,4%	**0,78**
**>251**	0,0%	9,4%	41,5%	35,8%	13,2%	**0,76**

Gli specializzandi 1°-3° anno e in generale gli anestesisti che hanno praticato un numero di anestesie annue tra 41-149 sembrano essere maggiormente interessati alla formazione sulla diagnosi della pOSAs.

Indicatore di accordo sul tema "Stratificazione del rischio" come argomento di approfondimento

Stratificazione del rischio

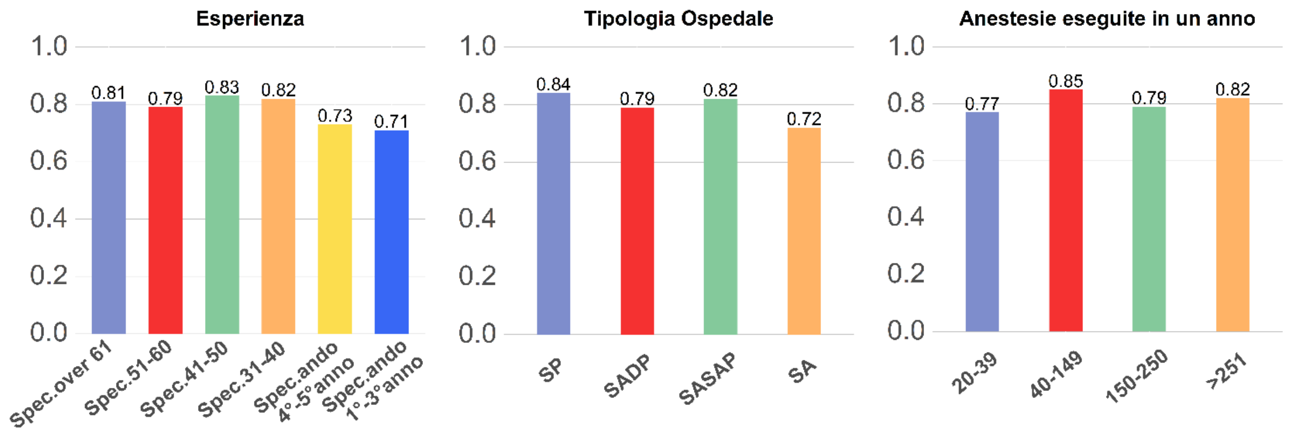

Esperienza	Per niente	Poco	Abbastanza	Molto	Moltissimo	Indicatore
**Specialista over 61**	0,0%	10,5%	15,8%	52,6%	21,1%	**0,81**
**Specialista 51- 60**	0,0%	6,2%	40,6%	37,5%	15,6%	**0,79**
**Specialista 41- 50**	0,0%	4,0%	30,7%	41,3%	24,0%	**0,83**
**Specialista 31- 40**	0,0%	5,5%	29,1%	41,8%	23,6%	**0,82**
**Specializzando 4°-5° anno**	0,0%	16,7%	38,9%	27,8%	16,7%	**0,73**
**Specializzando 1°-3° anno**	4,2%	4,2%	45,8%	33,3%	12,5%	**0,71**

Struttura	Per niente	Poco	Abbastanza	Molto	Moltissimo	Indicatore
**Pediatrica**	0,0%	2,3%	32,6%	41,9%	23,3%	**0,84**
**Per adulti con dipartimento pediatrico**	0,0%	10,9%	31,2%	31,2%	26,6%	**0,79**
**Per adulti con sporadica attività pediatrica**	0,0%	3,8%	31,2%	50,0%	15,0%	**0,82**
**Esclusivamente per adulti**	2,9%	8,6%	40,0%	28,6%	20,0%	**0,72**

N. Anestesie Annue	Per niente	Poco	Abbastanza	Molto	Moltissimo	Indicatore
**20-39**	0,8%	5,8%	38,8%	38,0%	16,5%	**0,77**
**40-149**	0,0%	7,7%	11,5%	50,0%	30,8%	**0,85**
**150-250**	0,0%	8,7%	39,1%	26,1%	26,1%	**0,79**
**>251**	0,0%	5,7%	26,4%	45,3%	22,6%	**0,82**

Lo specialista rispetto allo specializzando sembra essere maggiormente interessato alla formazione sulla stratificazione del rischio in pOSAs. Gli intervistati che lavorano presso una struttura con dipartimento pediatrico o in strutture pediatriche sembrerebbero più interessati alla formazione sulla stratificazione del rischio rispetto in pOSAs a chi lavora in una struttura per adulti. Gli anestesisti che hanno effettuato tra le 40 e le 149 anestesie sembrerebbero maggiormente interessati alla formazione sulla stratificazione del rischio in pOSAs.

Indicatore di accordo sul tema "Trattamento e gestione" come argomento di approfondimento

Trattamento e gestione

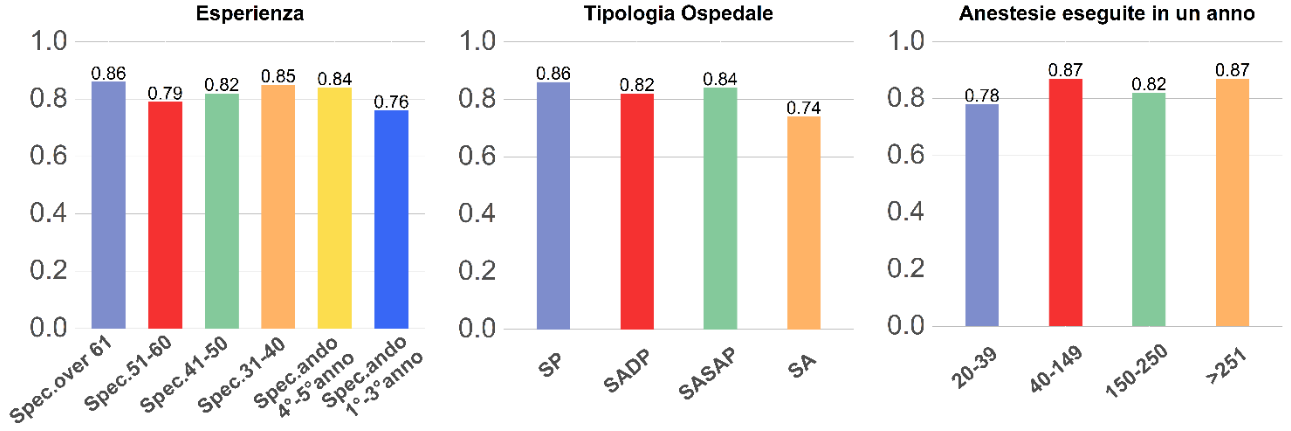

Esperienza	Per niente	Poco	Abbastanza	Molto	Moltissimo	Indicatore
**Specialista over 61**	0,0%	5,3%	15,8%	47,4%	31,6%	**0,86**
**Specialista 51- 60**	0,0%	6,2%	40,6%	31,2%	21,9%	**0,79**
**Specialista 41- 50**	0,0%	6,7%	26,7%	42,7%	24,0%	**0,82**
**Specialista 31- 40**	0,0%	7,3%	16,4%	36,4%	40,0%	**0,85**
**Specializzando 4°-5° anno**	0,0%	5,6%	22,2%	38,9%	33,3%	**0,84**
**Specializzando 1°-3° anno**	4,2%	8,3%	16,7%	45,8%	25,0%	**0,76**

Struttura	Per niente	Poco	Abbastanza	Molto	Moltissimo	Indicatore
**Pediatrica**	0,0%	2,3%	25,6%	41,9%	30,2%	**0,86**
**Per adulti con dipartimento pediatrico**	0,0%	7,8%	23,4%	39,1%	29,7%	**0,82**
**Per adulti con sporadica attività pediatrica**	0,0%	5,0%	26,2%	37,5%	31,2%	**0,84**
**Esclusivamente per adulti**	2,9%	14,3%	17,1%	42,9%	22,9%	**0,74**

N. Anestesie Annue	Per niente	Poco	Abbastanza	Molto	Moltissimo	Indicatore
**20-39**	0,8%	9,1%	25,6%	38,0%	26,4%	**0,78**
**40-149**	0,0%	7,7%	11,5%	34,6%	46,2%	**0,87**
**150-250**	0,0%	4,3%	34,8%	34,8%	26,1%	**0,82**
**>251**	0,0%	1,9%	20,8%	49,1%	28,3%	**0,87**

Tutti gli anestesisti sembrerebbero interessati alla formazione sul trattamento e la gestione della pOSAs.. Gli anestesisti che svolgono la propria attività presso una struttura pediatrica o struttura per adulti con sporadica attività pediatrica sembrerebbero maggiormente interessati alla formazione sul trattamento e la gestione della pOSAs.

Indicatore di accordo sul tema "Decorso post-operatorio" come argomento di approfondimento

Decorso post-operatorio

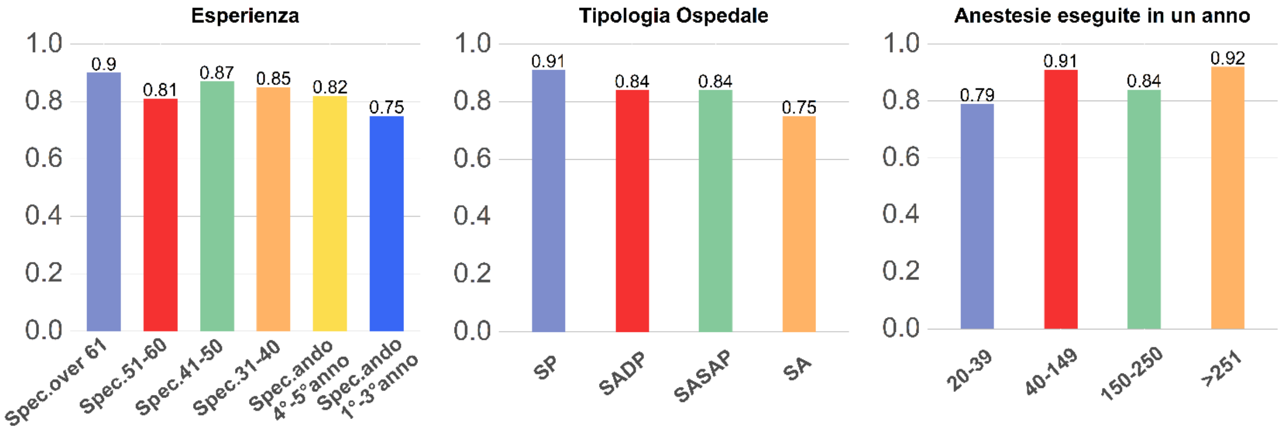

Esperienza	Per niente	Poco	Abbastanza	Molto	Moltissimo	Indicatore
**Specialista over 61**	0,0%	0,0%	21,1%	42,1%	36,8%	**0,90**
**Specialista 51- 60**	0,0%	9,4%	21,9%	43,8%	25,0%	**0,81**
**Specialista 41- 50**	0,0%	2,7%	20,0%	45,3%	32,0%	**0,87**
**Specialista 31- 40**	1,8%	1,8%	14,5%	43,6%	38,2%	**0,85**
**Specializzando 4°-5° anno**	0,0%	11,1%	16,7%	44,4%	27,8%	**0,82**
**Specializzando 1°-3° anno**	4,2%	12,5%	8,3%	54,2%	20,8%	**0,75**

Struttura	Per niente	Poco	Abbastanza	Molto	Moltissimo	Indicatore
**Pediatrica**	0,0%	0,0%	16,3%	51,2%	32,6%	**0,91**
**Per adulti con dipartimento pediatrico**	1,6%	3,1%	15,6%	43,8%	35,9%	**0,84**
**Per adulti con sporadica attività pediatrica**	0,0%	7,5%	16,2%	46,2%	30,0%	**0,84**
**Esclusivamente per adulti**	2,9%	8,6%	25,7%	37,1%	25,7%	**0,75**

N. Anestesie Annue	Per niente	Poco	Abbastanza	Molto	Moltissimo	Indicatore
**20-39**	1,7%	8,3%	17,4%	47,9%	24,8%	**0,79**
**40-149**	0,0%	0,0%	19,2%	34,6%	46,2%	**0,91**
**150-250**	0,0%	4,3%	34,8%	21,7%	39,1%	**0,84**
**>251**	0,0%	0,0%	9,4%	54,7%	35,8%	**0,92**

Tutti gli anestesisti, ad eccezione degli specialisti 1° -3° anno, sembrerebbero interessati alla formazione sul decorso post-operatorio in pOSAs. Chi lavora in una struttura pediatrica sembrerebbe maggiormente interessato alla formazione sul decorso post-operatorio in pOSAs. Gli anestesisti che hanno svolto 49-149 e >251 anestesie annue sembrerebbero maggiormente interessati alla formazione sul decorso post-operatorio in pOSAs

Indicatore di accordo sul tema "NIV in pOSAs" come argomento di approfondimento

NIV in pOSAs

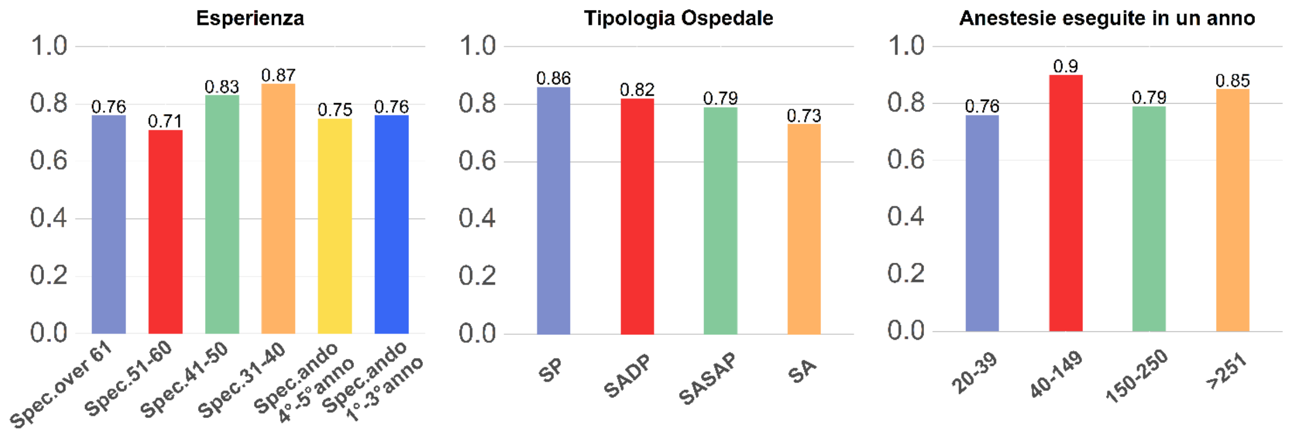

Esperienza	Per niente	Poco	Abbastanza	Molto	Moltissimo	Indicatore
**Specialista over 61**	0,0%	10,5%	36,8%	42,1%	10,5%	**0,76**
**Specialista 51- 60**	3,1%	12,5%	28,1%	40,6%	15,6%	**0,71**
**Specialista 41- 50**	0,0%	2,7%	28,0%	53,3%	16,0%	**0,83**
**Specialista 31- 40**	0,0%	3,6%	18,2%	36,4%	41,8%	**0,87**
**Specializzando 4°-5° anno**	5,6%	5,6%	27,8%	27,8%	33,3%	**0,75**
**Specializzando 1°-3° anno**	4,2%	8,3%	16,7%	41,7%	29,2%	**0,76**

Struttura	Per niente	Poco	Abbastanza	Molto	Moltissimo	Indicatore
**Pediatrica**	0,0%	2,3%	18,6%	55,8%	23,3%	**0,86**
**Per adulti con dipartimento pediatrico**	0,0%	7,8%	26,6%	35,9%	29,7%	**0,82**
**Per adulti con sporadica attività pediatrica**	1,3%	5,0%	31,2%	38,8%	23,8%	**0,79**
**Esclusivamente per adulti**	5,7%	8,6%	17,1%	48,6%	20,0%	**0,73**

N. Anestesie Annue	Per niente	Poco	Abbastanza	Molto	Moltissimo	Indicatore
**20-39**	2,5%	7,4%	24,8%	43,0%	22,3%	**0,76**
**40-149**	0,0%	0,0%	19,2%	50,0%	30,8%	**0,90**
**150-250**	0,0%	8,7%	39,1%	26,1%	26,1%	**0,79**
**>251**	0,0%	3,8%	22,6%	47,2%	26,4%	**0,85**

Gli specialisti 41-50 e gli specialisti 51-60 sembrerebbero maggiormente interessati sulla formazione in NIV in pOSAs. Chi lavora in una struttura pediatrica e struttura per adulti con dipartimento pediatrico sembrerebbe maggiormente interessato sulla formazione in NIV in pOSAs. Gli anestesisti che hanno svolto 49-149 e >251 anestesie annue sembrerebbero maggiormente interessati sulla formazione in NIV in pOSAs.

Sezione 2 – Atteggiamento in pOSAs

È stato chiesto agli intervistati di esprimere il proprio grado di accordo o disaccordo rispetto ad alcune affermazioni proposte. Ai fini di un confronto tra risposte aggregate per esperienza, tipologia di ospedale ed il numero di anestesie eseguite in un anno è stato calcolato un indicatore che misuri l'accordo degli intervistati verso un giudizio dell'Item che va da "Fortemente in disaccordo" a "Fortemente d'accordo" (massima concordanza verso il giudizio peggiore quando l'indicatore è uguale a 0, massima concordanza verso il giudizio migliore quando è uguale a 1).

Importanza del disturbo clinico e stratificazione del rischio anestesiologico

1- pOSAs severa può causare ipertensione polmonare è quindi indicata valutazione cardiologia

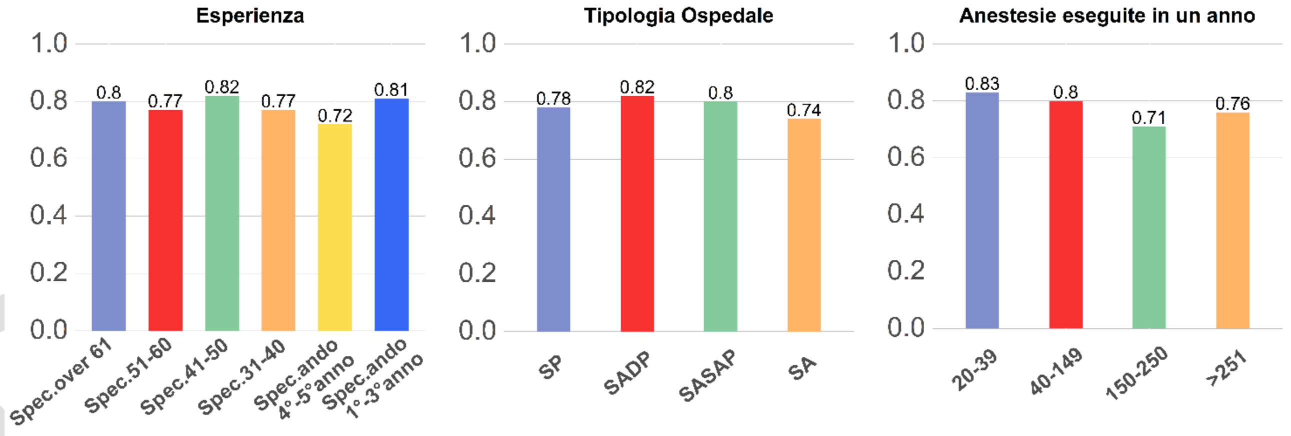

Esperienza	Fortemente disaccordo	Disaccordo	Né d'accordo né disaccordo	D'accordo	Fortemente d'accordo	Indicatore
**Specialista over 61**	5,3%	0,0%	15,8%	57,9%	21,1%	**0,80**
**Specialista 51- 60**	3,1%	3,1%	28,1%	50,0%	15,6%	**0,77**
**Specialista 41- 50**	0,0%	6,7%	18,7%	61,3%	13,3%	**0,82**
**Specialista 31- 40**	1,8%	7,3%	21,8%	56,4%	12,7%	**0,77**
**Specializzando 4°-5° anno**	5,6%	5,6%	22,2%	61,1%	5,6%	**0,72**
**Specializzando 1°-3° anno**	0,0%	8,3%	20,8%	58,3%	12,5%	**0,81**

Struttura	Fortemente disaccordo	Disaccordo	Né d'accordo né disaccordo	D'accordo	Fortemente d'accordo	Indicatore

**Pediatrica**	4,7%	2,3%	18,6%	53,5%	20,9%	**0,78**
**Per adulti con dipartimento pediatrico**	0,0%	4,7%	25,0%	54,7%	15,6%	**0,82**
**Per adulti con sporadica attività pediatrica**	0,0%	10,0%	18,8%	62,5%	8,8%	**0,80**
**Esclusivamente per adulti**	5,7%	2,9%	22,9%	57,1%	11,4%	**0,74**

N. Anestesie Annue	Fortemente disaccordo	Disaccordo	Né d'accordo né disaccordo	D'accordo	Fortemente d'accordo	Indicatore
**20-39**	0,0%	3,3%	24,8%	62,8%	9,1%	**0,83**
**40-149**	0,0%	15,4%	11,5%	50,0%	23,1%	**0,80**
**150-250**	4,3%	8,7%	30,4%	43,5%	13,0%	**0,71**
**>251**	5,7%	5,7%	13,2%	56,6%	18,9%	**0,76**

Tutti gli anestesisti sembrerebbero concordare con l'affermazione proposta.

2- I bambini affetti da OSAs presentano un rischio anestesiologico maggiore e vanno indirizzati a centri dotati di Terapia Intensiva Pediatrica

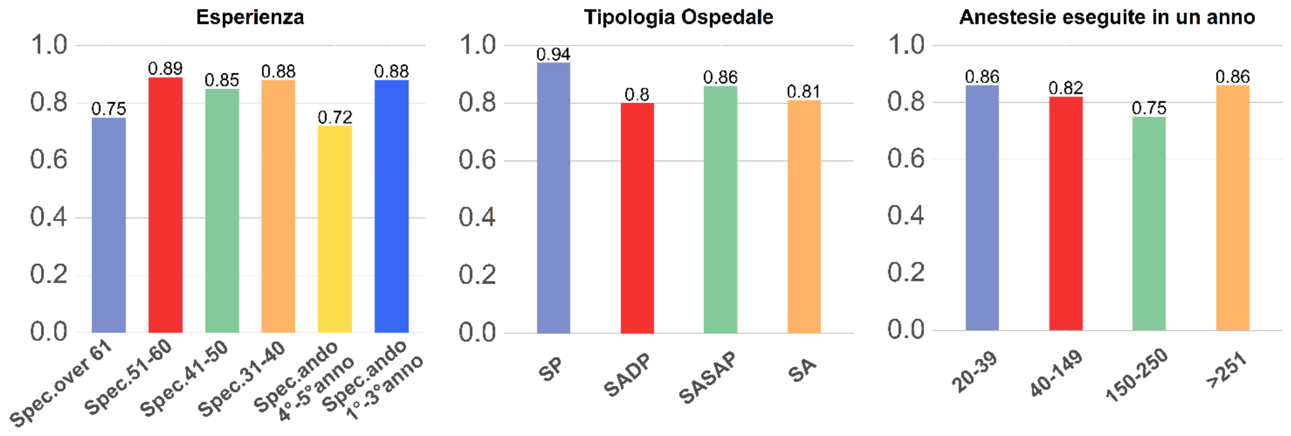

Esperienza	Fortemente disaccordo	Disaccordo	Né d'accordo né disaccordo	D'accordo	Fortemente d'accordo	Indicatore
**Specialista over 61**	5,3%	10,5%	10,5%	47,4%	26,3%	**0,75**
**Specialista 51- 60**	0,0%	3,1%	6,2%	62,5%	28,1%	**0,89**
**Specialista 41- 50**	0,0%	8,1%	13,5%	43,2%	35,1%	**0,85**
**Specialista 31- 40**	0,0%	3,6%	10,9%	47,3%	38,2%	**0,88**
**Specializzando 4°-5° anno**	5,6%	5,6%	22,2%	61,1%	5,6%	**0,72**
**Specializzando 1°-3° anno**	0,0%	0,0%	29,2%	45,8%	25,0%	**0,88**

Struttura	Fortemente disaccordo	Disaccordo	Né d'accordo né disaccordo	D'accordo	Fortemente d'accordo	Indicatore
**Pediatrica**	0,0%	0,0%	4,7%	48,8%	46,5%	**0,94**
**Per adulti con dipartimento pediatrico**	0,0%	9,4%	26,6%	42,2%	21,9%	**0,8**
**Per adulti con sporadica attività pediatrica**	0,0%	5,1%	12,7%	54,4%	27,8%	**0,86**
**Esclusivamente per adulti**	5,7%	2,9%	5,7%	51,4%	34,3%	**0,81**

N. Anestesie Annue	Fortemente disaccordo	Disaccordo	Né d'accordo né disaccordo	D'accordo	Fortemente d'accordo	Indicatore
**20-39**	0,0%	4,1%	16,5%	49,6%	29,8%	**0,86**
**40-149**	0,0%	8,0%	16,0%	60,0%	16,0%	**0,82**
**150-250**	4,3%	8,7%	21,7%	39,1%	26,1%	**0,75**

**>251**	1,9%	5,7%	3,8%	47,2%	41,5%	**0,86**

Tutti gli anestesisti sembrerebbero concordare con l'affermazione proposta.
Tutti gli anestesisti che svolgono la loro attività presso una struttura pediatrica sembrerebbero concordare con l'affermazione proposta.

Importanza dell'identificazione del disturbo

3- Il bambino affetto da OSAs sottoposto ad adenotonsillectomia necessita di particolari accorgimenti postoperatori

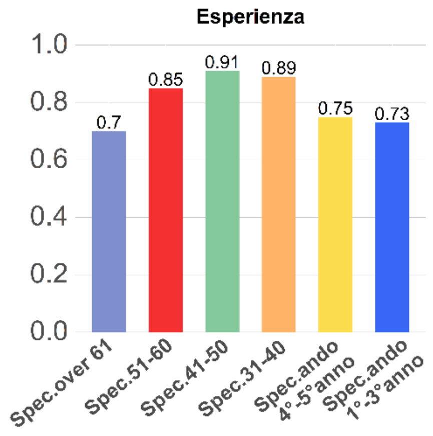

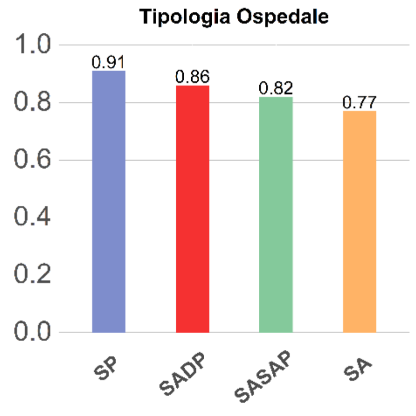

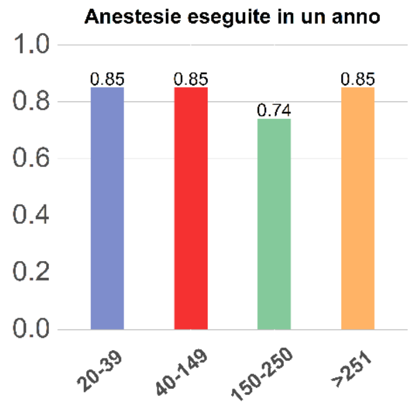

Esperienza	Fortemente disaccordo	Disaccordo	Né d'accordo né disaccordo	D'accordo	Fortemente d'accordo	Indicatore
**Specialista over 61**	10,5%	5,3%	15,8%	52,6%	15,8%	**0,70**
**Specialista 51- 60**	0,0%	9,4%	6,2%	59,4%	25,0%	**0,85**
**Specialista 41- 50**	0,0%	1,3%	5,3%	62,7%	30,7%	**0,91**
**Specialista 31- 40**	0,0%	1,9%	9,3%	61,1%	27,8%	**0,89**
**Specializzando 4°-5° anno**	5,6%	5,6%	5,6%	83,3%	0,0%	**0,75**
**Specializzando 1°-3° anno**	4,2%	4,2%	37,5%	37,5%	16,7%	**0,73**

Struttura	Fortemente disaccordo	Disaccordo	Né d'accordo né disaccordo	D'accordo	Fortemente d'accordo	Indicatore
**Pediatrica**	0,0%	0,0%	14,0%	51,2%	34,9%	**0,91**
**Per adulti con dipartimento pediatrico**	0,0%	6,2%	6,2%	67,2%	20,3%	**0,86**
**Per adulti con sporadica attività pediatrica**	1,3%	5,0%	13,8%	58,8%	21,2%	**0,82**
**Esclusivamente per adulti**	8,8%	0,0%	8,8%	61,8%	20,6%	**0,77**

N. Anestesie Annue	Fortemente disaccordo	Disaccordo	Né d'accordo né disaccordo	D'accordo	Fortemente d'accordo	Indicatore
**20-39**	0,8%	2,5%	11,7%	67,5%	17,5%	**0,85**
**40-149**	0,0%	7,7%	11,5%	53,8%	26,9%	**0,85**
**150-250**	8,7%	4,3%	8,7%	60,9%	17,4%	**0,74**
**>251**	1,9%	3,8%	9,4%	45,3%	39,6%	**0,85**

Gli specialisti over 41-50 e gli specialisti 51-60 sembrerebbero concordare nell'adottare particolari accorgimenti nel bambino affetto da OSAs sottoposto ad adenotonsillectomia.

Gli anestesisti che svolgono la loro attività in una struttura pediatrica sembrerebbero concordare nell'adottare particolari accorgimenti nel bambino affetto da OSAs sottoposto ad adenotonsillectomia.

Appropriatezza farmacologica

4- Gli analgesici oppioidi possono essere utilizzati con tranquillità a dosaggio standard nel periodo intraoperatorio

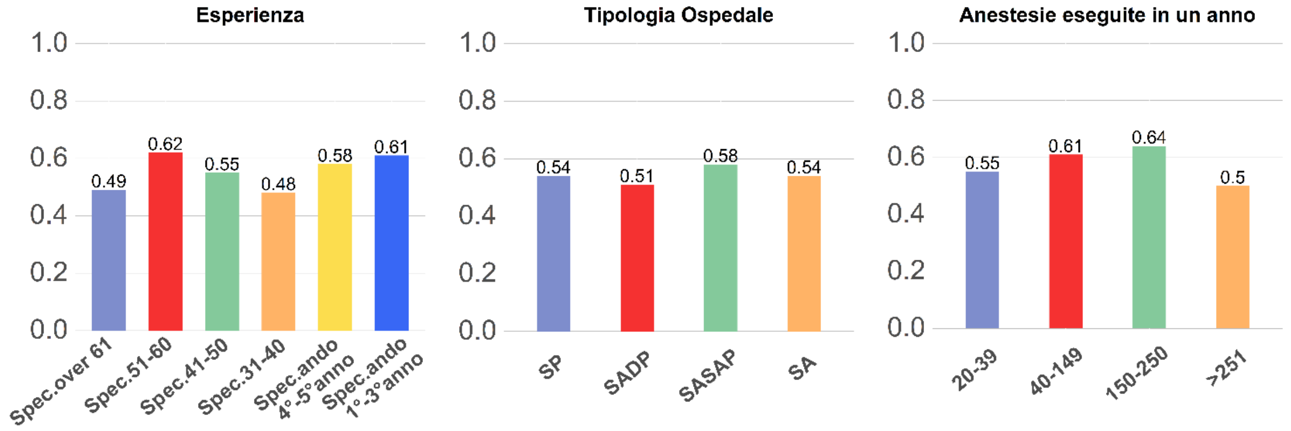

Esperienza	Fortemente disaccordo	Disaccordo	Né d'accordo né disaccordo	D'accordo	Fortemente d'accordo	Indicatore
**Specialista over 61**	11,1%	33,3%	27,8%	27,8%	0,0%	**0,49**
**Specialista 51- 60**	3,1%	31,2%	15,6%	46,9%	3,1%	**0,62**
**Specialista 41- 50**	5,3%	44,0%	10,7%	36,0%	4,0%	**0,55**
**Specialista 31- 40**	10,9%	40,0%	23,6%	20,0%	5,5%	**0,48**
**Specializzando 4°-5° anno**	0,0%	55,6%	16,7%	27,8%	0,0%	**0,58**
**Specializzando 1°-3° anno**	4,2%	29,2%	25,0%	33,3%	8,3%	**0,61**

Struttura	Fortemente disaccordo	Disaccordo	Né d'accordo né disaccordo	D'accordo	Fortemente d'accordo	Indicatore
**Pediatrica**	11,6%	34,9%	9,3%	34,9%	9,3%	**0,54**
**Per adulti con dipartimento pediatrico**	6,2%	45,3%	17,2%	31,2%	0,0%	**0,51**
**Per adulti con sporadica attività pediatrica**	2,5%	40,5%	20,3%	32,9%	3,8%	**0,58**
**Esclusivamente per adulti**	8,6%	31,4%	25,7%	28,6%	5,7%	**0,54**

N. Anestesie Annue	Fortemente disaccordo	Disaccordo	Né d'accordo né disaccordo	D'accordo	Fortemente d'accordo	Indicatore
**20-39**	5,0%	38,8%	24,0%	28,1%	4,1%	**0,55**
**40-149**	0,0%	46,2%	19,2%	34,6%	0,0%	**0,61**
**150-250**	0,0%	50,0%	0,0%	50,0%	0,0%	**0,64**
**>251**	15,1%	34,0%	11,3%	32,1%	7,5%	**0,5**

Gli anestesisti sembrerebbero presentare un giudizio discordante sull'assunzione di analgesici oppioidi nel periodo intraoperatorio. Gli anestesisti che lavorano nelle diverse tipologie di ospedali. Sembrerebbero avere giudizi discordanti sull'assunzione di analgesici oppioidi nel periodo intraoperatorio.

5- Nel bambino affetto da OSAs è preferibile non utilizzare anestetici inalatori
6-

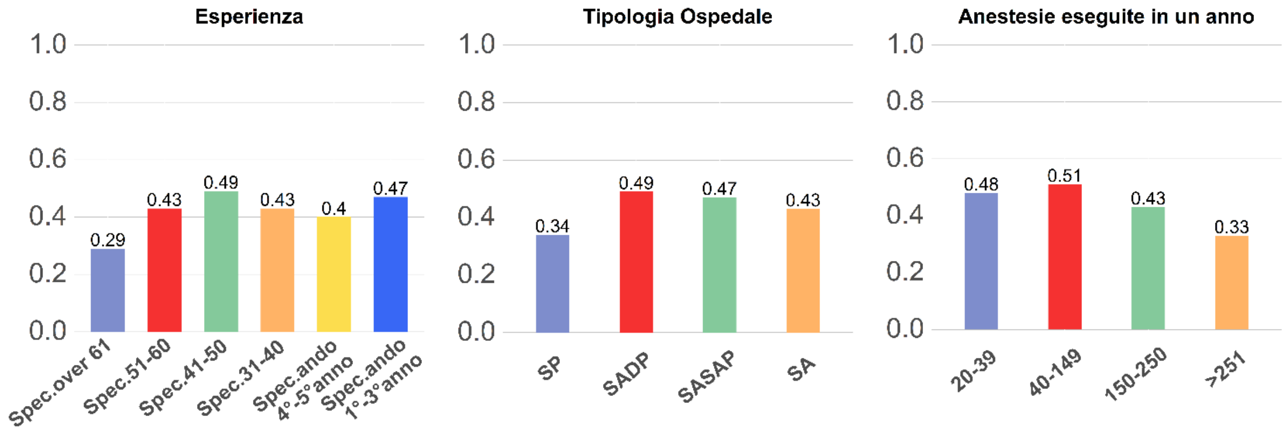

Esperienza	Fortemente disaccordo	Disaccordo	Né d'accordo né disaccordo	D'accordo	Fortemente d'accordo	Indicatore
**Specialista over 61**	26,3%	52,6%	15,8%	5,3%	0,0%	**0,29**
**Specialista 51- 60**	12,9%	38,7%	35,5%	12,9%	0,0%	**0,43**
**Specialista 41- 50**	10,8%	37,8%	24,3%	21,6%	5,4%	**0,49**
**Specialista 31- 40**	10,9%	47,3%	27,3%	14,5%	0,0%	**0,43**
**Specializzando 4°-5° anno**	16,7%	50,0%	16,7%	11,1%	5,6%	**0,40**
**Specializzando 1°-3° anno**	12,5%	25,0%	54,2%	8,3%	0,0%	**0,47**

Struttura	Fortemente disaccordo	Disaccordo	Né d'accordo né disaccordo	D'accordo	Fortemente d'accordo	Indicatore
**Pediatrica**	25,6%	44,2%	18,6%	7,0%	4,7%	**0,34**
**Per adulti con dipartimento pediatrico**	6,2%	43,8%	31,2%	17,2%	1,6%	**0,49**
**Per adulti con sporadica attività pediatrica**	9,0%	44,9%	26,9%	16,7%	2,6%	**0,47**
**Esclusivamente per adulti**	20,0%	22,9%	40,0%	17,1%	0,0%	**0,43**

N. Anestesie Annue	Fortemente disaccordo	Disaccordo	Né d'accordo né disaccordo	D'accordo	Fortemente d'accordo	Indicatore
**20-39**	9,2%	37,0%	37,0%	16,0%	0,8%	**0,48**
**40-149**	7,7%	46,2%	11,5%	30,8%	3,8%	**0,51**
**150-250**	13,0%	43,5%	30,4%	8,7%	4,3%	**0,43**
**>251**	24,5%	47,2%	17,0%	7,5%	3,8%	**0,33**

Gli intervistati sembrerebbero presentare un giudizio discordante nell'utilizzo di anestetici inalatori nel bambino affetto da OSAs.

7- Nei bambini affetti da OSAs è preferibile evitare il blocco neuromuscolare

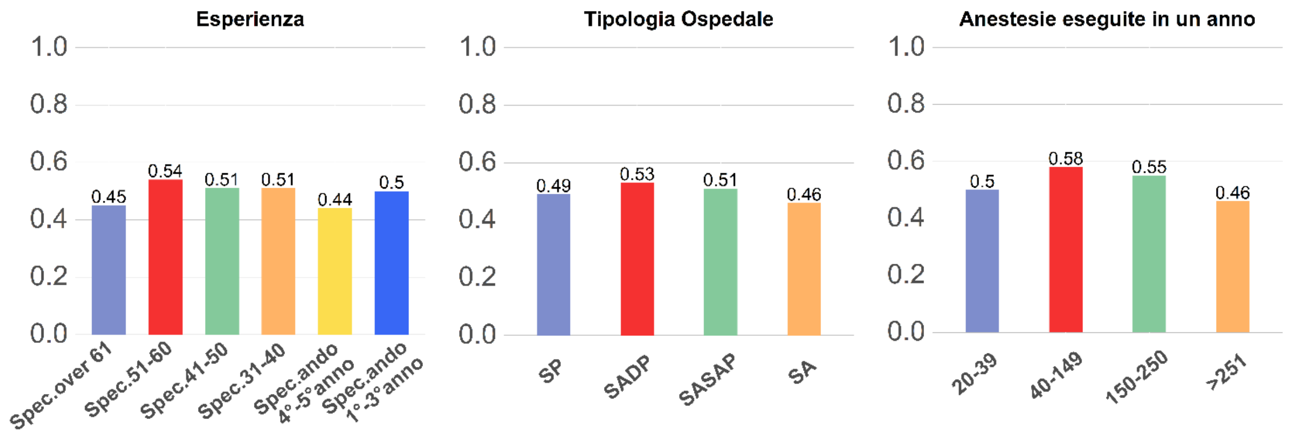

Esperienza	Fortemente disaccordo	Disaccordo	Né d'accordo né disaccordo	D'accordo	Fortemente d'accordo	Indicatore
**Specialista over 61**	15,8%	36,8%	21,1%	21,1%	5,3%	**0,45**
**Specialista 51- 60**	3,1%	43,8%	25,0%	28,1%	0,0%	**0,54**
**Specialista 41- 50**	9,5%	36,5%	21,6%	29,7%	2,7%	**0,51**
**Specialista 31- 40**	7,3%	38,2%	29,1%	21,8%	3,6%	**0,51**
**Specializzando 4°-5° anno**	11,1%	50,0%	16,7%	22,2%	0,0%	**0,44**
**Specializzando 1°-3° anno**	12,5%	25,0%	41,7%	16,7%	4,2%	**0,50**

Struttura	Fortemente disaccordo	Disaccordo	Né d'accordo né disaccordo	D'accordo	Fortemente d'accordo	Indicatore
**Pediatrica**	14,0%	32,6%	20,9%	30,2%	2,3%	**0,49**
**Per adulti con dipartimento pediatrico**	6,2%	35,9%	32,8%	21,9%	3,1%	**0,53**
**Per adulti con sporadica attività pediatrica**	7,6%	43,0%	20,3%	25,3%	3,8%	**0,51**
**Esclusivamente per adulti**	11,4%	37,1%	31,4%	20,0%	0,0%	**0,46**

N. Anestesie Annue	Fortemente disaccordo	Disaccordo	Né d'accordo né disaccordo	D'accordo	Fortemente d'accordo	Indicatore
**20-39**	8,3%	39,2%	29,2%	20,8%	2,5%	**0,5**
**40-149**	3,8%	38,5%	15,4%	38,5%	3,8%	**0,58**
**150-250**	8,7%	30,4%	21,7%	34,8%	4,3%	**0,55**
**>251**	13,2%	37,7%	24,5%	22,6%	1,9%	**0,46**

Gli anestesisti sembrerebbero presentare un giudizio discordante nell'evitare il blocco neuromuscolare nei bambini affetti da OSAs.

Gestione del post-operatorio/post-proceduta

8- In caso di pOSAs sospetta è opportuno monitorare i parametri vitali per 24h dopo una proceduta in sedazione/AG

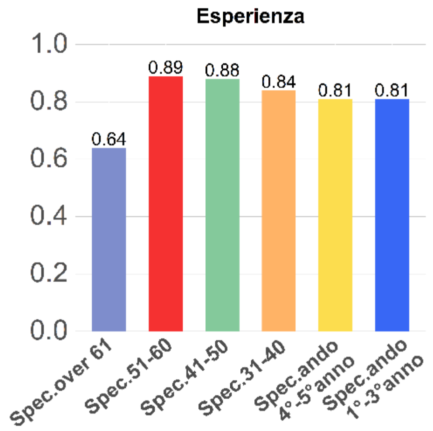

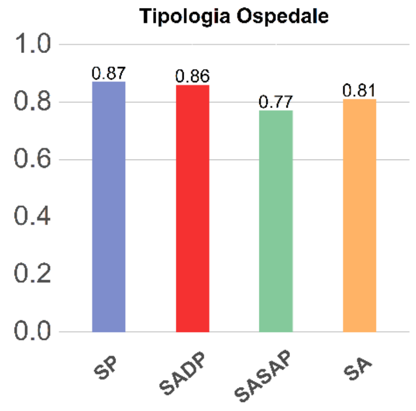

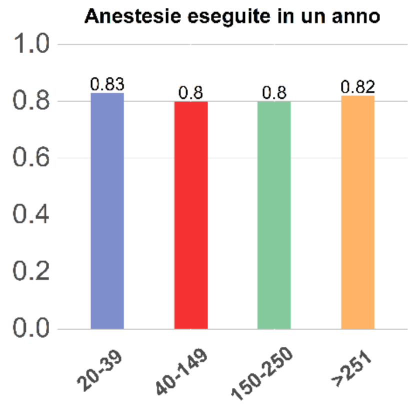

Esperienza	Fortemente disaccordo	Disaccordo	Né d'accordo né disaccordo	D'accordo	Fortemente d'accordo	Indicatore
**Specialista over 61**	10,5%	26,3%	0,0%	36,8%	26,3%	**0,64**
**Specialista 51- 60**	0,0%	3,1%	6,2%	65,6%	25,0%	**0,89**
**Specialista 41- 50**	0,0%	1,3%	14,7%	60,0%	24,0%	**0,88**
**Specialista 31- 40**	0,0%	7,3%	12,7%	60,0%	20,0%	**0,84**
**Specializzando 4°-5° anno**	5,6%	0,0%	11,1%	61,1%	22,2%	**0,81**
**Specializzando 1°-3° anno**	0,0%	4,2%	33,3%	45,8%	16,7%	**0,81**

Struttura	Fortemente disaccordo	Disaccordo	Né d'accordo né disaccordo	D'accordo	Fortemente d'accordo	Indicatore
**Pediatrica**	0,0%	2,3%	14,0%	60,5%	23,3%	**0,87**
**Per adulti con dipartimento pediatrico**	0,0%	3,1%	14,1%	60,9%	21,9%	**0,86**
**Per adulti con sporadica attività pediatrica**	2,5%	10,0%	12,5%	53,7%	21,2%	**0,77**
**Esclusivamente per adulti**	2,9%	2,9%	14,3%	54,3%	25,7%	**0,81**

N. Anestesie Annue	Fortemente disaccordo	Disaccordo	Né d'accordo né disaccordo	D'accordo	Fortemente d'accordo	Indicatore
**20-39**	0,8%	5,0%	12,4%	62,8%	19,0%	**0,83**
**40-149**	0,0%	11,5%	19,2%	46,2%	23,1%	**0,8**
**150-250**	4,3%	4,3%	8,7%	56,5%	26,1%	**0,8**
**>251**	1,9%	3,8%	15,1%	50,9%	28,3%	**0,82**

Tutti gli specialisti, a differenza dello specialista over 61, sembrerebbero concordare nel monitorare per 24h i parametri vitali dopo una procedura in sedazione/AG in caso di pOSAs sospetta. Gli anestesisti che lavorano in strutture pediatriche o strutture per adulti con dipartimento pediatrico sembrerebbero concordare nel monitorare per 24h i parametri vitali dopo una procedura in sedazione/AG in caso di pOSAs sospetta. Tutti gli anestesisti che hanno effettuato almeno 20 anestesie all'anno sembrerebbero concordare nel monitorare per 24h i parametri vitali dopo una procedura in sedazione/AG in caso di pOSAs sospetta.

9- L'analgesia postoperatoria con oppioidi deve prevedere dosaggi ridotti in caso di pOSAs moderata/severa

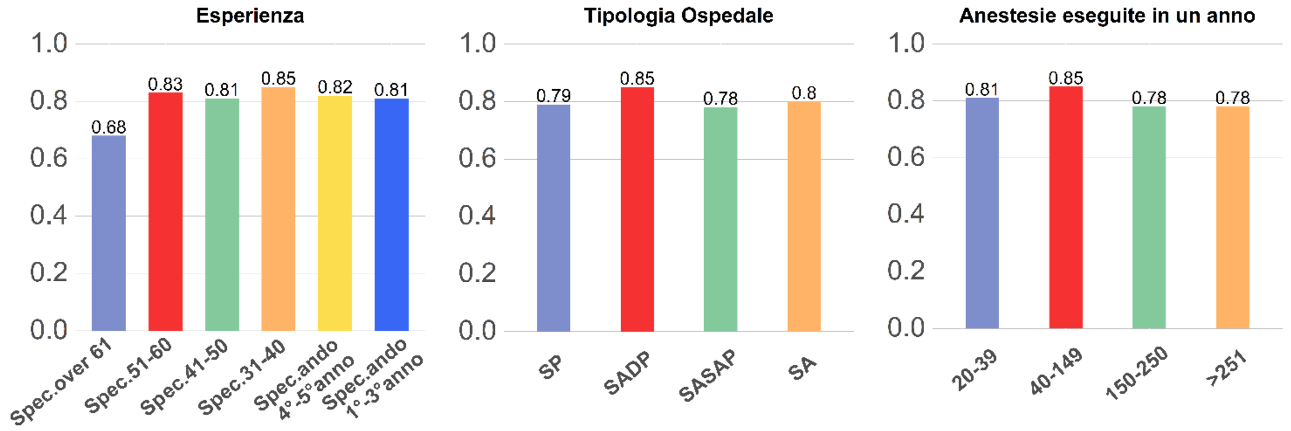

Esperienza	Fortemente disaccordo	Disaccordo	Né d'accordo né disaccordo	D'accordo	Fortemente d'accordo	Indicatore
**Specialista over 61**	10,5%	5,3%	21,1%	47,4%	15,8%	**0,68**
**Specialista 51- 60**	0,0%	9,4%	15,6%	43,8%	31,2%	**0,83**
**Specialista 41- 50**	2,7%	6,7%	10,7%	50,7%	29,3%	**0,81**
**Specialista 31- 40**	0,0%	3,7%	16,7%	61,1%	18,5%	**0,85**
**Specializzando 4°-5° anno**	0,0%	11,1%	11,1%	61,1%	16,7%	**0,82**
**Specializzando 1°-3° anno**	0,0%	8,3%	20,8%	58,3%	12,5%	**0,81**

Struttura	Fortemente disaccordo	Disaccordo	Né d'accordo né disaccordo	D'accordo	Fortemente d'accordo	Indicatore
**Pediatrica**	2,3%	7,0%	14,0%	53,5%	23,3%	**0,79**
**Per adulti con dipartimento pediatrico**	0,0%	6,2%	12,5%	59,4%	21,9%	**0,85**
**Per adulti con sporadica attività pediatrica**	2,5%	8,8%	15,0%	48,8%	25,0%	**0,78**
**Esclusivamente per adulti**	2,9%	2,9%	17,6%	55,9%	20,6%	**0,8**

N. Anestesie Annue	Fortemente disaccordo	Disaccordo	Né d'accordo né disaccordo	D'accordo	Fortemente d'accordo	Indicatore
**20-39**	0,8%	8,3%	13,3%	55,8%	21,7%	**0,81**
**40-149**	0,0%	3,8%	19,2%	50,0%	26,9%	**0,85**
**150-250**	4,3%	4,3%	17,4%	47,8%	26,1%	**0,78**
**>251**	3,8%	5,7%	15,1%	52,8%	22,6%	**0,78**

Tutti gli specialisti, a differenza dello specialista over 61, sembrerebbero concordare nel monitorare per 24h i parametri vitali dopo una procedura in sedazione/AG in caso di pOSAs sospetta.

10- Team Work e gestione multidisciplinare

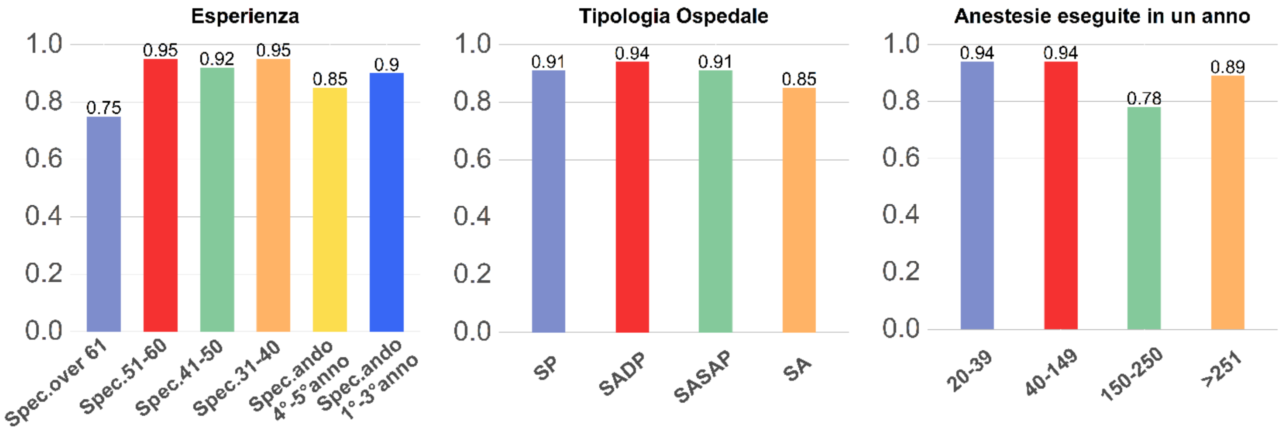

Esperienza	Fortemente disaccordo	Disaccordo	Né d'accordo né disaccordo	D'accordo	Fortemente d'accordo	Indicatore
**Specialista over 61**	10,5%	0,0%	15,8%	42,1%	31,6%	**0,75**
**Specialista 51- 60**	0,0%	0,0%	3,1%	46,9%	50,0%	**0,95**
**Specialista 41- 50**	1,3%	0,0%	1,3%	52,0%	45,3%	**0,92**
**Specialista 31- 40**	0,0%	0,0%	3,6%	40,0%	56,4%	**0,95**
**Specializzando 4°-5° anno**	5,6%	0,0%	5,6%	44,4%	44,4%	**0,85**
**Specializzando 1°-3° anno**	10,5%	0,0%	15,8%	42,1%	31,6%	**0,75**

Struttura	Fortemente disaccordo	Disaccordo	Né d'accordo né disaccordo	D'accordo	Fortemente d'accordo	Indicatore
**Pediatrica**	2,3%	0,0%	0,0%	55,8%	41,9%	**0,91**
**Per adulti con dipartimento pediatrico**	0,0%	0,0%	4,7%	51,6%	43,8%	**0,94**
**Per adulti con sporadica attività pediatrica**	1,3%	0,0%	7,5%	40,0%	51,2%	**0,91**
**Esclusivamente per adulti**	5,7%	2,9%	2,9%	34,3%	54,3%	**0,85**

N. Anestesie Annue	Fortemente disaccordo	Disaccordo	Né d'accordo né disaccordo	D'accordo	Fortemente d'accordo	Indicatore
**20-39**	0%	1%	5%	41%	53%	**0,94**
**40-149**	0%	0%	4%	54%	42%	**0,94**
**150-250**	9%	0%	9%	57%	26%	**0,78**
**>251**	4%	0%	2%	45%	49%	**0,89**

Tutti gli specialisti sembrerebbero concordare nel mettere in pratica il Team Work e gestione multidisciplinare per la gestione del post-operatorio /post procedura. Tutti gli anestesisti che lavorano nelle

diverse tipologie di ospedale prese a riferimento, sembrerebbero concordare mettere in pratica il Team Work e gestione multidisciplinare per la gestione del post-operatorio /post procedura.

RISERVATO
